# Analysis of durum wheat photosynthetic organs during grain filling reveals the ear as a water stress-tolerant organ and the peduncle as the largest pool of primary metabolites

**DOI:** 10.1007/s00425-023-04115-1

**Published:** 2023-03-14

**Authors:** Raquel Martínez-Peña, Omar Vergara-Díaz, Armin Schlereth, Melanie Höhne, Rosa Morcuende, María Teresa Nieto-Taladriz, José Luis Araus, Nieves Aparicio, Rubén Vicente

**Affiliations:** 1 Cereals Group, Section of Herbaceous, Agro-Technological Institute of Castile and León, Junta de Castile and León, Valladolid, Spain; 2 Plant Ecophysiology and Metabolism Group, Instituto de Tecnologia Química e Biológica António Xavier, Universidade Nova de Lisboa (ITQB NOVA), Oeiras, Portugal; 3 Max Planck Institute of Molecular Plant Physiology, Potsdam, Germany; 4 Institute of Natural Resources and Agrobiology of Salamanca (IRNASA), Consejo Superior de Investigaciones Científicas (CSIC), Salamanca, Spain; 5 Instituto Nacional de Investigación y Tecnología Agraria y Alimentaria (INIA), Consejo Superior de Investigaciones Científicas, Madrid, Spain; 6 Integrative Crop Ecophysiology Group, Section of Plant Physiology, Faculty of Biology, University of Barcelona, Barcelona, and AGROTECNIO-CERCA Center, Lleida, Spain

**Keywords:** Carbon metabolism, Drought, Field trial, Mediterranean region, Nitrogen metabolism, Source–sink dynamics

## Abstract

**Main conclusion:**

The pool of carbon- and nitrogen-rich metabolites is quantitatively relevant in non-foliar photosynthetic organs during grain filling, which have a better response to water limitation than flag leaves.

**Abstract:**

The response of durum wheat to contrasting water regimes has been extensively studied at leaf and agronomic level in previous studies, but the water stress effects on source–sink dynamics, particularly non-foliar photosynthetic organs, is more limited. Our study aims to investigate the response of different photosynthetic organs to water stress and to quantify the pool of carbon and nitrogen metabolites available for grain filling. Five durum wheat varieties were grown in field trials in the Spanish region of Castile and León under irrigated and rainfed conditions. Water stress led to a significant decrease in yield, biomass, and carbon and nitrogen assimilation, improved water use efficiency, and modified grain quality traits in the five varieties. The pool of carbon (glucose, glucose-6-phosphate, fructose, sucrose, starch, and malate) and nitrogen (glutamate, amino acids, proteins and chlorophylls) metabolites in leaf blades and sheaths, peduncles, awns, glumes and lemmas were also analysed. The results showed that the metabolism of the blades and peduncles was the most susceptible to water stress, while ear metabolism showed higher stability, particularly at mid-grain filling. Interestingly, the total metabolite content per organ highlighted that a large source of nutrients, which may be directly involved in grain filling, are found outside the blades, with the peduncles being quantitatively the most relevant. We conclude that yield improvements in our Mediterranean agro-ecosystem are highly linked to the success of shoots in producing ears and a higher number of grains, while grain filling is highly dependent on the capacity of non-foliar organs to fix CO_2_ and N. The ear organs show higher stress resilience than other organs, which deserves our attention in future breeding programmes.

**Supplementary Information:**

The online version contains supplementary material available at 10.1007/s00425-023-04115-1.

## Introduction

There is evidence of a fall in production in major crops such as wheat and maize in many regions like the Mediterranean basin due to climate change, i.e. increases in temperature and the occurrence of severe droughts and extreme events (Lobell et al. 2011). This is particularly relevant when the temperature increases take place in May–July, because such timing is negatively associated with wheat yields in Europe (Pinke et al. 2022). Moreover, future exacerbation of climate change effects, together with growing population and changes in dietary habits, will further endanger crop yield stability particularly in the countries of the Mediterranean basin. Thus, plant scientists are motivated to provide new strategies and explore potential sources of crop stress resilience to enhance food security.

Advances in field-based high-throughput phenotyping have undoubtedly contributed to a better understanding of the genotype-by-environment interaction, making it possible to evaluate the physiological and yield performance in extensive collections of varieties (Araus et al. 2022). However, such robust phenotyping systems combined with traditional breeding programmes may not be sufficient to ensure future food security. To complement them and increase the annual genetic gains, novel traits with high genetic variation are needed. This is particularly critical for important crops like bread and durum wheat, whose genetic advances have stagnated in the last few decades, particularly in southern European countries (Chairi et al. 2018; Pinke et al. 2022).

Durum wheat is one of the most relevant Mediterranean food crops in terms of cultivated area (about 16 M hectares) and nutritional importance, e.g. production of pasta, couscous and bulgur (Guzmán et al. 2016; Beres et al. 2020). While its production is concentrated in the south and east of the Mediterranean basin, it is also cultivated in the North American Great Plains, Russia and Kazakhstan (Tidiane Sall et al. 2019; Royo et al. 2021). In these regions, durum wheat is usually subjected to terminal drought during the highly sensitive stages of anthesis and grain filling, which compromises grain yield. During water deprivation events, plant growth is inhibited as a result of restricted CO_2_ diffusion (i.e. stomatal closure) and a drop in photosynthesis and N assimilation (Tezara et al. 1999; Xu and Yu 2006). Water stress triggers major transcriptional reprogramming of plant metabolism, including signalling, which leads to cell osmotic adjustment, e.g. accumulation of certain sugars and amino acids as osmoprotectants and an antioxidant response (Ergen et al. 2009; Rybka and Nita 2015; Ullah et al. 2017; Vicente et al. 2018b). Prolonged stress, mainly occurring at sensitive growth stages, may lead to metabolic disruption and collapse, affecting grain yield severely (Rybka and Nita 2015). These effects of water stress cause changes in grain quality traits, usually increasing levels of protein, bioactive compounds and minerals (De Santis et al. 2021).

Yield is a complex trait that depends on the agronomic components (e.g. ears per area, grains per ear and grain weight) and the physiological traits, such as the amount and efficiency of resource use (water, nutrients and light). Grain yield can be limited by sink and source organs in terms of either storing or assimilation capacity (Reynolds et al. 2022). Grain filling is considered to be mainly limited by the ability of the developing grains to utilise the translocated assimilates (sink limitation), but source and sink co-limitations may also exist (Slafer and Savin 1994; Rivera-Amado et al. 2020; Reynolds et al. 2022). Under water stress, grain filling is mainly limited by source capacity due to the inhibition of C fixation and N assimilation, which have been mainly reported in the flag leaves (Medina et al. 2016; Vicente et al. 2018b). Photoassimilates originate from different organs and pathways, such as the photosynthesis that occurs in the leaves (blades and sheaths) and ears (mainly glumes, lemmas, and awns), as well as translocation from internodes (Sanchez-Bragado et al. 2020; Tambussi et al. 2021). The contribution of each of these compartments to grain filling is complex because it depends on the environmental conditions, species, variety and phenology.

The leaf blade has traditionally been considered the primary source of photoassimilates during optimal and stress conditions in wheat, especially the flag leaf during late developmental stages (Hu et al. 2018; Tambussi et al. 2021), whereas relatively little attention has been paid to the performance of other foliar or non-foliar photosynthetic organs. For instance, peduncles and leaf sheaths are organs that show active photosynthesis, delayed senescence and play an important role in sugar storage (Schnyder 1993; Kong et al. 2010; Martínez-Peña et al. 2022). One of the first detailed reports about ear photosynthesis estimated its contribution to grain yield as being 10–44% (Kriedemann 1966). In the last few years, far more attention has been paid to the metabolic performance of the wheat ear and its contribution to grain filling (Sanchez-Bragado et al. 2020). It is worth mentioning that the significant role of this organ has been corroborated using different methodologies such as organ removal and shadowing (Maydup et al. 2012; Sanchez-Bragado et al. 2016), isotopic and nutrient composition determinations in different organs (Sanchez-Bragado et al. 2014a, 2014b, 2017), as well as transcriptional and metabolic approaches (Lopes et al. 2006; Jia et al. 2015; Vicente et al. 2018b; Shokat et al. 2020; Vergara-Diaz et al. 2020; Martínez-Peña et al. 2022). Interestingly, it has also been suggested that the contribution of ear tissues may be even more relevant under stress conditions (Hu et al. 2018; Sanchez-Bragado et al. 2020).

Compared to other organs, ears may be considered as organs that are constitutively adapted to stress due to their xeromorphic anatomy, more stable N and water status and C and N assimilation (Araus et al. 1993, 2003; Martinez et al. 2003; Lopes et al. 2006; Vicente et al. 2018b; Sanchez-Bragado et al. 2020). While some studies have reported that awns are the most relevant CO_2_-fixing tissues in the ears, others have highlighted that glumes and lemmas may serve as essential refixation sites of the endosperm-respired C in addition to fixing atmospheric CO_2_ (Araus et al. 1992; Bort et al. 1996; Sanchez-Bragado et al. 2020; Tambussi et al. 2021). Under water stress, respiration and N metabolism may be up-regulated and senescence delayed in the ears compared to leaves (Martinez et al. 2003; Hu et al. 2018; Vicente et al. 2018b). Finally, respiration and photorespiration intermediates and specific amino acids have been reported to increase in ear bracts (glumes + lemmas) relative to leaves, highlighting better coordination of C and N metabolism in these particular tissues (Vergara-Diaz et al. 2020). There is, however, some degree of uncertainty about the real activity and potential of ear organs, as summarised in Araus et al. (2021). For instance, methodological issues such as whether to consider some of these traits at the whole organ level vs. on a per area basis may greatly affect the conclusions.

The complexity of encompassing different analytical (plant agronomy, physiology, and biochemistry), temporal (plant growth development), morphological (plant organs) and environmental (growth conditions) scales and including a large germplasm collection has been a limitation in previous studies to understanding plant metabolism at the whole-plant level and its contribution to yield traits. While some earlier studies have analysed the metabolic response of different organs to stress (Jia et al. 2015; Kong et al. 2015; Hu et al. 2018; Sanchez-Bragado et al. 2020; Vergara-Diaz et al. 2020; Tambussi et al. 2021; Dehigaspitiya et al. 2022; Martínez-Peña et al. 2022), the current work deepens our understanding of the source–sink dynamics of C and N metabolites in field-grown durum wheat in response to water stress. We analysed the levels of key C and N metabolites in the most relevant photosynthetic organs at the grain filling stage in five varieties widely used on the Iberian Peninsula in recent decades. Special attention is paid to phenology-dependent dynamics and the identification of new traits as selection criteria for developing climate-resilient crops in future breeding programs.

## Materials and methods

### Plant material and experimental design

**Fig. 1:**
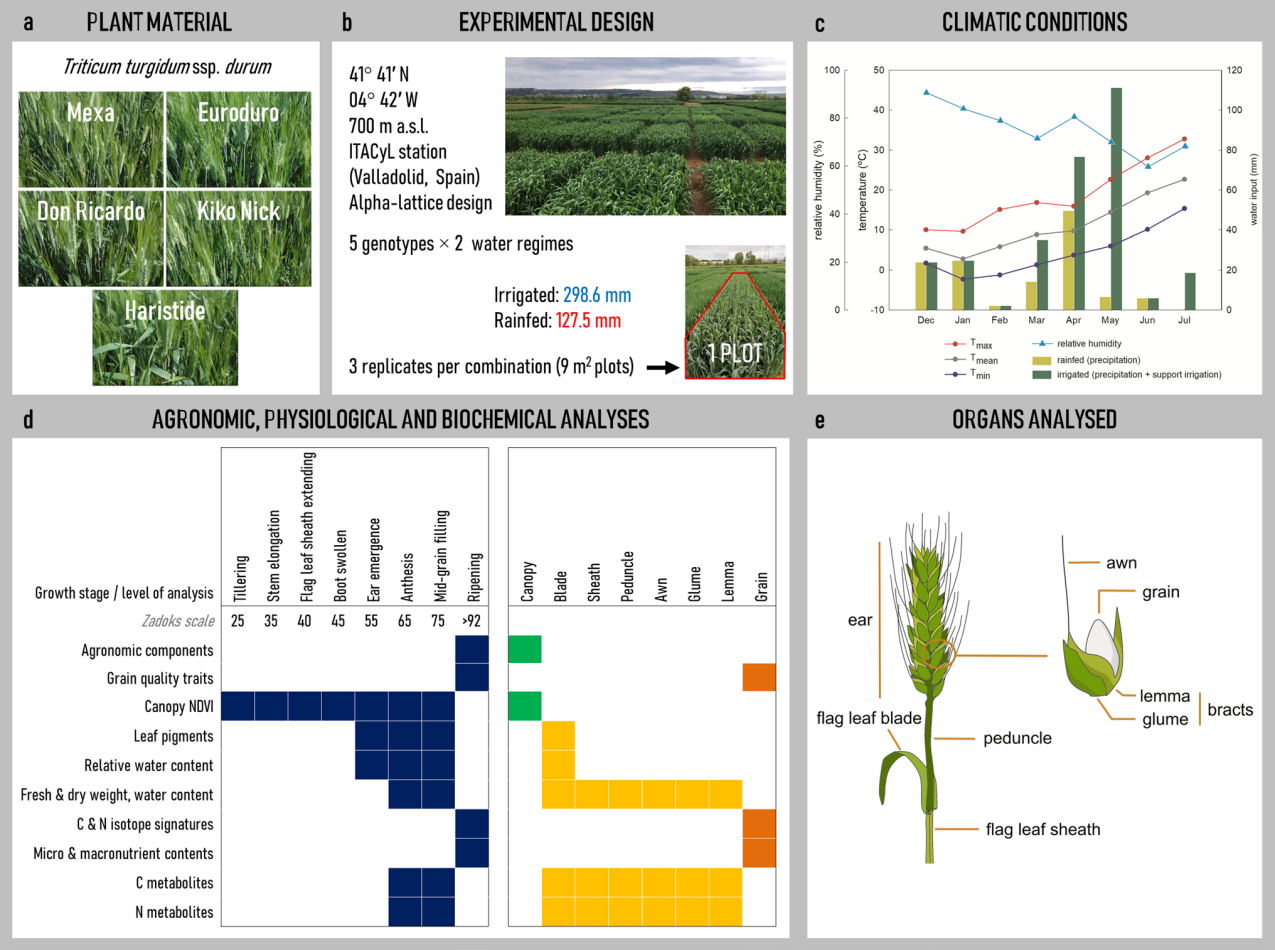
Scheme of the experimental setup and analyses carried out. **a** Five durum wheat varieties were **b** grown under two water regimes (irrigated and rainfed conditions). **c** Climatic conditions during the 2018/2019 crop season are shown: daily maximum (red line), mean (grey line), and minimum (dark blue line) temperatures, relative humidity (light blue line), and water inputs (precipitation as light green bars, and with support irrigation as dark green bars). **d** A detailed list with the protocols performed at different growth stages and plant levels is included, **e** with an emphasis on the sampled organs

Five modern durum wheat [*Triticum turgidum* L. ssp. *durum* (Desf.)] varieties were grown during the 2018/2019 crop season: Mexa, Euroduro, Don Ricardo, Kiko Nick and Haristide, released after the Green Revolution in 1980, 2007, 2008, 2009 and 2015, respectively (Fig. 1a). These varieties were selected due to their wide cultivation and high yield performance during recent decades in the Mediterranean region of Castile and León (Spain). Mexa is a historical CIMMYT variety that dominated the market in the 1980s; Don Ricardo is a high-yielding and widely commercialised variety in southern Spain, where the largest durum wheat area in the country is located; and, these two varieties together with Kicko Nick, Euroduro and Haristide, have given good agronomic results in previous crop seasons in the study region (Chairi et al. 2018; Gracia-Romero et al. 2019; Martínez-Peña et al. 2022). The field trials were carried out at the Zamadueñas experimental station of the Agro-technological Institute of Castile and León (ITACyL), located in Valladolid, Spain (41° 41′ N, 04º 42′ W, 700 m a.s.l.; Fig. 1b). The climate is continental Mediterranean, and the soil is xerofluvent with a loamy sand texture; the upper 0.30 m had 13.9 g kg^−1^ of organic matter, 66.4 g kg^−1^ of carbonate, 0.81 g kg^−1^ of N, 0.27 g kg^−1^ of phosphorus, a pH of 8.5 and electric conductivity of 0.123 dS m^−1^. Meteorological data were collected with an automated meteorological station located in the experimental station. The maximum, average and minimum temperatures throughout the crop season were 18.9, 11.1, and 4.3 °C, respectively, with 75.4% mean relative humidity and 127.5 mm of accumulated precipitation (Fig. 1c). Sowing was carried out on 3rd December 2018 at a seed rate of 250 seeds m^−2^. Before sowing, the field trial received a basal application of 300 kg ha^−1^ of 8-15-15 NPK fertiliser on 16th November 2018. Then, the trial was dressed with N applied at the beginning of tillering (28th February 2019) and jointing (12th April 2019), using a dose of 150 kg ha^−1^ of calcium ammonium nitrate (CAN 27%) and 150 kg ha^−1^ of ammonium nitrosulphate (ASN 26%).

Two water regimes were imposed: the control treatment (irrigated) and the stress treatment (rainfed), the latter depending exclusively on the rainfall. The irrigated plots received 171.1 mm of additional water, provided as sprinkler irrigations between 29th March and 31st May 2019, and alongside precipitation these accounted for a total of 298.6 mm. The experimental design was an alpha-lattice design with three replicates per variety and water regime. The size of each plot was 6 m long by 1.5 m wide (9 m^2^), with six rows and 0.25 m of space between them (Fig. 1b). Weeds, insect pests and diseases were controlled by applying the recommenced agrochemicals to avoid yield limitations.

Harvesting was performed after the crop reached physiological maturity on the 3rd (rainfed) and 15th of July 2019 (irrigated). Phenotyping was carried out on sunny days around noon (11–14 h, UTC + 1) on the whole canopy (greenness) and at the single leaf level (pigments) in every plot at different developmental stages throughout the crop cycle (Fig. 1d). In addition, different foliar and non-foliar green organs (flag leaf blades and sheaths, peduncles, awns, glumes and lemmas; Fig. 1e) were sampled at key reproductive stages for further analyses (relative leaf water content, organ water content, dry weight, and carbon and nitrogen primary metabolites). For each water regime, cultivar, plot and date of sampling, a pool of five plants was sampled, separated into the different organs and considered as a biological replicate.

### Agronomic components and grain quality traits

At maturity, two 0.5 m-length row samples were collected randomly from the central rows of each plot a few days before harvest, and were processed as dry matter after drying at 70 ºC for 24 h to determine the aboveground biomass. After that, the number of plants and ears per m^2^ was calculated by counting the plants and ears contained in the combined one metre-length sample. In addition, the number of ears per plant was also measured. Then, the number of grains per ear and the thousand kernels weight (TKW) were determined in a representative subset of ten main shoots per plot of the previous selection. At the end of the experiment, the 9 m^2^ plots were harvested mechanically, and the moisture content of the grain was determined with a PM-450 grain moisture tester (Kett, Villa Park, CA, USA). The grain yield was obtained for each plot and adjusted to a 10% moisture basis. Finally, the harvest index (HI) was calculated from the agronomic components obtained:

$$\text{Harvest Index}=\text{grain weight}/(\text{grain weight}+\text{biomass}).$$

A pool of 250 g of mature grains per plot was collected to assess the grain quality traits. First, protein content was determined using an Infratec 1226 grain analyzer (Foss Analytical, Hillerød, Denmark) on a dry basis. Test weight (TW) was determined according to AACC Method 55-10. The percentage of vitreous grains (vitreousness) was evaluated in two batches containing 100 seeds each. Whole-grain flour samples were obtained after using an LM 3100 mill (Perten Instruments AB, Hägersten, Sweden) and later used to evaluate the gluten strength by the sodium dodecyl sulphate sedimentation (SDSS) test (Axford et al. 1978), and the yellow colour index (b*, according to the CIE L*a*b* colour system) using a portable reflectance colorimeter (CR-310, Konica-Minolta Sensing Inc, Tokyo, Japan). Finally, wet gluten (WG) and the gluten index (GI) were determined according to ICC Methods 155 and 158, respectively.

### Canopy NDVI and leaf pigments

We measured the normalised difference vegetation index (NDVI) at the canopy level using a GreenSeeker hand-held portable spectroradiometer (NTech Industries, Ukiah, CA, USA) during the growing cycle at tillering, stem elongation, flag leaf sheath extension, boot swollen, ear emergence, and anthesis, corresponding to Zadoks stages 25, 35, 40, 45, 55 and 65, respectively (Zadoks et al. 1974). The NDVI was calculated using the equation: NDVI = (NIR − R)/(NIR + R), where R and NIR are the reflectances in the red (660 nm) and near-infrared (760 nm) bands. The measurements were acquired at a distance of 0.5–0.6 m above the plants with the sensor perpendicular to the canopy. In addition, the relative content of chlorophylls (Chl), flavonoids and anthocyanins, and the N balanced index (NBI; estimated as the ratio of Chl and flavonoids) were obtained with a DUALEX leaf-clip sensor (ForceA, Orsay, France) at ear emergence, anthesis, and mid-grain filling (Zadoks 55, 65 and 75). The measurements were carried out in the middle of the flag leaf blades of five plants per plot selected randomly, which were the same plants collected for the biochemical analyses described below.

### Relative water content in flag leaves and fresh and dry weight per organ

Leaf relative water content (RWC) was determined according to Estévez-Geffriaud et al. (2020) at ear emergence, anthesis, and mid-grain filling (Zadoks stages 55, 65 and 75, respectively) using the fresh (FW), turgid (TW) and dry (DW) weights of flag leaf blades according to the following equation:

$$\mathrm{RWC }\left(\mathrm{\%}\right)=\left[\left(\mathrm{FW}-\mathrm{DW}\right)/\left(\mathrm{TW}-\mathrm{DW}\right)\right]\times 100.$$

Five plants per plot were collected at anthesis and mid-grain filling to calculate the FW, and after drying the samples in the oven at 70 °C for 48 h, the DW of the different organs (flag leaf blades and sheaths, peduncles, awns, glumes, lemmas and whole ears; Fig. 1e). The water content (WC) of the organs was calculated according to the following equation:

$$\mathrm{WC }\left(\mathrm{\%}\right)=\left[\left(\mathrm{FW}-\mathrm{DW}\right)/\mathrm{FW}\right]\times 100.$$

### Carbon and nitrogen isotope signatures and nutrient composition in grains

The mature grains, dried at 70ºC for 48 h, were finely ground and used to assess the stable C (δ^13^C) and N (δ^15^N) isotope composition and their contents. These analyses were carried out with a Flash 1112 EA elemental analyser (Thermo Finnigan, Bremen, Germany) coupled with a Delta C isotope ratio mass spectrometer (IRMS; Thermo Finnigan), at the Scientific-Technical Services of the University of Barcelona (Barcelona, Spain) as described in Medina et al. (2016). The C and N content in grains were used to calculate the grain C (GCY, kg ha^−1^) and N (GNY, kg ha^−1^) yields as follows:

$$\text{GCY}=\left[\text{grain C content }\left(\mathrm{\%}\right)\times \text{grain yield }(\text{kg }{\text{ha}}^{-1})\right]/100,$$

$$\text{GNY}=\left[\text{grain N content }\left(\mathrm{\%}\right)\times \text{grain yield }(\text{kg }{\text{ha}}^{-1})\right]/100.$$

Furthermore, the macronutrient and micronutrient contents (Ca, K, Mg, P, S, Cu, Fe, Mn, and Zn) were also analysed in the mature grains. For this, around 500 mg of dried material was mixed with 8 ml of 60% HNO_3_ and 2 ml of 30% H_2_O_2_ in a teflon container. The samples were subsequently digested at 200 °C in an ETHOUS UP microwave digestion system (Milestone, Sorisole, Italy). Afterwards, the solutions were cooled down at room temperature and diluted to 25 mL by adding deionised water. Finally, the nutrient concentration was determined at the Analysis and Instrumentation Service of the Institute of Natural Resources and Agrobiology of Salamanca (Salamanca, Spain) with a Varian 720-ES inductively coupled plasma-optical emission spectrometer (ICP-OES; Agilent Technologies, Santa Clara, CA, USA).

### Determination of carbon and nitrogen primary metabolites

Foliar and non-foliar green organs were harvested and placed in liquid N_2_ at anthesis and mid-grain filling (Zadoks 65 and 75, respectively) at noon on sunny days and subsequently stored at − 80 °C. Blades, sheaths and peduncles were ground to a fine powder in a Mixer Mill MM300 (Retsch GmbH, Haan, Germany), while awns, glumes and lemmas were carefully detached from several ears per plot and ground manually using a mortar and pestle with liquid N_2_. Glucose (Glc), glucose-6-phosphate (Glc6P), fructose (Fru), sucrose (Suc), starch, malate, glutamate (Glu), free amino acids (aa), total soluble proteins and Chl (a, b, and total) were determined in ethanolic extracts obtained from 20 mg of powder from every organ. First, the ethanolic extracts were obtained from aliquots using serial extractions with boiling ethanol as described by Stitt et al. (1989). Then, Chl a (Chla), b (Chlb) and total (Chltotal) were measured spectrophotometrically and calculated according to Lichtenthaler (1987). Second, the soluble sugars Glc, Fru, and Suc were determined according to Stitt et al. (1989) with the volumes adapted to 96-well plates, while Glc6P, malate and Glu were measured as previously described by Gibon et al. (2002) and Cross et al. (2006). The amino acids were also determined in the ethanolic extracts following the fluorescamine method described by Bantan-Polak et al. (2001) using a black microplate and Glu as standard. The starch and proteins were extracted and determined from the insoluble material remaining after the ethanolic extraction (Hendriks et al. 2003). All the assays were performed on 96-well plates using an ELx800 microplate reader (Bio-Tek, Winooski, VT, USA) at the Max Planck Institute of Molecular Plant Physiology (Potsdam, Germany). Two technical replicates were considered and the standards were included on each microplate as measuring references. Metabolites were calculated as a FW concentration and then expressed relative to both DW and total organ content (after multiplying by the weight of each organ).

### Statistical analysis

Statistical analyses were carried out with IBM SPSS v23.0 (SPSS Inc., Chicago, IL, USA) and R environment with the RStudio v2022.02.2-485 interface (R Foundation for Statistical Computing, Vienna, Austria). Two-way analyses of variance (ANOVA) were performed for all the variables to study the effects of genotype × water regime and organ × water regime. In addition, Tukey's Honestly Significant Difference (HSD) was used to test differences among group means for significance, which was accepted at *P* < 0.05. Line, scatter and bar plots were generated with SigmaPlot 12.0 (Systat Software Inc., Palo Alto, CA, USA) and the R package *ggplot2*; the principal component analyses (PCA) were undertaken with the *factoextra* and *FactoMineR* packages and the correlation matrix for agronomic components and grain quality traits were generated using the function *cor()* and the package *corrplot*.

## Results

### The impact of water regime on the performance of five durum wheat varieties

**Fig. 2:**
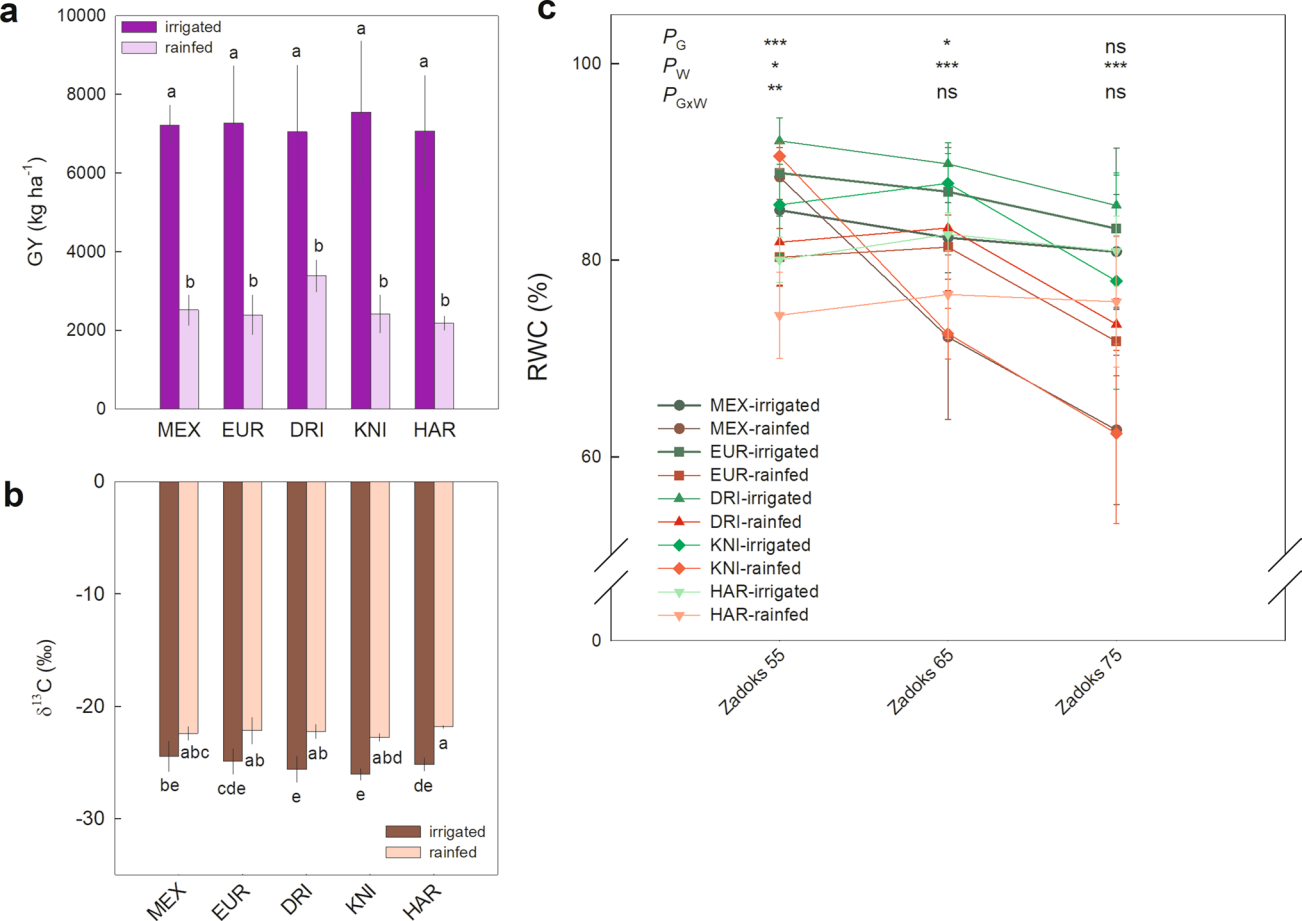
**a** Grain yield (GY), **b** C isotope composition (δ^13^C) in grains at harvest, and **c** relative water content (RWC) in flag leaves at ear emergence, anthesis and mid-grain filling (Zadoks 55, 65 and 75, respectively), in five durum wheat varieties (MEX, Mexa; EUR, Euroduro; DRI, Don Ricardo; KNI, Kiko Nick; HAR, Haristide) under rainfed and irrigated conditions. For each comparison of means, letters are significantly different (ns, not significant; *, *P* < 0.05; **, *P* < 0.01; ***, *P* < 0.001; two-way ANOVA, TUKEY test)

**Fig. 3:**
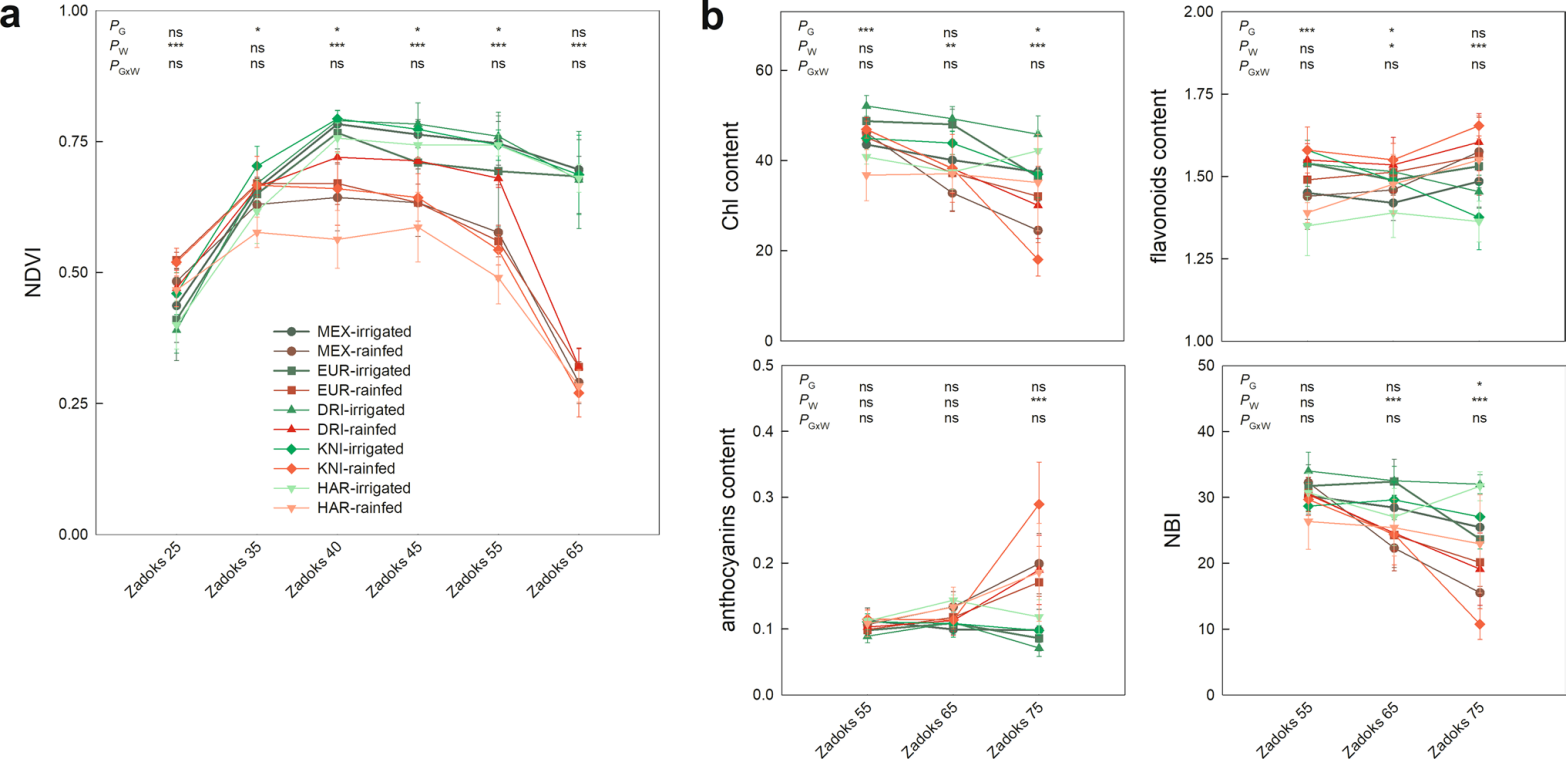
Spectral vegetation indices at **a** the canopy and **b** leaf levels in five durum wheat varieties (MEX, Mexa; EUR, Euroduro; DRI, Don Ricardo; KNI, Kiko Nick; HAR, Haristide) under rainfed and irrigated conditions. **a** A GreenSeeker spectroradiometer was used to determine the Normalised Difference Vegetation Index (NDVI) at six growth stages: tillering, stem elongation, flag leaf sheath extension, boot swollen, ear emergence, anthesis, and mid-grain filling (Zadoks 25, 35, 40, 45, 55 and 65, respectively); while **b** a DUALEX leaf-clip sensor estimated the chlorophyll (Chl), flavonoid, and anthocyanin contents and the N balanced index (NBI) at ear emergence, anthesis and mid-grain filling stages. All values are dimensionless, specific for each device. For each comparison of means, letters are significantly different (ns, not significant; *, *P* < 0.05; **, *P* < 0.01; ***, *P* < 0.001; two-way ANOVA, TUKEY test)

The Mediterranean climate in the study area was characterised by low temperatures during the first months of growth and high temperatures during the later stages of grain filling (Fig. 1c). Total rainfall was low during 2018/2019 crop season (127.5 mm), so irrigation (which implied an additional 171.1 mm) was an essential factor in the differences between water regimes in durum wheat growth and production. Differences due to water input at the agronomic, grain quality and physiological level were analysed using multivariate (PCA, Supplementary Fig. S1) and univariate (Figs. 2, 3) analyses. Dimension 1 of the PCA explained 41.1% of the variability and was clearly associated with the effect of water input (Supplementary Fig. S1). Dimension 2 explained 13.4% of the variability and was partly associated with the genotypic variability, which was more evident under irrigated conditions.

Rainfed conditions resulted in an overall grain yield reduction of 64% for the five varieties studied, with non-significant differences among varieties (Fig. 2a). Likewise, rainfed conditions also reduced biomass by 52% and other agronomic traits such as harvest index, ears m^−2^, ears plant^−1^, grains ear^−1^, GCY, and GNY (Supplementary Table S1 and Fig. S1). While irrigation increased grain δ^15^N, it decreased δ^13^C (Fig. 2b and Supplementary Fig. S1). The RWC in flag leaves was progressively reduced under rainfed conditions from ear emergence to mid-grain filling, with some significant differences between varieties (Fig. 2c and Supplementary Table S1).

Regarding grain quality traits, the results were more heterogeneous and showed that they were influenced by both the environment and the variety (Supplementary Table S1 and Fig. S1). Under rainfed conditions, protein content, vitreousness, SDS sedimentation, and wet gluten increased independently of the variety. On the other hand, thousand kernels weight (TKW) decreased in rainfed conditions, while the genotype × environment interaction was significant for the b* and GI parameters. Concerning the nutritional quality of the grain, significant changes were observed for 9 out of the 11 micro- and macro-nutrients as response to water regime, environment or the interaction effects (Supplementary Table S1 and Fig. S1). Among the most significant changes, N and S concentrations in grain increased under rainfed conditions compared to irrigation, whereas Mg, P, Cu and Mn decreased.

At canopy level, rainfed conditions led to lower NDVI values than under irrigation, and these were significantly different at the stage of flag leaf sheath extension (Zadoks 40) onwards (Fig. 3a and Supplementary Table S1). While significant differences between varieties were observed at the intermediate stages (Zadoks 35–55), mainly under rainfed conditions, these disappeared at the last sampled stage (anthesis). Regarding leaf pigments, Chl content was reduced in rainfed conditions relative to irrigation, being more evident at later stages and in Kiko Nick compared to the other varieties (Fig. 3b and Supplementary Table S1). The same pattern was observed for nitrogen balanced index (NBI). Flavonoids content increased slightly at anthesis under rainfed conditions and, together with anthocyanins, more intensely at mid-grain filling, particularly in Kiko Nick.

Although our study has little variability in grain yield between varieties for each water regime, as well as a limited number of observations, we have found some significant correlations between grain yield and other agronomic and grain quality traits (Supplementary Fig. S2). Under irrigated conditions, grain yield correlated positively with the number of grains per ear, while it was negatively associated with vitreousness and wet gluten. Under rainfed conditions, grain yield correlated positively with biomass, harvest index, test weight and TKW, and negatively with grain protein content and b*.

### Differences in the concentration of primary metabolites between the photosynthetic organs and effect of water regime on C metabolites

**Fig. 4:**
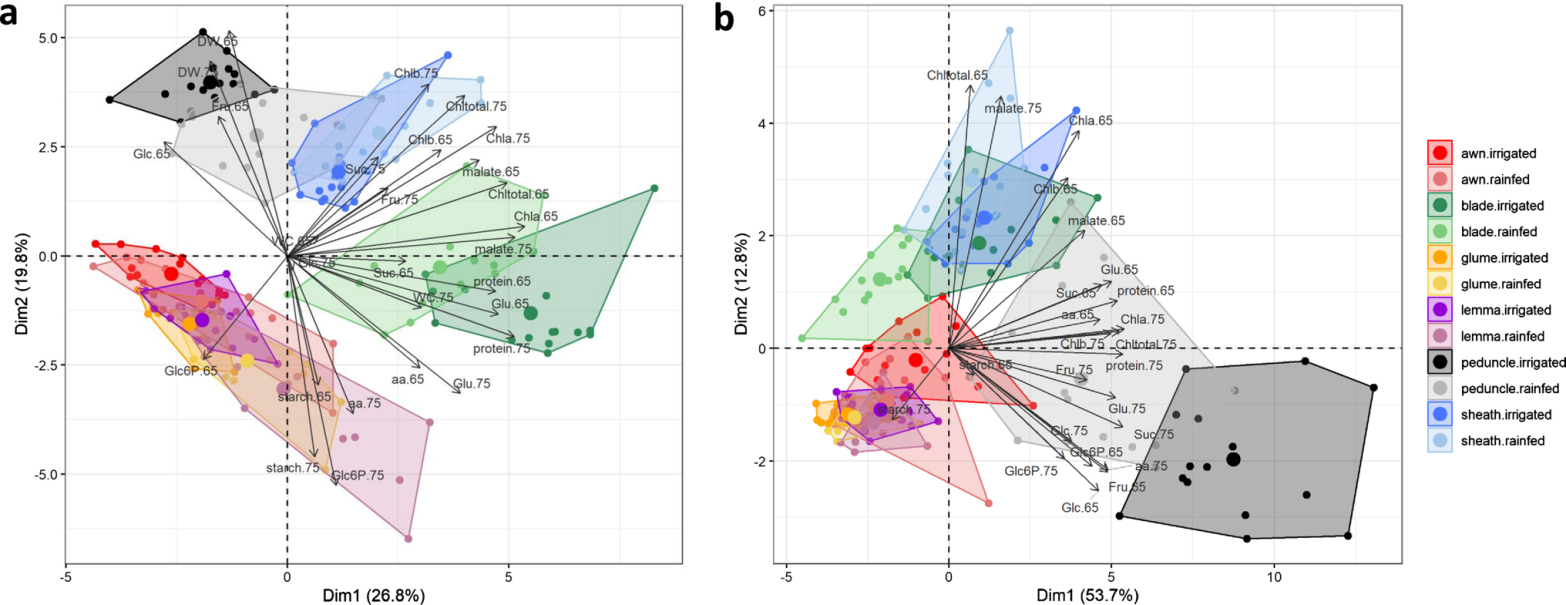
**a** Principal component analysis (PCA) of dry weight (DW), water content (WC), and C and N metabolites expressed as concentrations. **b** PCA of C and N metabolites expressed as total content per organ. The C metabolites include glucose (Glc), glucose-6-phosphate (Glc6P), fructose (Fru), sucrose (Suc), starch, and malate, while the N metabolites include glutamate (Glu), total amino acids (aa), proteins, and chlorophylls a (Chla), b (Chlb) and total (Chltotal). Six photosynthetic organs (leaf blades and sheaths, peduncles, awns, glumes and lemmas) of five durum wheat varieties (MEX, Mexa; EUR, Euroduro; DRI, Don Ricardo; KNI, Kiko Nick; HAR, Haristide) under rainfed and irrigated conditions were considered. The measurements were carried out at anthesis (Zadoks 65) and mid-grain filling (Zadoks 75)

**Fig. 5:**
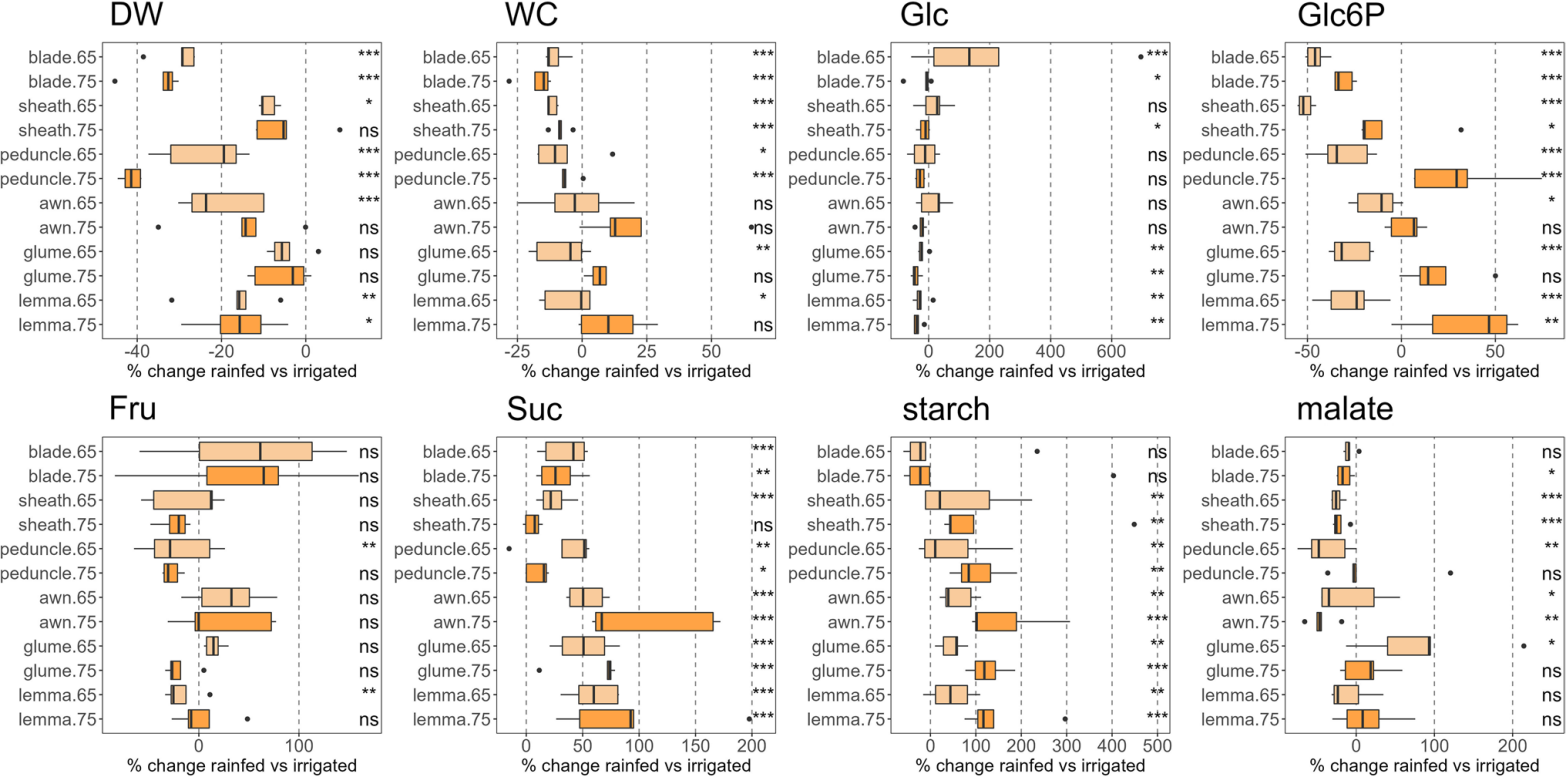
Effect of water regime on dry weight (DW), water content (WC), and C metabolites [glucose (Glc), glucose-6-phosphate (Glc6P), fructose (Fru), sucrose (Suc), starch, and malate] in six photosynthetic organs during anthesis (Zadoks 65) and mid-grain filling (Zadoks 75). The boxplots were built per organ using the data from five durum wheat varieties using the percentage of change in rainfed compared to irrigated conditions for every variety. Symbols on the right of each figure indicate the significance of the water regime effect according to the two-way ANOVA in Supplementary Table S1 (ns, not significant; *, *P* < 0.05; **, *P* < 0.01; ***, *P* < 0.001; two-way ANOVA, TUKEY test)

**Fig. 6:**
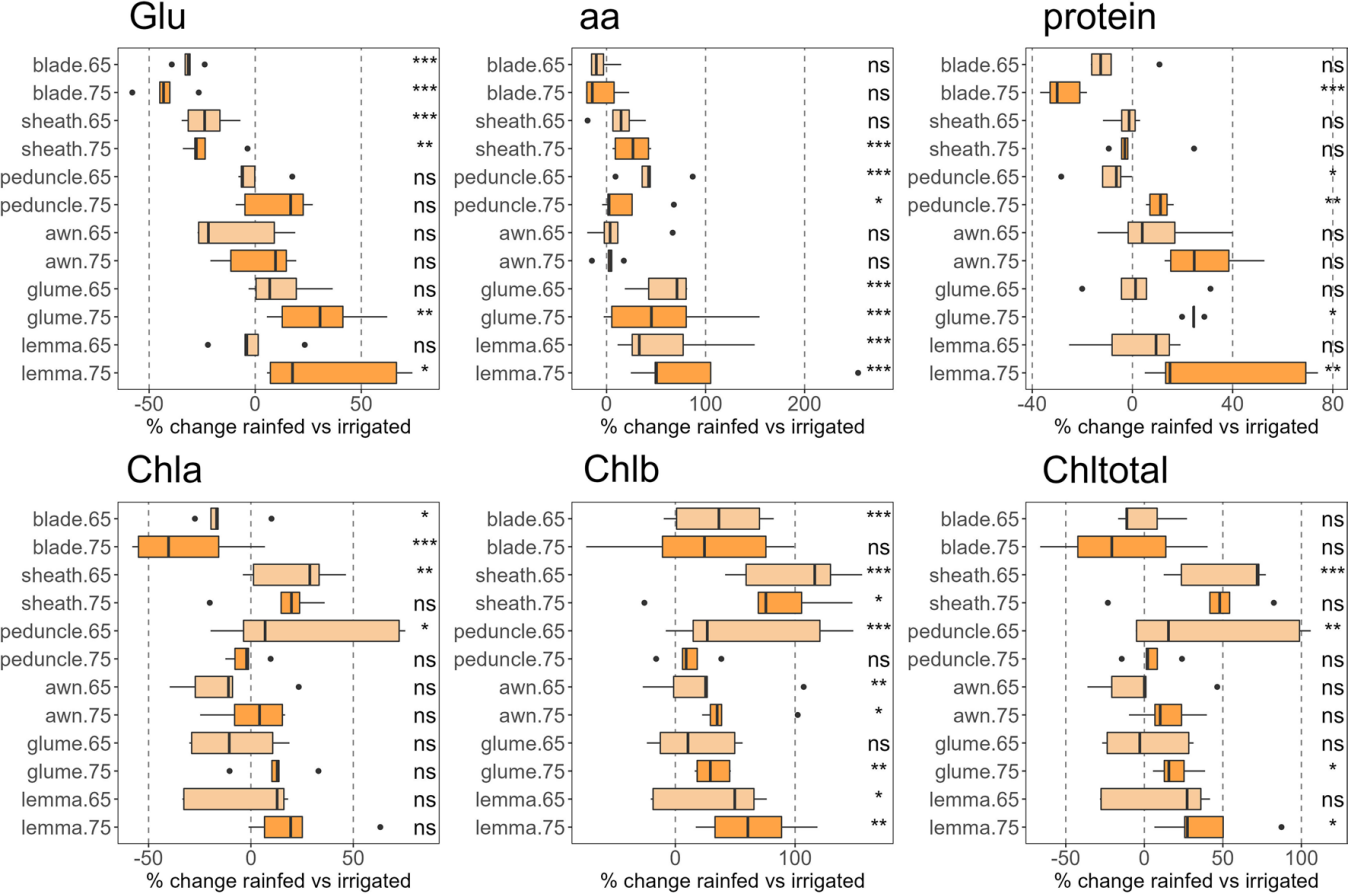
Effect of water regime on N metabolites [glutamate (Glu), total amino acids (aa), proteins, and chlorophylls a (Chla), b (Chlb) and total (Chltotal)] in six photosynthetic organs during anthesis (Zadoks 65) and mid-grain filling (Zadoks 75). The boxplots were built per organ using the data from five durum wheat varieties by using the percentage of change in rainfed compared to irrigated conditions for every variety. Symbols on the right of each figure indicate the significance of the water regime effect according to the two-way ANOVA in Supplementary Table S1 (ns, not significant; *, *P* < 0.05; **, *P* < 0.01; ***, *P* < 0.001; two-way ANOVA, TUKEY test)

Once the effects of water stress and genotypic variability were characterised at the physiological and agronomic levels, we focussed on the metabolic changes between photosynthetic organs at anthesis and mid-grain filling (Fig. 1d, e). We performed a multivariate analysis of the results for DW, WC and diverse C and N metabolites in the blades and sheaths from the flag leaves, peduncles, awns, glumes and lemmas (Fig. 4a). The metabolic data were expressed in concentration per DW, avoiding the use of FW, which could be greatly influenced by organ water content. PCA dimension 1 explained 26.8% of the variability, while dimension 2 explained 19.8% (Fig. 4a). The organ was the main factor affecting the metabolic traits. Ear organs (awns, glumes and lemmas) were grouped together regardless of the water regime, showing the highest concentrations of Glc6P and starch in general. On the other hand, the peduncle and the leaf sheath were relatively close in the PCA. The peduncle had the highest DW, as well as Glc and Fru concentrations among the different organs at anthesis, and its metabolism was significantly altered by water stress. The leaf sheath exhibited little variability in response to the water regime, with high concentrations of Chlb at anthesis and mid-grain filling, and Suc at the latter stage. The blade was separated from the other organs and showed a tendency towards the highest concentrations of some metabolites, mainly Chl and Suc at anthesis and proteins and malate at both growth stages. However, blades showed the greatest effect of water regime according to the multivariate and univariate analyses (Figs. 4a, 5, 6).

A more detailed analysis of the response of photosynthetic organs to water stress showed that FW and DW in the different organs were significantly reduced by rainfed conditions, with some differences between varieties (Fig. 5 and Supplementary Table S1). However, to study the differences between photosynthetic organs in durum wheat, we grouped the five varieties and observed that DW was strongly reduced in blades (30–35%) and peduncles (24–41%), followed by awns (15–20%), lemmas (16–17%), sheaths (5–9%) and glumes (5–6%) under rainfed conditions (Fig. 5). By contrast, WC decreased significantly in blades, sheaths and peduncles, while in the three ear organs, this reduction was smaller at anthesis or even increased by 6–22% at mid-grain filling. In leaf blades, the Glc concentration increased dramatically in rainfed compared to irrigated conditions at anthesis (up to 204%), but decreased at grain filling (Fig. 5). Irrespective of the growth stage, Glc decreased in the bracts (21–42%). Glc6P significantly decreased in all organs at anthesis under rainfed conditions. However, this reduction was only observed in the foliar organs (blades and sheaths) at mid-grain filling and, conversely, increased in the peduncles, glumes and lemmas. Fru decreased in the peduncles and lemmas at anthesis under rainfed conditions, while the increase in blades and awns did not reach statistical significance (Supplementary Table S1). Rainfed conditions significantly increased the Suc concentration in almost all organs regardless of the growth stage. This increase was organ-specific, being 29–35% in blades, 6–25% in sheaths, and 11–35% in peduncles, and higher in ear organs (53–105%, 51–62% and 60–92% for awns, glumes and lemmas, respectively). Similarly, starch concentrations increased markedly in all organs under rainfed conditions, except in blades, with the highest increase at mid-grain filling in peduncles and the three ear organs (reaching 158% in awns). Malate significantly decreased under rainfed conditions in blades and awns at mid-grain filling, peduncles at anthesis, and sheaths at both stages. However, it was not affected in ear bracts, with even a substantial increase in glumes at anthesis (86%).

### Effect of water regime on the concentration of N metabolites in the photosynthetic organs

The effect of water stress on Glu concentration was highly dependent on the organ (Fig. 6). It decreased at anthesis and mid-grain filling in blades (32–43%) and sheaths (23–24%) under rainfed conditions, but was not altered in peduncles and awns and increased in glumes (30%) and lemmas (34%) at mid-grain filling. The concentration of amino acids significantly increased under rainfed conditions in sheaths at mid-grain filling (26%) and at both stages in peduncles (19–44%), glumes (57–59%) and lemmas (59–97%, Fig. 6). The Chla concentration decreased in blades at both stages under rainfed conditions and increased in sheaths and peduncles at anthesis, while Chlb tended to increase in all organs. Overall, Chltotal levels increased in the sheaths and peduncles at anthesis and in the ear bracts at mid-grain filling.

### Total organ content of primary metabolites reveals significant organ-specific differences in C-rich metabolites

The levels of C and N metabolites were also expressed as total content per organ and analysed with a PCA and two-way ANOVA for the organ × water regime interaction. PCA dimension 1 explained 53.7% of the variability, while dimension 2 explained 12.8% (Fig. 4b). A large part of the metabolites tended to accumulate in peduncles. Only starch content was higher in the ear organs (mainly in awns and lemmas) and Chltotal at anthesis and malate at both stages in blades and sheaths. The differences between the photosynthetic organs were narrowed when expressing metabolites by total content as opposed to concentration values. The awns, glumes and lemmas were again located close to each other, with a shorter distance to the blade in this case (Fig. 4b). Interestingly, the sheath overlapped with the blade under irrigated conditions, whereas the two organs differed more markedly under rainfed conditions. While the effect of the water regime was less pronounced on sheaths and ear bracts, it was more marked on blades, peduncles and awns.

**Fig. 7:**
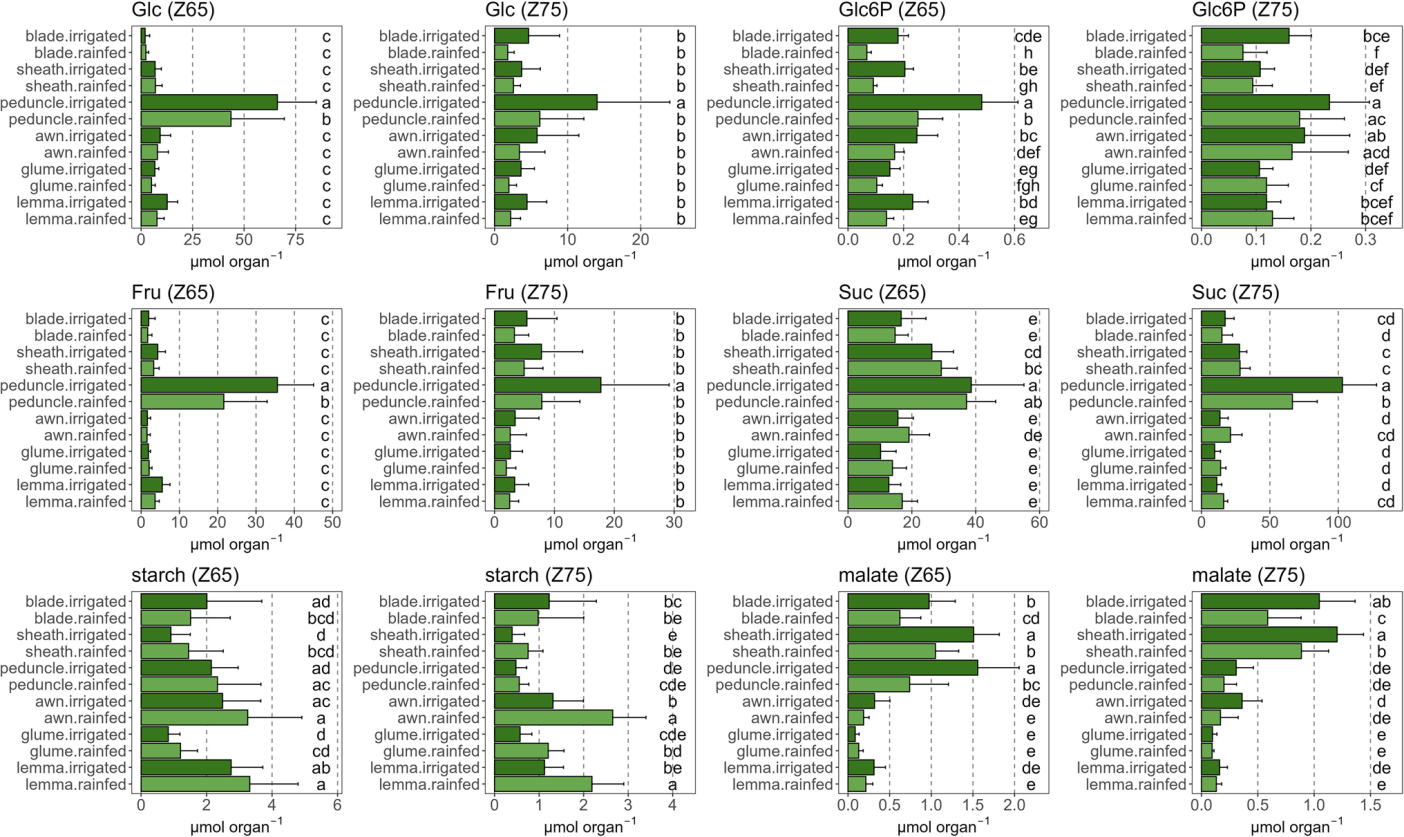
Differences in the content of metabolites related to C metabolism [glucose (Glc), glucose-6-phosphate (Glc6P), fructose (Fru), sucrose (Suc), starch, and malate] per organ and water regime. The analyses were performed in six photosynthetic organs during anthesis (Z65) and mid-grain filling (Z75). For each comparison of means, letters are significantly different as detailed in Supplementary Table S2 (*P* < 0.05; two-way ANOVA, TUKEY test)

The univariate analyses revealed that the total organ contents of Glc and Fru were quantitatively higher in peduncles compared to the other organs (Fig. 7). While Glc6P had a similar trend to Glc, the differences between organs were attenuated with an apparent effect of water stress on all organs at anthesis. This negative impact of water stress on Glc6P disappeared at mid-grain filling mainly in the three ear organs. Suc content was again higher in peduncles, mainly at mid-grain filling, and was negatively affected by rainfed conditions (Fig. 7). There was a clear tendency for the Suc pool to increase in all three organs of the ear under rainfed conditions, but this did not reach statistical significance. Starch content was slightly higher in awns and lemmas, while rainfed conditions clearly increased its levels in sheaths, awns, lemmas, and glumes compared to irrigated conditions. The malate content was markedly higher in blades and sheaths at both stages and in peduncles at anthesis, and was significantly and negatively affected by rainfed conditions, but not in the case of the ear organs.

Changes in total metabolite content per organ between anthesis and mid-grain filling showed that under irrigation metabolites such as Glc in blades, and malate in awns and glumes increased significantly at later stages (Supplementary Fig. S3). Moreover, at both water regimes Fru in blades, sheaths and awns, and Suc in the peduncle also increased at mid-grain filling. Glc6P and starch tended to decrease at mid-grain filling under irrigation, with a smaller decrease or even an increase under rainfed conditions. Finally, to highlight the total pool of sugars available in the plant, we added up Glc, Glc6P, Fru, Suc and malate, and observed that quantitatively it is much higher in structures with transport and storage functions (sheaths + peduncles), followed by the ears (awns + glumes + lemmas), far from the leaf blades at anthesis and mid-grain filling regardless of water regime (cf Fig. 9).

### Contrasting total organ content of N metabolites between organs, with reduced effect of water stress on ear organs

**Fig. 8:**
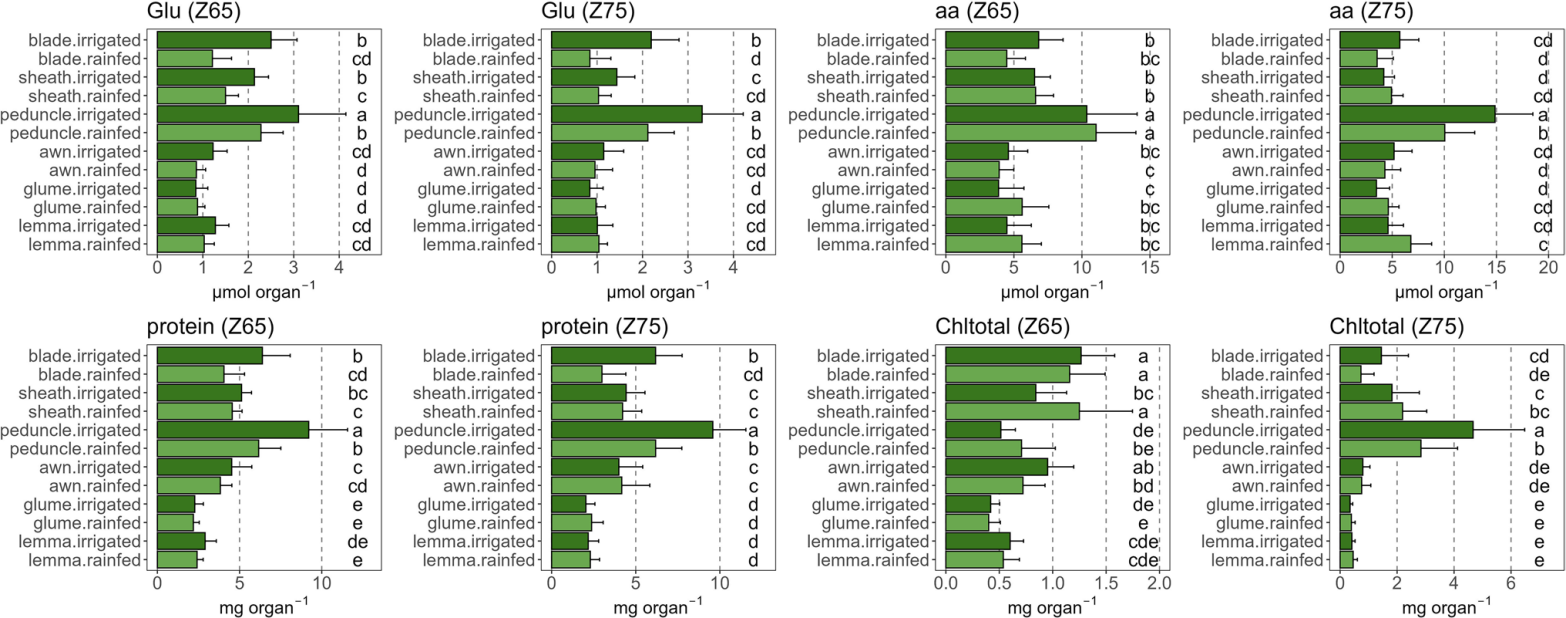
Differences in the content of metabolites related to N metabolism [glutamate (Glu), total amino acids (aa), proteins, and total chlorophylls (Chltotal)] per organ and water regime. The analyses were performed in six photosynthetic organs during anthesis (Z65) and mid-grain filling (Z75). For each comparison of means, letters are significantly different as detailed in Supplementary Table S2 (*P* < 0.05; two-way ANOVA, TUKEY test)

The Glu content was particularly higher in blades, sheaths and peduncles at anthesis, compared to the other organs under irrigated conditions (Fig. 8). Interestingly, its levels decreased in rainfed compared to irrigated conditions in these three organs, but not in awns, glumes and lemmas. The free amino acids content was higher in peduncles and similar in the rest of the photosynthetic organs, with only a slight decrease in blades at both stages and the peduncle at mid-grain filling under rainfed conditions, as well as a small increase in ear bracts. Soluble protein levels were highest in peduncles and lowest in bracts. Furthermore, water stress strongly decreased the soluble protein levels in peduncles and blades, with no significant changes in the other organs. Chltotal content was highest in blades, sheaths and awns at anthesis and in peduncles at mid-grain filling. Water stress increased Chltotal levels in sheaths at anthesis, while it reduced them in peduncles and blades at mid-grain filling.

**Fig. 9:**
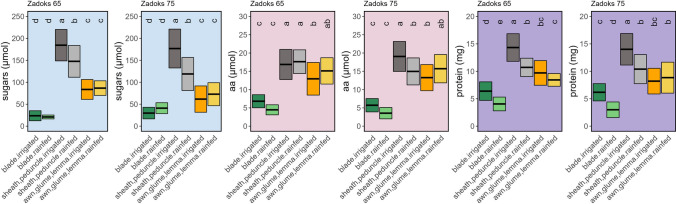
Total content of sugars, amino acids (aa), and proteins per organ (or group of organs) and water regime during anthesis (Zadoks 65) and mid-grain filling (Zadoks 75). The comparison of the flag leaf blade with the organs with a function more related to transport and storage (sheath + peduncle) and the ear organs (awn + glume + lemma) has been highlighted. Sugar content is the sum of glucose, glucose-6-phosphate, fructose, sucrose, starch, and malate. Each box represents the average ± standard deviation of five field-grown durum wheat varieties. For each comparison of means, letters are significantly different (*P* < 0.05; two-way ANOVA, TUKEY test)

Metabolic changes between anthesis and mid-grain filling (Supplementary Fig. S3) showed that in general the levels of N-rich metabolites (Glu, amino acids and proteins) decreased in the photosynthetic organs, but this effect was smaller or even reversed in ear organs at both water regimes or in peduncles only at irrigated conditions. Chltotal also increased significantly in the peduncles. To highlight the N distribution in the plant, using amino acids and proteins as representative outputs of N metabolism, we grouped the organs with transport/storage function (sheaths + peduncles) and the ears (awns + glumes + lemmas; Fig. 9). We observed that their N pool is much higher than that of leaf blades at any late stage. Interestingly, protein and amino acid levels in ears remained unaltered or even tended to be higher under water stress compared to irrigated conditions, which was not the case in the other organs.

## Discussion

In the present study, five durum wheat varieties were grown under field conditions in a region (Castile and León, Spain) with a marked continental Mediterranean climate. The climate is classified as Csb (temperate, dry and warm summer) according to the Köppen–Geiger classification (Beck et al. 2018). It is characterised by low rainfall and high temperatures, especially at the grain filling stages (T_max_; Fig. 1c), when temperatures greatly exceeded the optimum for photosynthesis (Farooq et al. 2011). In fact, the study showed lower rainfall values compared to previous years (Spanish State Meteorological Agency; http://www.aemet.es/), which led to characteristic and severe water stress effects (Figs. 2–3) that will become increasingly common in the study region in the coming years (Beck et al. 2018).

### Water stress reduced crop yield and biomass, and altered grain quality in five field-grown durum wheat varieties

In the genotype × water regime interaction, water regime was the main factor contributing to the variability in the agronomic and physiological traits (Supplementary Table S1 and Fig. S1). Although some of these traits showed significance for genotypic variability, grain yield and biomass at harvest did not vary among the five varieties for each water level, which allows us to generalise the response of durum wheat to water stress and the factors affecting grain yield in our study (Figs. 2, 3a). Overall, water stress reduced biomass by decreasing ears per area and per plant, as well as grains per ear, factors that led to a lower harvest index and grain yield. Indeed, these parameters have been argued as being the most important in determining yield under contrasting water inputs (De Santis et al. 2021). The correlation matrix for each water regime highlighted that grain yield was mainly determined by a higher number of grains per ear under irrigated conditions and by a higher biomass and harvest index under drought conditions (Supplementary Fig. S2). Furthermore, yield increases have an important impact on grain quality depending on water conditions.

The δ^13^C indicates the biochemical discrimination that takes place during CO_2_ assimilation when analysed in plant dry matter, which is intimately related to water use efficiency (WUE) during the crop cycle (Araus et al. 2022). Our results evidenced that a severe water stress took place in all the varieties leading to a loss of leaf turgor (RWC), that affected the metabolic capacities, even if WUE improved under rainfed conditions as a water-saving strategy (Bowne et al. 2012; Sanchez-Bragado et al. 2014a; Chairi et al. 2018; Fig. 2). The changes in spectral indices and GNY suggested that water stress led to an inhibition of N assimilation at the canopy and leaf levels (Supplementary Table S1). Additionally, changes in δ^15^N may reflect the downregulation of N uptake, assimilation and/or translocation taking place under rainfed conditions, making δ^15^N a natural tracer of N fluxes (Cui et al. 2020; Araus et al. 2022). Yield decline under water stress was associated with smaller kernel size, which is in agreement with De Santis et al. (2021), but grain N and protein concentrations were higher, probably due to a concentration mechanism (Xu and Yu 2006; Hoyle et al. 2020). Moreover, water stress led to positive modifications in grain quality for the industry (e.g. higher S content, SDSS, vitreousness and wet gluten), but also other negative ones, such as an impoverishment of Mg, P, Cu and Mn (Supplementary Table S1).

Canopy NDVI results indicated that senescence began prematurely due to water limitation (Moraga et al. 2022). This shortening of the life cycle (shorter grain filling periods) and/or decreased ability of source organs to supply nutrients may have been associated with the lower yields under rainfed conditions (De Santis et al. 2021). The leaf spectral index for Chl content was a good proxy for water stress symptoms, but also for genotypic variability. This index, as well as the nitrogen balanced index (NBI), suggested a decrease in foliar N content under water stress, while the increase in the flavonoids and anthocyanins (Fig. 3) may indicate a response to counteract the oxidative damage that typically appears in drought (Cerovic et al. 2012; Landi et al. 2015; Agati et al. 2020). Overall, the different agronomic and physiological traits provided evidence for the existence of a water limitation in the five durum wheat varieties and that it was severe for most traits analysed. Water stress promoted WUE, differentially modified grain quality traits, and led to a significant decrease in biomass, productive tillers, grains per ear, and C and N assimilation that negatively impacted grain yield.

### Organ-specific metabolic responses to water stress: blades and peduncles as the most sensitive, ear organs as the most stable

Metabolic variation was analysed between organs, growth conditions and stages, combining varieties together as a pool due to the low genotypic variability found for grain yield in each water regime. Metabolite levels varied more between organs than with water regime, as previously reported in durum wheat (Vergara-Diaz et al. 2020; Martínez-Peña et al. 2022) and rice (Lawas et al. 2019; Yang et al. 2021). The peduncle had the highest DW of the six photosynthetic organs (Fig. 4a), which directly impacted on the total metabolite content per organ (see next section). The blade possessed the highest concentration of several primary metabolites (Supplementary Table S1), which reflects its key function of acting as source organ to provide nutrients to other parts of the plant (Sun et al. 2013; Vicente et al. 2018a).

The metabolic status of peduncles and sheaths was similar (Fig. 4a, Supplementary Table S2), as these organs seem to exhibit similar functions that are predominantly related to nutrient transport and storage for later stages of grain filling, as previously reported in durum wheat (Cimini et al. 2015; Martínez-Peña et al. 2022) and barley (Torralbo et al. 2019). We previously found in field-grown durum wheat that sugar content (Suc and starch) and N metabolism enzyme activities (glutamine synthetase and ferredoxin-dependent glutamate synthase) in peduncles correlated negatively with yield (Martínez-Peña et al. 2022). Conversely, the activation state of Rubisco during late stages was positively associated with yield, which may suggest some role in providing newly fixed CO_2_ to the grains (Kong et al. 2010; Rivera-Amado et al. 2020). Therefore, whether a larger peduncle for storing leaf photoassimilates or a higher photosynthetic and N metabolism capacity in the peduncles plays a significant role in yield requires future research.

The peduncles and the blades were the most susceptible organs to water stress, with little effect observed in sheaths and awns as inferred by water stress-related reductions in C and N metabolites and DW (Figs. 4a, 5, 6, and Supplementary Table S2). The tendency towards higher levels of hexose sugars in blades compared to other organs in response to water stress, or progressing in phenology (Fig. 5, Supplementary Fig. S3), was not associated with starch or Suc breakdown to act as C building blocks and energy sources (Cimini et al. 2015; Živanović et al. 2020). Therefore, and given the magnitude of the change, we suggest that their increase was linked to a role as osmoprotectants (Živanović et al. 2020). Suc is the main sugar transported from sources to sinks in most plants (Stein and Granot 2018), and it can also act as an osmoprotectant under stress (Živanović et al. 2020). The overall increase in Suc in all organs under water stress could indicate an osmoprotective function, associated with the significant decrease in RWC and increase in flavonoids and anthocyanins observed in flag leaf blades. Interestingly, the increase in Suc content was higher in awns, glumes and lemmas compared to the other organs under water stress. It could be associated, at least in part, with a greater stability of the photosynthetic apparatus in these organs, as they were the only ones where WC did not decrease. Nevertheless, we cannot fully discern whether Suc increase was linked to photosynthesis or to its osmoprotective function. Furthermore, among the sugars analysed, starch is the only one with a function mainly associated with photosynthesis, as it is a reserve carbohydrate that forms granules, so it cannot participate in cell protection. Thus, the markedly increase in starch in ears and peduncles under water stress (Fig. 5) may indicate either an improved C status, particularly at late stages, or a poor translocation of sugars to the grain. However, the increase in N-rich compounds (Glu, aa, and proteins; Fig. 6) in the ear organs would point more to the first option, where an improved provision of C skeletons would favour the synthesis of these compounds. Indeed, our data support the idea that Glc decreased in these non-foliar organs because it was the substrate for starch synthesis, as we subsequently confirmed with the strong increase in one intermediate of this pathway, Glc6P. This better performance of ears under stress and key contribution to grain filling has been previously supported using analyses of δ^13^C in its natural abundance (Sanchez-Bragado et al. 2014a, 2020). This could be reinforced by the aforementioned increase in starch synthesis in these organs, which is only activated when the C status is optimal in the cell (Kolbe et al. 2005). Malate content, which is another transient C reserve also involved in respiration (Finkemeier and Sweetlove 2009; Barros et al. 2020), was not affected in the ear bracts by water stress or it was even increased at later stages (Fig. 5, Supplementary Fig. S3), supporting their better C status.

The ear organs also showed better N status than the other organs, particularly at mid-grain filling (Figs. 4a, 6). In our earlier study in durum wheat (Martínez-Peña et al. 2022), we reported that the activities of key enzymes in C and N metabolism, e.g. Rubisco, phosphoenolpyruvate carboxylase, glutamate synthase, and glutamate dehydrogenase, had a greater correlation with yield than the same activities in other organs such as the blades. Similarly, Shokat et al. (2020) showed that the activities of enzymes related to antioxidant capacity and carbohydrate metabolism in the whole ears correlated with bread wheat yield. This is interesting given our knowledge of the lower content and enzyme activities of photosynthetic proteins in these organs (Martínez-Peña et al. 2022), which leads to a higher C/N ratio compared to blades (Vicente et al. 2018b), and this is due to the higher N-cost of these proteins. By tracking the δ^15^N of dry matter in the durum wheat photosynthetic organs, Sanchez-Bragado et al. (2017) suggested that a substantial portion of the N content in the grains came from the ears, comparable to the provision of N from the vegetative parts of the plants, and this was even more relevant as water and N stresses progressed during crop cycle. Our data support this study, showing that the pool of Glu, free amino acids, soluble proteins and Chl were not affected in ears by water stress in the same way as in the blades (Fig. 6). This was also observed with a metabolomic approach in durum wheat ear bracts vs. blades (Vergara-Diaz et al. 2020). The higher levels of N metabolites in ears under water stress was in line with our previous study where the expression of key N metabolism genes was up-regulated in durum wheat ears during early grain filling, while these genes were strongly inhibited in leaf blades (Vicente et al. 2018b). Overall, at the concentration level, C and N metabolites show organ-specific levels (Figs. 5, 6), with maintenance or enhancement in ears in response to stress, mainly at late stages. The stress resilience of these organs may be of interest for improving grain yield and quality under unfavourable growth conditions.

### The total metabolite content per organ shows that the peduncles are the major reservoir of carbon and nitrogen during grain filling and that the ear organs may play an important role under water stress conditions

Leaf photoassimilates and sugars accumulated in the stems have traditionally been considered the main sources of nutrients for grain filling in cereals such as wheat. However, there is growing evidence that the photosynthetic and N assimilation roles of non-foliar organs are more relevant under limiting conditions (Sanchez-Bragado et al. 2020; Tambussi et al. 2021). The peduncle’s relevance in grain filling is clear because we demonstrated that this plant part possesses the highest pool of primary metabolites during grain filling (Figs. 7, 8, 9). Peduncles accumulated remarkably high levels of Fru compared to the rest of the organs, as well as Glc and Suc, which is directly related to the synthesis of fructans. These sugars are one of the main sources of C to nourish the growing grains once plant photosynthesis (in leaves and ears) ceases in the late stages of grain filling (Takahashi et al. 2001; Cimini et al. 2015; Martínez-Peña et al. 2022). Under rainfed conditions, the decreased Fru levels (and likely fructan levels; Fig. 7) in the peduncle could explain part of the decline in yield observed. Although the high levels of sugars in this organ are largely due to C translocation from the source organs below the peduncle, its photosynthetic rate, associated with Rubisco and phosphoenolpyruvate carboxylase activities alongside a high stomatal density, is still relevant at late stages of grain filling (Kong et al. 2010; Martínez-Peña et al. 2022). In addition, the peduncle was also a significant source of N compounds (Glu, amino acids and proteins; Fig. 8), which may have a metabolic rather than a storage or structural origin (Barraclough et al. 2014), although they were also significantly reduced by water stress. In line with its possible photosynthetic role, we observed a large increase in chlorophyll content in the peduncle from anthesis to mid-grain filling regardless of water regime (Supplementary Fig. S3). Furthermore, most of the metabolites analysed did decrease under rainfed conditions compared to irrigation in both peduncles and blades, highlighting the susceptibility of these organs to water stress.

From a quantitative point of view, it is surprising that there were no decreases observed in most of the metabolites analysed in the three organs of the ear under water stress (Figs. 7, 8). The stability of their sugar, amino acid and protein levels and their high total content relative to the leaf blades (Fig. 9) reflects its stress tolerance and relevance for supplying C and N to the developing grain at late stages. Vergara-Diaz et al. (2020) also identified a better N status and levels of organic acids under water limitation in durum wheat ears relative to blades, but the study was restricted to ear bracts and the values were only expressed as concentrations. Intriguingly, malate content was unique in remaining unaltered in ear organs under rainfed conditions. Nevertheless, its content was lower than in the non-ear organs and further research is required to discover whether ear organs preferably use other C intermediates (Finkemeier and Sweetlove 2009; Barros et al. 2020). In contrast, the ears redirected more C towards starch than other organs, as discussed above.

In summary, the absolute levels of soluble C and N compounds that are available during grain filling quantitatively demonstrate that all green photosynthetic organs contribute to grain filling to a greater or lesser extent. Supported by previous isotopic, transcriptomic, metabolomic, and enzymatic studies, most of them reviewed in Hu et al. (2018), Sanchez-Bragado et al. (2020) and Tambussi et al. (2021), we conclude that peduncles and ears have important roles in providing C and N to the grain, the former due to their high metabolic content and the latter for their tolerance to stress, especially when the flag leaves cease their photosynthetic activity during late growth stages and under water stress. The biomass, exposure to light, late senescence and proximity to the grains of non-foliar photosynthetic organs support their role in the synthesis of photoassimilates that may be reallocated to the grains.

## Conclusions

In the present study, the analysis of key C and N metabolites in the different green organs, both at the concentration level and in absolute values per organ, allowed us to predict their relevance during grain filling and susceptibility to water stress. The ear organs have a greater stability in response to contrasting water regimes, while the blades and peduncles were more susceptible. Ear organs, especially bracts, seem to maintain or even increase their pool of metabolites in response to water stress, showing better water, C and N status and highlighting a key role for grain filling under stress conditions. The peduncle proved to be the organ with the largest pool of C and N during grain filling, mainly due to its higher dry weight and its ability to store nutrients. Yield reductions under rainfed conditions were associated with an inhibition of C and N assimilation and their translocation to grains (Xu and Yu 2006; Fresneau et al. 2007; Farooq et al. 2014). Then, the decrease in yield was the consequence of fewer shoots being capable of producing ears and fewer grains per shoot, accompanied by the reduced metabolic capacity of blades and peduncles under these conditions. We observed that there was genotypic variability for the metabolic status in the different organs, indicating that there is a large window for crop improvement under stress conditions given the possibility of using the existing natural variability in durum wheat, but also extrapolated to other C_3_ cereals. Many efforts in recent decades have focussed on improving the photosynthetic capacity of leaves (Araus et al. 2021), but it is possible that redirecting our efforts to other regulatory systems around source-sink coordination at the whole-plant level in dynamic environment and time scales may contribute to further progress (Paul 2021; Reynolds et al. 2022).

### *Author contribution statement*

RV, NA and JLA conceived and designed the research. RMP, NA, AS, MH, RM, and MTNT performed the experiments and analytical measurements. RMP, RV and OVD analysed the data. RV wrote the manuscript, while OVD, JLA, RM, AS and RMP reviewed and edit it. All authors read and approved the manuscript.

## Supplementary Information

Below is the link to the electronic supplementary material.

Supplementary file1 (DOCX 4240 kb)

## Data Availability

All data generated or analysed during this study are included in this published article [and its supplementary information files]. Raw data, including biological replicates, are available from the corresponding author on reasonable request.

## Abbreviations

DW: Dry weight
FW: Fresh weight
Fru: Fructose
GCY: Grain carbon yield
GNY: Grain nitrogen yield
Glc: Glucose
Glc6P: Glucose-6-phosphate
Glu: Glutamate
NDVI: Normalised difference vegetation index
PCA: Principal component analysis
Suc: Sucrose
WC: Water content
WUE: Water use efficiency
