# Supplementary material for: Analysis of durum wheat photosynthetic organs during grain filling reveals the ear as a water stress-tolerant organ and the peduncle as the largest pool of primary metabolites
Source: Planta. 2023 Mar 14;257(4):81. doi: 10.1007/s00425-023-04115-1 (PMC10014764; doi:10.1007/s00425-023-04115-1)

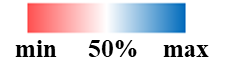
**Supplementary Table S1**. Effect of genotypic variability (G), water regime (W) and their interaction (G×W) on agronomic traits, C and N isotope composition, grain quality traits, grain mineral content, spectral vegetation indices, leaf relative water content, and organ-specific fresh and dry weights, water content, and C [glucose (Glc), glucose-6-phosphate (Glc6P), fructose (Fru), sucrose (Suc), starch, and malate] and N [glutamate (Glu), total amino acids (aa), proteins (Prot), chlorophylls a (Chla), b (Chlb) and total (Chltotal)] metabolites. The numbers in the traits represent the Zadoks scale when they were measured: tillering, stem elongation, flag leaf sheath extension, boot swollen, ear emergence, anthesis, and mid-grain filling (Zadoks 25, 35, 40, 45, 55, 65 and 75, respectively). The organ-specific traits were expressed as concentration in dry weight (DW) and as total organ content. The means in each row with different letters are statistically different (*P*<0.05; two-way ANOVA, TUKEY test; yellow colour indicates the significance of a factor). The colour scale in the means shows the minimum (red) and maximum (blue) values per trait. The rest of the abbreviations are described throughout the text. MEX, Mexa; EUR, Euroduro; DRI, Don Ricardo; KNI, Kiko Nick; HAR, Haristide; b, blade; s, sheath; p, peduncle; a, awn; g, glume; l, lemma; e, the whole ear.

| **Variable** | **Units** | **Irrigated** | | | | | **Rainfed** | | | | | ***P*-value** | | |
| --- | --- | --- | --- | --- | --- | --- | --- | --- | --- | --- | --- | --- | --- | --- |
|  |  | **MEX** | **EUR** | **DRI** | **KNI** | **HAR** | **MEX** | **EUR** | **DRI** | **KNI** | **HAR** | **G** | **W** | **G×W** |
| **GY** | kg ha^-1^ | 7220 ± 291^a^ | 7270 ± 852^a^ | 7050 ± 979^a^ | 7550 ± 1050^a^ | 7070 ± 818^a^ | 2520 ± 222^b^ | 2390 ± 291^b^ | 3380 ± 241^b^ | 2410 ± 279^b^ | 2180 ± 105^b^ | 0.909 | 0.000 | 0.784 |
| **GNY** | kg ha^-1^ | 169 ± 31.8^ab^ | 197 ± 13.2^a^ | 154 ± 18.4^abc^ | 165 ± 25^ab^ | 159 ± 19.1^ab^ | 72.5 ± 6.71^cd^ | 75.2 ± 10.6^cd^ | 108 ± 5.6^bd^ | 75.2 ± 8.36^cd^ | 66.8 ± 2.16^d^ | 0.645 | 0.000 | 0.284 |
| **GCY** | kg ha^-1^ | 3120 ± 141^a^ | 3190 ± 401^a^ | 3070 ± 418^a^ | 3320 ± 474^a^ | 3080 ± 368^a^ | 1110 ± 103^b^ | 1050 ± 121^b^ | 1480 ± 106^b^ | 1050 ± 118^b^ | 954 ± 45.9^b^ | 0.916 | 0.000 | 0.780 |
| **biomass** | kg ha^-1^ | 14100 ± 536^a^ | 14000 ± 473^a^ | 14400 ± 2700^a^ | 15100 ± 1270^a^ | 15300 ± 1740^a^ | 6950 ± 803^b^ | 6540 ± 116^b^ | 9190 ± 1320^ab^ | 6440 ± 955^b^ | 5900 ± 528^b^ | 0.780 | 0.000 | 0.553 |
| **HI** | g grain g biomass^-1^ | 0.418 ± 0.017^a^ | 0.362 ± 0.017^abc^ | 0.373 ± 0.014^ab^ | 0.35 ± 0.022^abc^ | 0.399 ± 0.008^ab^ | 0.276 ± 0.031^cde^ | 0.218 ± 0.022^e^ | 0.314 ± 0.009^bd^ | 0.247 ± 0.014^de^ | 0.247 ± 0.017^de^ | 0.016 | 0.000 | 0.094 |
| **plant.m2** | plants m^-2^ | 180 ± 22 | 211 ± 25 | 201 ± 34 | 221 ± 22 | 243 ± 17 | 196 ± 6 | 175 ± 1 | 187 ± 28 | 212 ± 10 | 224 ± 12 | 0.163 | 0.340 | 0.784 |
| **ears.m2** | ears m^-2^ | 439 ± 22^ab^ | 535 ± 22^a^ | 339 ± 99^ab^ | 521 ± 31.2^a^ | 433 ± 58.7^ab^ | 368 ± 26^ab^ | 360 ± 21.2^ab^ | 371 ± 49.7^ab^ | 404 ± 20^ab^ | 281 ± 21.8^b^ | 0.072 | 0.003 | 0.187 |
| **ears.plant** | ears plant^-1^ | 2.49 ± 0.23^a^ | 2.6 ± 0.28^a^ | 1.65 ± 0.26^ab^ | 2.37 ± 0.10^a^ | 1.83 ± 0.35^ab^ | 2.04 ± 0.26^ab^ | 2.06 ± 0.14^ab^ | 2.00 ± 0.10^ab^ | 1.91 ± 0.09^ab^ | 1.26 ± 0.10^b^ | 0.006 | 0.020 | 0.181 |
| **grains.ear** | grains ear^-1^ | 27.3 ± 3.7^ac^ | 24.9 ± 5.1^ac^ | 28.3 ± 4.3^ab^ | 22.9 ± 2.3^ac^ | 30.4 ± 2.0^a^ | 19.2 ± 3.5^ac^ | 13.6 ± 0.5^bc^ | 21.5 ± 2.8^ac^ | 12.1 ± 2.1^c^ | 17.3 ± 2.2^ac^ | 0.119 | 0.000 | 0.850 |
| **d15N.grain** | ‰ | 2.19 ± 0.14^ac^ | 1.69 ± 0.10^ad^ | 2.44 ± 0.26^ab^ | 2.79 ± 0.06^a^ | 2.78 ± 0.32^a^ | 1.42 ± 0.23^bcd^ | 0.98 ± 0.17^cd^ | 0.95 ± 0.53^cd^ | 0.74 ± 0.24^d^ | 0.83 ± 0.18^d^ | 0.336 | 0.000 | 0.040 |
| **d13C.grain** | ‰ | -24.5 ± 0.8^be^ | -24.9 ± 0.7^cde^ | -25.6 ± 0.7^e^ | -26.1 ± 0.3^e^ | -25.2 ± 0.3^de^ | -22.4 ± 0.4^abc^ | -22.2 ± 0.7^ab^ | -22.3 ± 0.4^ab^ | -22.8 ± 0.2^abd^ | -21.8 ± 0.9^a^ | 0.299 | 0.000 | 0.628 |
| **prot.grain** | % | 13.0 ± 0.5^c^ | 14.5 ± 1.1^bc^ | 15.4 ± 0.6^ac^ | 14.8 ± 0.5^ac^ | 12.5 ± 0.5^c^ | 15.5 ± 0.8^ac^ | 17.7 ± 0.7^a^ | 16.3 ± 0.3^ab^ | 16.5 ± 0.5^ab^ | 17.2 ± 0.2^ab^ | 0.040 | 0.000 | 0.057 |
| **TW.grain** | g L^-1^ | 78.9 ± 1.35^ab^ | 80.5 ± 0.8^a^ | 78.8 ± 0.6^ab^ | 76.7 ± 0.2^b^ | 79.5 ± 0.4^ab^ | 78.3 ± 0.5^ab^ | 79.0 ± 0.5^ab^ | 80.3 ± 0.2^a^ | 77.7 ± 0.2^ab^ | 79.0 ± 0.1^ab^ | 0.004 | 0.945 | 0.116 |
| **TKW.grain** | g | 52.8 ± 1.5^bc^ | 55.5 ± 2.0^ab^ | 61.2 ± 1.2^a^ | 56.3 ± 3.5^ab^ | 51.1 ± 1.8^bc^ | 37.2 ± 0.1^d^ | 39.6 ± 0.9^d^ | 44.9 ± 0.2^cd^ | 39.6 ± 1.3^d^ | 38.0 ± 0.7^d^ | 0.000 | 0.000 | 0.819 |
| **vitreousness.grain** | % | 81.2 ± 9.3 | 92.2 ± 4.2 | 93.0 ± 2.8 | 80.2 ± 8.0 | 86.3 ± 10.7 | 97.8 ± 0.3 | 98.0 ± 0.3 | 98.2 ± 0.6 | 97.5 ± 0.5 | 97.8 ± 0.2 | 0.628 | 0.004 | 0.688 |
| **b.grain** |  | 17.8 ± 0.3^ad^ | 16.6 ± 0.4^cde^ | 15.6 ± 0.5^e^ | 17.0 ± 0.9^bde^ | 18.6 ± 0.2^abc^ | 17.8 ± 0.0^ad^ | 16.4 ± 0.3^de^ | 15.9 ± 0.2^de^ | 19.4 ± 0.5^a^ | 19.0 ± 0.3^ab^ | 0.000 | 0.041 | 0.033 |
| **SDSS.grain** | mL g^-1^ | 33.0 ± 1.5^c^ | 37.7 ± 0.3^bc^ | 33.0 ± 1.2^c^ | 38.7 ± 5.5^bc^ | 37.0 ± 4.7^bc^ | 47.0 ± 3.2^ab^ | 56.0 ± 1.5^a^ | 49.0 ± 0.6^ab^ | 59.0 ± 2.7^a^ | 58.3 ± 0.9^a^ | 0.011 | 0.000 | 0.668 |
| **WG.grain** | mg | 24.7 ± 1.1^cd^ | 28.8 ± 2.1^ad^ | 30.3 ± 2.2^ad^ | 26.9 ± 1.5^bcd^ | 23.6 ± 0.8^d^ | 31.5 ± 3.3^ad^ | 35.0 ± 1.3^ab^ | 31.3 ± 1.0^ad^ | 33.7 ± 1.7^ac^ | 36.3 ± 2.2^a^ | 0.383 | 0.000 | 0.072 |
| **GI.grain** | % | 74 ± 3.3^a^ | 71.7 ± 1.8^a^ | 54.4 ± 5.4^b^ | 71.6 ± 0.9^a^ | 50.4 ± 2.5^b^ | 77.1 ± 4.1^a^ | 85.6 ± 0.4^a^ | 80.9 ± 0.8^a^ | 82.1 ± 5.6^a^ | 49.8 ± 1.0^b^ | 0.000 | 0.000 | 0.003 |
| **N.grain** | % | 2.31 ± 0.34 | 2.75 ± 0.16 | 2.27 ± 0.35 | 2.19 ± 0.16 | 2.25 ± 0.02 | 2.89 ± 0.12 | 3.14 ± 0.10 | 3.22 ± 0.21 | 3.16 ± 0.32 | 3.07 ± 0.12 | 0.574 | 0.000 | 0.623 |
| **C.grain** | % | 43.2 ± 0.2 | 43.7 ± 0.4 | 43.6 ± 0.2 | 43.9 ± 0.3 | 43.6 ± 0.3 | 44.2 ± 0.5 | 43.8 ± 0.2 | 43.6 ± 0.1 | 43.4 ± 0.2 | 43.8 ± 0.1 | 0.986 | 0.368 | 0.159 |
| **Ca.grain** | g kg DW*^-1^* | 0.409 ± 0.012^ab^ | 0.387 ± 0.023^b^ | 0.338 ± 0.023^b^ | 0.333 ± 0.023^b^ | 0.42 ± 0.025^ab^ | 0.411 ± 0.020^ab^ | 0.363 ± 0.01^b^ | 0.324 ± 0.019^b^ | 0.384 ± 0.021^b^ | 0.506 ± 0.020^a^ | 0.000 | 0.127 | 0.058 |
| **K.grain** | g kg DW*^-1^* | 5.00 ± 0.17 | 4.73 ± 0.26 | 4.59 ± 0.06 | 4.69 ± 0.10 | 4.68 ± 0.11 | 4.71 ± 0.29 | 4.74 ± 0.25 | 4.03 ± 0.11 | 4.52 ± 0.34 | 4.93 ± 0.32 | 0.144 | 0.295 | 0.471 |
| **Mg.grain** | g kg DW*^-1^* | 1.32 ± 0.05^bcd^ | 1.42 ± 0.10^ab^ | 1.64 ± 0.09^a^ | 1.42 ± 0.04^ac^ | 1.19 ± 0.02^bcd^ | 1.13 ± 0.02^d^ | 1.15 ± 0.05^cd^ | 1.09 ± 0.01^d^ | 1.15 ± 0.03^bcd^ | 1.15 ± 0.05^cd^ | 0.019 | 0.000 | 0.003 |
| **P.grain** | g kg DW*^-1^* | 3.32 ± 0.15^bc^ | 3.79 ± 0.34^ab^ | 4.45 ± 0.27^a^ | 3.66 ± 0.19^ac^ | 2.76 ± 0.06^bc^ | 2.84 ± 0.25^bc^ | 3.07 ± 0.16^bc^ | 2.67 ± 0.02^c^ | 2.87 ± 0.11^bc^ | 2.94 ± 0.30^bc^ | 0.023 | 0.000 | 0.003 |
| **S.grain** | g kg DW*^-1^* | 1.69 ± 0.14^c^ | 1.77 ± 0.07^c^ | 1.83 ± 0.06^c^ | 1.88 ± 0.15^c^ | 1.89 ± 0.03^c^ | 1.99 ± 0.08^ac^ | 2.17 ± 0.13^ac^ | 1.9 ± 0.04^bc^ | 2.39 ± 0.08^ab^ | 2.41 ± 0.11^a^ | 0.010 | 0.000 | 0.164 |
| **Cu.grain** | mg kg DW*^-1^* | 3.76 ± 0.21 | 4.46 ± 0.32 | 5.54 ± 0.44 | 4.59 ± 0.76 | 3.87 ± 0.13 | 3.68 ± 0.17 | 4.04 ± 0.38 | 3.84 ± 0.35 | 4.06 ± 0.23 | 3.77 ± 0.34 | 0.102 | 0.027 | 0.215 |
| **Fe.grain** | mg kg DW*^-1^* | 33.7 ± 3.7^b^ | 44.7 ± 3.9^ab^ | 58.8 ± 8.6^a^ | 43.8 ± 2.9^ab^ | 34.4 ± 3.3^b^ | 35.1 ± 3.0^b^ | 39.4 ± 2.2^ab^ | 38.5 ± 1.8^b^ | 42.0 ± 2.23^ab^ | 37.6 ± 3.6^b^ | 0.013 | 0.086 | 0.055 |
| **Mn.grain** | mg kg DW*^-1^* | 32.5 ± 2.1^bd^ | 36.8 ± 1.1^bc^ | 46.1 ± 3.7^a^ | 38.9 ± 1.6^ab^ | 23.4 ± 0.7^ef^ | 25.6 ± 0.8^df^ | 30.3 ± 1.4^cde^ | 27.0 ± 1.5^df^ | 25.6 ± 1.3^df^ | 20.6 ± 0.5^f^ | 0.000 | 0.000 | 0.001 |
| **Zn.grain** | mg kg DW*^-1^* | 14.4 ± 1.2 | 19.6 ± 0.7 | 22.9 ± 2.0 | 16.2 ± 2.7 | 15.8 ± 2.0 | 20.0 ± 3.0 | 19.1 ± 1.4 | 14.5 ± 1.4 | 16.9 ± 1.4 | 22.9 ± 1.8 | 0.472 | 0.447 | 0.005 |
| **NDVI_25** |  | 0.437 ± 0.041^ab^ | 0.410 ± 0.045^ab^ | 0.390 ± 0.025^b^ | 0.460 ± 0.023^ab^ | 0.400 ± 0.027^ab^ | 0.483 ± 0.018^ab^ | 0.523 ± 0.009^a^ | 0.470 ± 0.020^ab^ | 0.520 ± 0.015^ab^ | 0.467 ± 0.019^ab^ | 0.170 | 0.000 | 0.760 |
| **NDVI_35** |  | 0.660 ± 0.025^ab^ | 0.650 ± 0.017^ab^ | 0.670 ± 0.021^ab^ | 0.703 ± 0.022^a^ | 0.617 ± 0.035^ab^ | 0.630 ± 0.030^ab^ | 0.670 ± 0.015^ab^ | 0.663 ± 0.009^ab^ | 0.667 ± 0.032^ab^ | 0.577 ± 0.017^b^ | 0.015 | 0.226 | 0.688 |
| **NDVI_40** |  | 0.783 ± 0.009^ab^ | 0.767 ± 0.018^abc^ | 0.790 ± 0.012^a^ | 0.793 ± 0.009^a^ | 0.757 ± 0.017^abd^ | 0.643 ± 0.037^de^ | 0.670 ± 0.023^be^ | 0.720 ± 0.000^abd^ | 0.660 ± 0.040^cde^ | 0.563 ± 0.032^e^ | 0.010 | 0.000 | 0.128 |
| **NDVI_45** |  | 0.763 ± 0.015^ac^ | 0.710 ± 0.047^ad^ | 0.783 ± 0.023^a^ | 0.773 ± 0.007^ab^ | 0.743 ± 0.015^ac^ | 0.633 ± 0.037^cd^ | 0.633 ± 0.020^cd^ | 0.713 ± 0.003^ad^ | 0.643 ± 0.026^bcd^ | 0.587 ± 0.038^d^ | 0.040 | 0.000 | 0.440 |
| **NDVI_55** |  | 0.747 ± 0.024^a^ | 0.693 ± 0.061^ab^ | 0.760 ± 0.027^a^ | 0.743 ± 0.017^a^ | 0.743 ± 0.012^a^ | 0.577 ± 0.052^bc^ | 0.560 ± 0.017^bc^ | 0.680 ± 0.010^ab^ | 0.543 ± 0.017^bc^ | 0.490 ± 0.029^c^ | 0.027 | 0.000 | 0.103 |
| **NDVI_65** |  | 0.697 ± 0.015^a^ | 0.683 ± 0.041^a^ | 0.677 ± 0.054^a^ | 0.687 ± 0.044^a^ | 0.680 ± 0.015^a^ | 0.290 ± 0.023^b^ | 0.320 ± 0.020^b^ | 0.320 ± 0.021^b^ | 0.270 ± 0.027^b^ | 0.283 ± 0.018^b^ | 0.920 | 0.000 | 0.818 |


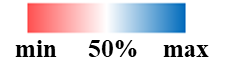
**Supplementary Table S1**. Continued.

| **Variable** | **Units** | **Irrigated** | | | | | **Rainfed** | | | | | ***P*-value** | | |
| --- | --- | --- | --- | --- | --- | --- | --- | --- | --- | --- | --- | --- | --- | --- |
|  |  | **MEX** | **EUR** | **DRI** | **KNI** | **HAR** | **MEX** | **EUR** | **DRI** | **KNI** | **HAR** | **G** | **W** | **G×W** |
| **Chl_55** |  | 43.6 ± 2.5^bc^ | 48.7 ± 1.87^ab^ | 52.1 ± 1.3^a^ | 44.9 ± 0.4^ac^ | 40.8 ± 0.9^bc^ | 46.2 ± 0.6^ab^ | 45.1 ± 0.4^ab^ | 46.9 ± 1.1^ab^ | 46.9 ± 1.1^ab^ | 36.8 ± 3.3^c^ | 0.000 | 0.133 | 0.084 |
| **Chl_65** |  | 40.1 ± 2.0^ab^ | 48.0 ± 1.9^a^ | 49.3 ± 1.6^a^ | 43.9 ± 1.5^ab^ | 37.4 ± 2.2^ab^ | 32.8 ± 2.3^b^ | 37.2 ± 5.0^ab^ | 38.2 ± 3.2^ab^ | 38.3 ± 4.4^ab^ | 37.1 ± 2.4^ab^ | 0.070 | 0.001 | 0.353 |
| **Chl_75** |  | 37.5 ± 0.6^ab^ | 36.3 ± 3.1^ab^ | 45.8 ± 2.4^a^ | 37.0 ± 3.0^ab^ | 42.2 ± 2.6^a^ | 24.5 ± 3.8^bc^ | 32.1 ± 3.8^ac^ | 30.1 ± 4.3^ac^ | 18.1 ± 2.1^c^ | 35.1 ± 6.2^ac^ | 0.022 | 0.000 | 0.235 |
| **Flav_55** |  | 1.45 ± 0.04^ac^ | 1.54 ± 0.03^ab^ | 1.54 ± 0.04^ab^ | 1.58 ± 0.01^a^ | 1.35 ± 0.05^c^ | 1.44 ± 0.06^ac^ | 1.49 ± 0.02^ac^ | 1.55 ± 0.03^ab^ | 1.58 ± 0.04^a^ | 1.39 ± 0.02^bc^ | 0.000 | 0.917 | 0.777 |
| **Flav_65** |  | 1.42 ± 0.03 | 1.49 ± 0.04 | 1.51 ± 0.01 | 1.49 ± 0.01 | 1.39 ± 0.04 | 1.46 ± 0.01 | 1.51 ± 0.03 | 1.54 ± 0.05 | 1.55 ± 0.03 | 1.48 ± 0.05 | 0.029 | 0.041 | 0.845 |
| **Flav_75** |  | 1.48 ± 0.05^ab^ | 1.53 ± 0.04^ab^ | 1.45 ± 0.03^ab^ | 1.38 ± 0.06^b^ | 1.36 ± 0.04^b^ | 1.57 ± 0.05^ab^ | 1.56 ± 0.07^ab^ | 1.6 ± 0.05^a^ | 1.65 ± 0.02^a^ | 1.55 ± 0.04^ab^ | 0.337 | 0.000 | 0.099 |
| **Anth_55** |  | 0.113 ± 0.011 | 0.098 ± 0.007 | 0.0893 ± 0.006 | 0.111 ± 0.007 | 0.112 ± 0.003 | 0.107 ± 0.004 | 0.099 ± 0.007 | 0.103 ± 0.006 | 0.115 ± 0.008 | 0.108 ± 0.011 | 0.125 | 0.695 | 0.735 |
| **Anth_65** |  | 0.100 ± 0.004 | 0.109 ± 0.007 | 0.110 ± 0.009 | 0.108 ± 0.012 | 0.144 ± 0.005 | 0.134 ± 0.013 | 0.118 ± 0.012 | 0.113 ± 0.013 | 0.114 ± 0.008 | 0.133 ± 0.017 | 0.093 | 0.239 | 0.370 |
| **Anth_75** |  | 0.098 ± 0.018^bc^ | 0.086 ± 0.004^bc^ | 0.071 ± 0.007^c^ | 0.098 ± 0.004^bc^ | 0.118 ± 0.015^bc^ | 0.199 ± 0.026^ab^ | 0.171 ± 0.012^ac^ | 0.190 ± 0.030^ac^ | 0.289 ± 0.037^a^ | 0.186 ± 0.043^ac^ | 0.080 | 0.000 | 0.126 |
| **NBI_55** |  | 30.2 ± 1.6^ab^ | 31.7 ± 1.9^ab^ | 34.0 ± 1.7^a^ | 28.7 ± 0.4^ab^ | 30.7 ± 1.7^ab^ | 32.2 ± 1.0^ab^ | 30.7 ± 0.4^ab^ | 30.4 ± 1.5^ab^ | 29.7 ± 1.4^ab^ | 26.3 ± 2.4^b^ | 0.121 | 0.231 | 0.194 |
| **NBI_65** |  | 28.4 ± 1.1 | 32.4 ± 2.0 | 32.5 ± 1.3 | 29.6 ± 1.6 | 27 ± 2.5 | 22.3 ± 1.7 | 24.3 ± 3.2 | 24.6 ± 1.5 | 24.4 ± 2.7 | 25.4 ± 2.5 | 0.512 | 0.000 | 0.543 |
| **NBI_75** |  | 25.5 ± 1.2^ab^ | 23.6 ± 1.9^ab^ | 32.0 ± 0.8^a^ | 27.0 ± 2.8^ab^ | 31.7 ± 1.3^a^ | 15.5 ± 2.7^bc^ | 20.1 ± 2.1^ac^ | 19.1 ± 3.2^bc^ | 10.7 ± 1.4^c^ | 22.9 ± 4.3^ab^ | 0.011 | 0.000 | 0.135 |
| **RWC_55** | % | 85.1 ± 0.3^ac^ | 88.9 ± 1.7^ac^ | 92.1 ± 1.4^a^ | 85.6 ± 2.9^ac^ | 80.0 ± 1.3^cd^ | 88.4 ± 1.7^ac^ | 80.3 ± 1.7^cd^ | 81.8 ± 2.5^bcd^ | 90.6 ± 0.8^ab^ | 74.4 ± 2.5^d^ | 0.000 | 0.012 | 0.001 |
| **RWC_65** | % | 82.3 ± 2.1^ac^ | 87.0 ± 2.6^ab^ | 89.8 ± 1.2^a^ | 87.8 ± 1.7^ab^ | 82.6 ± 1.2^ac^ | 72.2 ± 4.9^c^ | 81.3 ± 1.9^ac^ | 83.3 ± 3.7^ac^ | 72.5 ± 1.5^c^ | 76.5 ± 2.6^bc^ | 0.012 | 0.000 | 0.323 |
| **RWC_75** | % | 80.9 ± 3.4^ab^ | 83.2 ± 4.8^a^ | 85.6 ± 1.8^a^ | 77.9 ± 6.4^ab^ | 80.9 ± 2.1^ab^ | 62.7 ± 4.4^b^ | 71.7 ± 2.0^ab^ | 73.5 ± 1.5^ab^ | 62.3 ± 5.3^b^ | 75.8 ± 3.8^ab^ | 0.088 | 0.000 | 0.539 |
| **FW.b.65** | g | 0.323 ± 0.020^a^ | 0.275 ± 0.035^ac^ | 0.280 ± 0.026^ab^ | 0.255 ± 0.010^ad^ | 0.38 ± 0.026^a^ | 0.186 ± 0.047^bcd^ | 0.152 ± 0.008^cd^ | 0.192 ± 0.011^bcd^ | 0.137 ± 0.027^d^ | 0.196 ± 0.010^bcd^ | 0.014 | 0.000 | 0.438 |
| **FW.s.65** | g | 0.461 ± 0.030^a^ | 0.374 ± 0.037^ad^ | 0.403 ± 0.029^abc^ | 0.409 ± 0.018^abc^ | 0.437 ± 0.021^ab^ | 0.342 ± 0.027^ad^ | 0.276 ± 0.001^d^ | 0.330 ± 0.023^bd^ | 0.292 ± 0.032^cd^ | 0.340 ± 0.016^ad^ | 0.046 | 0.000 | 0.884 |
| **FW.p.65** | g | 0.968 ± 0.260^ac^ | 1.00 ± 0.115^ac^ | 1.100 ± 0.028^ab^ | 1.210 ± 0.048^a^ | 0.766 ± 0.004^ac^ | 0.637 ± 0.165^bc^ | 0.466 ± 0.015^c^ | 0.603 ± 0.099^bc^ | 0.594 ± 0.090^bc^ | 0.507 ± 0.013^c^ | 0.202 | 0.000 | 0.516 |
| **FW.a.65** | g | 0.409 ± 0.066^a^ | 0.275 ± 0.025^ac^ | 0.251 ± 0.028^bc^ | 0.268 ± 0.042^ac^ | 0.368 ± 0.036^ab^ | 0.255 ± 0.008^ac^ | 0.258 ± 0.024^ac^ | 0.239 ± 0.013^bc^ | 0.158 ± 0.010^c^ | 0.258 ± 0.004^ac^ | 0.007 | 0.001 | 0.132 |
| **FW.g.65** | g | 0.244 ± 0.030^a^ | 0.161 ± 0.011^bc^ | 0.252 ± 0.013^a^ | 0.206 ± 0.022^ac^ | 0.246 ± 0.013^a^ | 0.159 ± 0.003^bc^ | 0.157 ± 0.006^bc^ | 0.222 ± 0.008^ab^ | 0.138 ± 0.006^c^ | 0.221 ± 0.005^ab^ | 0.000 | 0.000 | 0.057 |
| **FW.l.65** | g | 0.369 ± 0.052^a^ | 0.235 ± 0.014^bd^ | 0.310 ± 0.014^abc^ | 0.290 ± 0.0378^abc^ | 0.338 ± 0.029^ab^ | 0.23 ± 0.009^bd^ | 0.208 ± 0.004^cd^ | 0.284 ± 0.010^ad^ | 0.164 ± 0.012^d^ | 0.226 ± 0.010^bd^ | 0.006 | 0.000 | 0.064 |
| **FW.e.65** | g | 1.440 ± 0.083^ab^ | 1.270 ± 0.120^bc^ | 1.340 ± 0.086^ab^ | 1.210 ± 0.082^bc^ | 1.680 ± 0.075^a^ | 1.240 ± 0.053^bc^ | 1.100 ± 0.042^bc^ | 1.080 ± 0.063^bc^ | 0.879 ± 0.104^c^ | 1.220 ± 0.037^bc^ | 0.001 | 0.000 | 0.361 |
| **FW.b.75** | g | 0.239 ± 0.020^ac^ | 0.271 ± 0.001^ac^ | 0.333 ± 0.011^ab^ | 0.261 ± 0.038^ac^ | 0.375 ± 0.070^a^ | 0.119 ± 0.016^c^ | 0.149 ± 0.012^bc^ | 0.191 ± 0.020^ac^ | 0.116 ± 0.035^c^ | 0.206 ± 0.089^ac^ | 0.052 | 0.000 | 0.975 |
| **FW.s.75** | g | 0.322 ± 0.011^ab^ | 0.280 ± 0.008^ab^ | 0.400 ± 0.029^ab^ | 0.314 ± 0.042^ab^ | 0.425 ± 0.033^a^ | 0.258 ± 0.011^ab^ | 0.273 ± 0.008^ab^ | 0.348 ± 0.033^ab^ | 0.246 ± 0.037^b^ | 0.362 ± 0.077^ab^ | 0.005 | 0.033 | 0.907 |
| **FW.p.75** | g | 0.876 ± 0.120^ac^ | 0.781 ± 0.053^ac^ | 1.070 ± 0.027^ab^ | 0.936 ± 0.148^ac^ | 1.140 ± 0.139^a^ | 0.439 ± 0.056^c^ | 0.435 ± 0.022^c^ | 0.591 ± 0.056^bc^ | 0.489 ± 0.096^c^ | 0.678 ± 0.161^ac^ | 0.040 | 0.000 | 0.969 |
| **FW.a.75** | g | 0.288 ± 0.009^ac^ | 0.279 ± 0.048^ac^ | 0.210 ± 0.010^c^ | 0.214 ± 0.032^bc^ | 0.452 ± 0.075^ab^ | 0.271 ± 0.036^ac^ | 0.255 ± 0.014^ac^ | 0.235 ± 0.022^ac^ | 0.168 ± 0.010^c^ | 0.465 ± 0.109^a^ | 0.000 | 0.756 | 0.946 |
| **FW.g.75** | g | 0.172 ± 0.003^bc^ | 0.138 ± 0.015^c^ | 0.208 ± 0.012^ac^ | 0.159 ± 0.028^c^ | 0.264 ± 0.023^ab^ | 0.172 ± 0.012^bc^ | 0.153 ± 0.004^c^ | 0.211 ± 0.017^ac^ | 0.148 ± 0.007^c^ | 0.280 ± 0.038^a^ | 0.000 | 0.689 | 0.950 |
| **FW.l.75** | g | 0.234 ± 0.006 | 0.179 ± 0.031 | 0.239 ± 0.018 | 0.182 ± 0.032 | 0.287 ± 0.035 | 0.196 ± 0.010 | 0.196 ± 0.003 | 0.235 ± 0.019 | 0.167 ± 0.014 | 0.282 ± 0.049 | 0.003 | 0.581 | 0.876 |
| **FW.e.75** | g | 2.40 ± 0.37^ab^ | 2.02 ± 0.25^ab^ | 2.87 ± 0.44^ab^ | 1.76 ± 0.18^b^ | 3.88 ± 0.47^a^ | 1.71 ± 0.26^b^ | 1.63 ± 0.05^b^ | 2.24 ± 0.15^ab^ | 1.54 ± 0.25^b^ | 3.87 ± 0.79^a^ | 0.000 | 0.120 | 0.886 |
| **DW.b.65** | g | 0.094 ± 0.004^ab^ | 0.089 ± 0.011^ac^ | 0.096 ± 0.007^ab^ | 0.076 ± 0.003^bc^ | 0.122 ± 0.008^a^ | 0.069 ± 0.013^bc^ | 0.063 ± 0.003^bc^ | 0.071 ± 0.010^bc^ | 0.054 ± 0.010^c^ | 0.075 ± 0.003^bc^ | 0.007 | 0.000 | 0.520 |
| **DW.s.65** | g | 0.160 ± 0.008 | 0.140 ± 0.014 | 0.161 ± 0.011 | 0.143 ± 0.009 | 0.163 ± 0.007 | 0.148 ± 0.010 | 0.126 ± 0.001 | 0.151 ± 0.009 | 0.127 ± 0.014 | 0.146 ± 0.006 | 0.047 | 0.034 | 0.994 |
| **DW.p.65** | g | 0.257 ± 0.004^ac^ | 0.272 ± 0.014^ab^ | 0.291 ± 0.018^a^ | 0.238 ± 0.002^ad^ | 0.194 ± 0.012^bcd^ | 0.207 ± 0.028^bcd^ | 0.185 ± 0.007^cd^ | 0.182 ± 0.020^cd^ | 0.199 ± 0.027^bcd^ | 0.168 ± 0.006^d^ | 0.019 | 0.000 | 0.100 |
| **DW.a.65** | g | 0.152 ± 0.009^ab^ | 0.140 ± 0.014^ac^ | 0.114 ± 0.010^bcd^ | 0.114 ± 0.007^bcd^ | 0.169 ± 0.009^a^ | 0.137 ± 0.004^ac^ | 0.107 ± 0.004^cd^ | 0.103 ± 0.008^cd^ | 0.080 ± 0.012^d^ | 0.124 ± 0.007^bcd^ | 0.000 | 0.000 | 0.299 |
| **DW.g.65** | g | 0.066 ± 0.003^bcd^ | 0.063 ± 0.007^cd^ | 0.084 ± 0.006^ab^ | 0.063 ± 0.004^cd^ | 0.092 ± 0.001^a^ | 0.068 ± 0.004^bcd^ | 0.058 ± 0.003^d^ | 0.081 ± 0.003^ac^ | 0.059 ± 0.004^d^ | 0.083 ± 0.003^ab^ | 0.000 | 0.169 | 0.777 |
| **DW.l.65** | g | 0.085 ± 0.0041^ab^ | 0.080 ± 0.012^ab^ | 0.101 ± 0.007^a^ | 0.077 ± 0.005^ab^ | 0.099 ± 0.002^a^ | 0.080 ± 0.005^ab^ | 0.067 ± 0.002^b^ | 0.087 ± 0.002^ab^ | 0.065 ± 0.008^b^ | 0.068 ± 0.006^b^ | 0.008 | 0.001 | 0.303 |
| **DW.e.65** | g | 0.463 ± 0.021^ac^ | 0.445 ± 0.043^ac^ | 0.481 ± 0.039^ac^ | 0.387 ± 0.023^bc^ | 0.548 ± 0.020^a^ | 0.491 ± 0.024^ab^ | 0.437 ± 0.014^ac^ | 0.445 ± 0.017^ac^ | 0.346 ± 0.041^c^ | 0.478 ± 0.013^ac^ | 0.001 | 0.167 | 0.476 |
| **DW.b.75** | g | 0.084 ± 0.001^ac^ | 0.100 ± 0.003^ac^ | 0.130 ± 0.007^ab^ | 0.085 ± 0.008^ac^ | 0.139 ± 0.019^a^ | 0.055 ± 0.005^c^ | 0.067 ± 0.006^bc^ | 0.089 ± 0.010^ac^ | 0.047 ± 0.011^c^ | 0.097 ± 0.033^ac^ | 0.003 | 0.000 | 0.983 |
| **DW.s.75** | g | 0.133 ± 0.010 | 0.123 ± 0.006 | 0.180 ± 0.013 | 0.130 ± 0.016 | 0.193 ± 0.014 | 0.126 ± 0.006 | 0.133 ± 0.005 | 0.172 ± 0.017 | 0.115 ± 0.015 | 0.171 ± 0.034 | 0.001 | 0.395 | 0.878 |
| **DW.p.75** | g | 0.373 ± 0.058^ac^ | 0.331 ± 0.023^ac^ | 0.456 ± 0.014^ab^ | 0.395 ± 0.050^ac^ | 0.522 ± 0.064^a^ | 0.207 ± 0.029^c^ | 0.201 ± 0.011^c^ | 0.278 ± 0.028^bc^ | 0.226 ± 0.043^c^ | 0.306 ± 0.066^bc^ | 0.017 | 0.000 | 0.902 |
| **DW.a.75** | g | 0.163 ± 0.021^ab^ | 0.150 ± 0.014^ab^ | 0.156 ± 0.013^ab^ | 0.120 ± 0.013^ab^ | 0.237 ± 0.024^a^ | 0.140 ± 0.009^ab^ | 0.132 ± 0.003^ab^ | 0.132 ± 0.015^ab^ | 0.078 ± 0.015^b^ | 0.237 ± 0.060^a^ | 0.000 | 0.169 | 0.935 |
| **DW.g.75** | g | 0.076 ± 0.008^bc^ | 0.060 ± 0.007^c^ | 0.110 ± 0.008^ab^ | 0.066 ± 0.006^c^ | 0.114 ± 0.009^a^ | 0.067 ± 0.004^c^ | 0.060 ± 0.003^c^ | 0.107 ± 0.006^ab^ | 0.057 ± 0.006^c^ | 0.116 ± 0.011^a^ | 0.000 | 0.374 | 0.912 |
| **DW.l.75** | g | 0.091 ± 0.009^ac^ | 0.087 ± 0.011^ac^ | 0.129 ± 0.011^a^ | 0.082 ± 0.012^ac^ | 0.119 ± 0.005^ab^ | 0.077 ± 0.007^ac^ | 0.069 ± 0.004^bc^ | 0.116 ± 0.006^ab^ | 0.058 ± 0.011^c^ | 0.114 ± 0.020^ab^ | 0.000 | 0.039 | 0.928 |
| **DW.e.75** | g | 0.910 ± 0.154^bc^ | 0.758 ± 0.097^c^ | 1.100 ± 0.172^ac^ | 0.632 ± 0.060^c^ | 1.570 ± 0.211^ab^ | 0.679 ± 0.091^c^ | 0.631 ± 0.017^c^ | 0.895 ± 0.056^bc^ | 0.580 ± 0.092^c^ | 1.680 ± 0.287^a^ | 0.000 | 0.286 | 0.770 |
| **WC.b.65** | % | 70.7 ± 0.8^a^ | 67.7 ± 0.8^ab^ | 65.7 ± 1.0^ab^ | 70.1 ± 0.3^a^ | 67.8 ± 0.2^ab^ | 61.6 ± 2.3^ab^ | 58.7 ± 0.2^b^ | 63.2 ± 4.9^ab^ | 60.3 ± 1.2^b^ | 61.6 ± 0.5^ab^ | 0.603 | 0.000 | 0.276 |
| **WC.s.65** | % | 65.3 ± 0.6^a^ | 62.4 ± 0.7^b^ | 60.1 ± 0.8^b^ | 65.2 ± 0.8^a^ | 62.6 ± 0.36^ab^ | 56.7 ± 0.6^c^ | 54.3 ± 0.1^c^ | 54.2 ± 0.4^c^ | 56.6 ± 0.4^c^ | 56.9 ± 0.3^c^ | 0.000 | 0.000 | 0.022 |
| **WC.p.65** | % | 67.1 ± 11.8 | 72.5 ± 1.9 | 73.4 ± 2.3 | 80.2 ± 0.7 | 74.7 ± 1.6 | 75.0 ± 6.7 | 60.3 ± 0.2 | 69.2 ± 1.7 | 66.3 ± 0.8 | 66.8 ± 0.5 | 0.641 | 0.045 | 0.153 |
| **WC.a.65** | % | 61.2 ± 4.8 | 48.4 ± 6.9 | 53.7 ± 5.9 | 56 ± 4.3 | 53.5 ± 2.4 | 46.1 ± 3.4 | 58.2 ± 3.1 | 57.1 ± 1.4 | 50.1 ± 5.1 | 52.0 ± 3.4 | 0.975 | 0.507 | 0.090 |
| **WC.g.65** | % | 72.5 ± 2.6^a^ | 60.8 ± 5.1^ab^ | 66.6 ± 3.1^ab^ | 69.2 ± 1.9^ab^ | 62.6 ± 1.6^ab^ | 57.5 ± 2.1^b^ | 62.9 ± 2.7^ab^ | 63.7 ± 0.5^ab^ | 57.2 ± 2.0^b^ | 62.4 ± 0.7^ab^ | 0.620 | 0.002 | 0.011 |
| **WC.l.65** | % | 76.1 ± 2.9^a^ | 65.7 ± 5.9^ab^ | 67.3 ± 1.9^ab^ | 72.8 ± 2.4^ab^ | 70.2 ± 2.3^ab^ | 65.2 ± 1.0^ab^ | 67.7 ± 1.3^ab^ | 69.3 ± 1.2^ab^ | 60.7 ± 3.2^b^ | 70 ± 1.9^ab^ | 0.494 | 0.040 | 0.032 |
| **WC.e.65** | % | 67.8 ± 0.5^a^ | 64.9 ± 0.6^ab^ | 64.2 ± 0.8^b^ | 67.9 ± 0.4^a^ | 67.3 ± 1.0^ab^ | 60.5 ± 0.9^c^ | 60.3 ± 0.2^c^ | 58.6 ± 0.9^c^ | 60.6 ± 0.6^c^ | 60.6 ± 0.3^c^ | 0.001 | 0.000 | 0.244 |


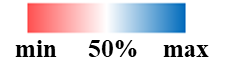
**Supplementary Table S1**. Continued.

| **Variable** | **Units** | **Irrigated** | | | | | **Rainfed** | | | | | ***P*-value** | | |
| --- | --- | --- | --- | --- | --- | --- | --- | --- | --- | --- | --- | --- | --- | --- |
|  |  | **MEX** | **EUR** | **DRI** | **KNI** | **HAR** | **MEX** | **EUR** | **DRI** | **KNI** | **HAR** | **G** | **W** | **G×W** |
| **WC.b.75** | % | 64.5 ± 2.8^ab^ | 63.2 ± 0.9^ab^ | 61.1 ± 1.3^ab^ | 66.7 ± 2.2^a^ | 62.1 ± 2.5^ab^ | 52.8 ± 2.6^ab^ | 54.9 ± 0.4^ab^ | 53.7 ± 0.5^ab^ | 56.9 ± 4.6^ab^ | 44.6 ± 10.8^b^ | 0.370 | 0.000 | 0.746 |
| **WC.s.75** | % | 58.8 ± 1.7^a^ | 56.2 ± 0.7^ab^ | 54.9 ± 0.8^ac^ | 58.2 ± 2.0^a^ | 54.5 ± 0.3^ac^ | 51.1 ± 0.3^bc^ | 51.3 ± 0.3^bc^ | 50.5 ± 0.3^c^ | 53.0 ± 1.2^bc^ | 52.6 ± 0.7^bc^ | 0.074 | 0.000 | 0.132 |
| **WC.p.75** | % | 57.6 ± 0.7^a^ | 57.6 ± 0.2^a^ | 57.3 ± 1.0^a^ | 57.3 ± 1.7^a^ | 54.2 ± 0.6^ab^ | 53.2 ± 0.8^b^ | 53.7 ± 0.1^ab^ | 53.0 ± 0.3^b^ | 53.7 ± 0.3^ab^ | 54.4 ± 1.1^ab^ | 0.528 | 0.000 | 0.051 |
| **WC.a.75** | % | 42.9 ± 8.7 | 42.4 ± 12.1 | 26.3 ± 3.2 | 43.2 ± 3.6 | 46.3 ± 4.4 | 47.6 ± 3.4 | 47.8 ± 3.8 | 43.4 ± 5.5 | 53.1 ± 9.5 | 45.8 ± 14.9 | 0.510 | 0.160 | 0.844 |
| **WC.g.75** | % | 55.9 ± 4.9 | 55.5 ± 5.4 | 47.1 ± 0.5 | 57.5 ± 3.4 | 56.6 ± 0.4 | 61.1 ± 2.3 | 60.8 ± 2.7 | 49.1 ± 2.1 | 61.4 ± 5.2 | 57.0 ± 7.7 | 0.073 | 0.210 | 0.968 |
| **WC.l.75** | % | 60.8 ± 4.9 | 50 ± 6.1 | 45.9 ± 2.2 | 54 ± 3.6 | 57.7 ± 3.0 | 60.6 ± 3.5 | 64.6 ± 1.5 | 50.5 ± 1.9 | 64.6 ± 8.2 | 56.9 ± 11.3 | 0.217 | 0.111 | 0.568 |
| **WC.e.75** | % | 62.4 ± 0.7^a^ | 62.5 ± 0.3^a^ | 61.5 ± 0.1^a^ | 64.1 ± 1.1^a^ | 59.7 ± 2.0^ab^ | 60.0 ± 0.7^ab^ | 61.2 ± 0.2^a^ | 60.1 ± 0.2^ab^ | 62.2 ± 0.4^a^ | 55.9 ± 1.8^b^ | 0.000 | 0.002 | 0.700 |
| **Glc.b.65** | *µmol g DW^-1^* | 17.3 ± 1.1^bc^ | 10.8 ± 2.9^bc^ | 14.0 ± 5.8^bc^ | 7.5 ± 0.4^c^ | 43.1 ± 14.3^ab^ | 57.0 ± 9.2^a^ | 12.7 ± 2.4^bc^ | 32.7 ± 6.6^ac^ | 59.3 ± 6.3^a^ | 18.5 ± 4.7^bc^ | 0.009 | 0.000 | 0.000 |
| **Glc.s.65** | *µmol g DW^-1^* | 57.4 ± 4.2^abc^ | 32.5 ± 7.6^bd^ | 22.6 ± 9.2^cd^ | 44.2 ± 3.5^ad^ | 61.1 ± 8.0^ab^ | 73.5 ± 15.1^a^ | 16.0 ± 3.24^d^ | 42.0 ± 5.9^ad^ | 60.0 ± 3.5^ab^ | 55.6 ± 3.5^abc^ | 0.000 | 0.219 | 0.086 |
| **Glc.p.65** | *µmol g DW^-1^* | 227 ± 70^ab^ | 243 ± 40^ab^ | 222 ± 38^ab^ | 338 ± 28^a^ | 318 ± 40^a^ | 273 ± 41^a^ | 71 ± 10^b^ | 305 ± 22^a^ | 299 ± 38^a^ | 169 ± 15^ab^ | 0.007 | 0.068 | 0.009 |
| **Glc.a.65** | *µmol g DW^-1^* | 85.1 ± 19.4^ac^ | 36.8 ± 5.5^bc^ | 37.5 ± 3.5^bc^ | 75.7 ± 9.3^ac^ | 85.4 ± 3.4^ac^ | 113.0 ± 27^a^ | 28.3 ± 5.2^c^ | 67.5 ± 16.8^ac^ | 102.0 ± 7.8^ab^ | 50.0 ± 16.5^ac^ | 0.001 | 0.357 | 0.100 |
| **Glc.g.65** | *µmol g DW^-1^* | 123.0 ± 20.0^a^ | 69.0 ± 9.8^bc^ | 71.6 ± 0.8^ac^ | 121.0 ± 9.0^ab^ | 79.2 ± 0.9^ac^ | 94.4 ± 4.9^ac^ | 48.6 ± 8.9^c^ | 73.6 ± 2.8^ac^ | 95.9 ± 7.6^ac^ | 52.2 ± 17.7^c^ | 0.000 | 0.007 | 0.576 |
| **Glc.l.65** | *µmol g DW^-1^* | 199.0 ± 23.4^a^ | 89.1 ± 11.5^ce^ | 87.1 ± 13.4^de^ | 172.0 ± 17.9^ab^ | 167.0 ± 19.8^ac^ | 141.0 ± 6.75^acd^ | 42.7 ± 1.79^e^ | 99.9 ± 15.1^bce^ | 129.0 ± 13.1^acd^ | 105.0 ± 22.1^bce^ | 0.000 | 0.001 | 0.166 |
| **Glc.b.75** | *µmol g DW^-1^* | 44.9 ± 17.1^ab^ | 19.7 ± 4.7^b^ | 24.0 ± 9.32^b^ | 32.2 ± 17.1^b^ | 85.6 ± 9.3^a^ | 48.6 ± 10.0^ab^ | 19.2 ± 2.6^b^ | 23.1 ± 3.7^b^ | 29.2 ± 4.3^b^ | 14.6 ± 2.3^b^ | 0.014 | 0.028 | 0.004 |
| **Glc.s.75** | *µmol g DW^-1^* | 28.3 ± 5.7^ab^ | 14.7 ± 3.9^b^ | 12.6 ± 3.7^b^ | 19.8 ± 5.0^b^ | 41.7 ± 3.4^a^ | 20.9 ± 2.6^b^ | 14.7 ± 1.0^b^ | 13.2 ± 0.8^b^ | 17.8 ± 2.6^b^ | 24.2 ± 4.3^ab^ | 0.000 | 0.034 | 0.114 |
| **Glc.p.75** | *µmol g DW^-1^* | 37.1 ± 16.4 | 31.7 ± 15.0 | 31.4 ± 11.5 | 22.0 ± 7.24 | 40.6 ± 9.9 | 26.5 ± 2.8 | 19.2 ± 5.8 | 17.5 ± 3.5 | 18.6 ± 4.8 | 35.9 ± 14.9 | 0.471 | 0.185 | 0.980 |
| **Glc.a.75** | *µmol g DW^-1^* | 30.0 ± 9.1^ab^ | 25.1 ± 14.4^ab^ | 16.4 ± 5.1^b^ | 20.0 ± 6.6^b^ | 61.7 ± 7.3^a^ | 24.8 ± 3.2^ab^ | 20.0 ± 6.7^b^ | 11.9 ± 1.8^b^ | 18.8 ± 2.8^b^ | 34.1 ± 11.9^ab^ | 0.004 | 0.095 | 0.471 |
| **Glc.g.75** | *µmol g DW^-1^* | 41.6 ± 11.9 | 44.1 ± 15.4 | 40.4 ± 10.4 | 37.2 ± 7.7 | 44.8 ± 2.3 | 33.7 ± 5.7 | 28.4 ± 5.4 | 21.9 ± 5.8 | 15.4 ± 2.4 | 22.4 ± 6.8 | 0.685 | 0.004 | 0.908 |
| **Glc.l.75** | *µmol g DW^-1^* | 52.9 ± 14.5^ab^ | 35.0 ± 14.3^ab^ | 29.6 ± 4.9^ab^ | 29.4 ± 8.2^ab^ | 63.5 ± 4.9^a^ | 28.2 ± 3.0^ab^ | 21.2 ± 2.6^b^ | 19.6 ± 5.4^b^ | 25.2 ± 4.2^ab^ | 33.4 ± 10.9^ab^ | 0.045 | 0.006 | 0.537 |
| **Glc6P.b.65** | *µmol g DW^-1^* | 1.95 ± 0.14^a^ | 2.02 ± 0.13^a^ | 1.95 ± 0.11^a^ | 1.78 ± 0.14^a^ | 1.79 ± 0.19^a^ | 1.05 ± 0.05^b^ | 0.99 ± 0.02^b^ | 1.11 ± 0.11^b^ | 1.11 ± 0.09^b^ | 0.90 ± 0.03^b^ | 0.541 | 0.000 | 0.613 |
| **Glc6P.s.65** | *µmol g DW^-1^* | 1.48 ± 0.09^a^ | 1.28 ± 0.06^a^ | 1.30 ± 0.07^a^ | 1.45 ± 0.11^a^ | 1.20 ± 0.17^a^ | 0.71 ± 0.04^b^ | 0.57 ± 0.01^b^ | 0.59 ± 0.02^b^ | 0.75 ± 0.04^b^ | 0.66 ± 0.03^b^ | 0.062 | 0.000 | 0.674 |
| **Glc6P.p.65** | *µmol g DW^-1^* | 1.71 ± 0.49^ab^ | 1.92 ± 0.22^ab^ | 1.74 ± 0.09^ab^ | 2.38 ± 0.27^a^ | 1.95 ± 0.26^ab^ | 1.40 ± 0.19^ab^ | 0.94 ± 0.01^b^ | 1.51 ± 0.10^ab^ | 1.45 ± 0.10^ab^ | 1.28 ± 0.07^ab^ | 0.317 | 0.000 | 0.342 |
| **Glc6P.a.65** | *µmol g DW^-1^* | 2.09 ± 0.30 | 1.52 ± 0.19 | 1.74 ± 0.26 | 1.92 ± 0.13 | 1.71 ± 0.08 | 1.50 ± 0.06 | 1.53 ± 0.07 | 1.66 ± 0.08 | 1.47 ± 0.21 | 1.53 ± 0.05 | 0.592 | 0.025 | 0.365 |
| **Glc6P.g.65** | *µmol g DW^-1^* | 2.49 ± 0.31^a^ | 1.74 ± 0.28^ac^ | 2.04 ± 0.28^ac^ | 2.34 ± 0.12^ab^ | 1.83 ± 0.20^ac^ | 1.53 ± 0.08^bc^ | 1.49 ± 0.06^bc^ | 1.70 ± 0.06^ac^ | 1.60 ± 0.06^ac^ | 1.18 ± 0.09^c^ | 0.045 | 0.000 | 0.309 |
| **Glc6P.l.65** | *µmol g DW^-1^* | 3.45 ± 0.34^a^ | 2.36 ± 0.44^bc^ | 2.17 ± 0.12^bc^ | 2.85 ± 0.27^ab^ | 2.51 ± 0.02^ac^ | 1.82 ± 0.06^bc^ | 1.80 ± 0.08^bc^ | 2.04 ± 0.12^bc^ | 1.78 ± 0.04^c^ | 2.02 ± 0.14^bc^ | 0.099 | 0.000 | 0.017 |
| **Glc6P.b.75** | *µmol g DW^-1^* | 1.45 ± 0.07^ab^ | 1.46 ± 0.16^ab^ | 1.44 ± 0.10^ab^ | 1.72 ± 0.22^a^ | 1.44 ± 0.15^ab^ | 0.94 ± 0.03^b^ | 1.12 ± 0.09^ab^ | 0.93 ± 0.04^b^ | 1.14 ± 0.07^ab^ | 1.06 ± 0.21^ab^ | 0.372 | 0.000 | 0.890 |
| **Glc6P.s.75** | *µmol g DW^-1^* | 0.74 ± 0.06^ab^ | 0.70 ± 0.03^ab^ | 0.74 ± 0.08^ab^ | 0.74 ± 0.08^ab^ | 0.63 ± 0.03^ab^ | 0.59 ± 0.02^b^ | 0.56 ± 0.02^b^ | 0.59 ± 0.03^b^ | 0.66 ± 0.04^ab^ | 0.83 ± 0.02^a^ | 0.324 | 0.045 | 0.005 |
| **Glc6P.p.75** | *µmol g DW^-1^* | 0.56 ± 0.02^bc^ | 0.58 ± 0.04^bc^ | 0.57 ± 0.08^bc^ | 0.53 ± 0.01^c^ | 0.56 ± 0.06^bc^ | 0.75 ± 0.04^b^ | 0.62 ± 0.02^bc^ | 0.61 ± 0.03^bc^ | 0.68 ± 0.06^bc^ | 0.98 ± 0.04^a^ | 0.003 | 0.000 | 0.002 |
| **Glc6P.a.75** | *µmol g DW^-1^* | 1.20 ± 0.13 | 1.14 ± 0.20 | 0.83 ± 0.06 | 1.20 ± 0.19 | 1.29 ± 0.20 | 1.10 ± 0.08 | 1.08 ± 0.11 | 0.89 ± 0.11 | 1.30 ± 0.30 | 1.47 ± 0.36 | 0.142 | 0.795 | 0.948 |
| **Glc6P.g.75** | *µmol g DW^-1^* | 1.44 ± 0.18 | 1.27 ± 0.14 | 1.20 ± 0.06 | 1.53 ± 0.17 | 1.00 ± 0.08 | 1.64 ± 0.14 | 1.39 ± 0.14 | 1.19 ± 0.11 | 1.90 ± 0.34 | 1.50 ± 0.30 | 0.063 | 0.059 | 0.684 |
| **Glc6P.l.75** | *µmol g DW^-1^* | 1.53 ± 0.21^ab^ | 1.10 ± 0.10^ab^ | 0.97 ± 0.04^b^ | 1.29 ± 0.15^ab^ | 1.11 ± 0.12^ab^ | 1.45 ± 0.10^ab^ | 1.72 ± 0.13^ab^ | 1.13 ± 0.08^ab^ | 2.09 ± 0.51^a^ | 1.63 ± 0.30^ab^ | 0.100 | 0.009 | 0.296 |
| **Fru.b.65** | *µmol g DW^-1^* | 21.4 ± 1.6^ac^ | 10.5 ± 3.3^c^ | 13.4 ± 4.9^bc^ | 15.9 ± 0.6^bc^ | 36.0 ± 11.9^ac^ | 45.6 ± 7.3^a^ | 10.6 ± 2.2^bc^ | 21.6 ± 4.8^ac^ | 39.5 ± 8.1^ab^ | 14.7 ± 2.7^bc^ | 0.007 | 0.072 | 0.004 |
| **Fru.s.65** | *µmol g DW^-1^* | 29.5 ± 1.7^ac^ | 23.8 ± 5.0^ac^ | 17.9 ± 7.6^bc^ | 24.5 ± 1.6^ac^ | 42.9 ± 7.0^a^ | 33.4 ± 5.4^ab^ | 10.1 ± 1.6^c^ | 22.4 ± 4.2^ac^ | 27.4 ± 3.1^ac^ | 23.6 ± 3.9^ac^ | 0.008 | 0.152 | 0.041 |
| **Fru.p.65** | *µmol g DW^-1^* | 120.0 ± 36.7^ab^ | 132.0 ± 16.9^ab^ | 125.0 ± 16.2^ab^ | 179.0 ± 19.5^a^ | 168.0 ± 16.3^a^ | 133.0 ± 22.1^ab^ | 46.3 ± 3.5^b^ | 157.0 ± 10.9^a^ | 128.0 ± 10.9^ab^ | 93.9 ± 6.1^ab^ | 0.025 | 0.009 | 0.012 |
| **Fru.a.65** | *µmol g DW^-1^* | 13.7 ± 2.4 | 9.9 ± 3.9 | 7.8 ± 0.6 | 11.4 ± 1.7 | 14.1 ± 2.7 | 18.2 ± 5.3 | 10.2 ± 1.5 | 13.9 ± 4.9 | 17.1 ± 1.8 | 11.6 ± 3.4 | 0.343 | 0.171 | 0.596 |
| **Fru.g.65** | *µmol g DW^-1^* | 28.8 ± 3.7 | 28.9 ± 7.7 | 20.9 ± 0.9 | 27.3 ± 1.5 | 25.2 ± 3.3 | 31.0 ± 2.6 | 30.4 ± 3.9 | 24.0 ± 0.3 | 35.4 ± 3.3 | 30.0 ± 11.1 | 0.433 | 0.219 | 0.964 |
| **Fru.l.65** | *µmol g DW^-1^* | 77.3 ± 10.3^ab^ | 52.8 ± 3.4^ac^ | 38.9 ± 4.6^c^ | 62.7 ± 7.4^ac^ | 78.9 ± 12.6^a^ | 55.9 ± 3.7^ac^ | 35.1 ± 2.4^c^ | 43.3 ± 5.2^bc^ | 54.7 ± 3.5^ac^ | 58.8 ± 8.1^ac^ | 0.001 | 0.009 | 0.331 |
| **Fru.b.75** | *µmol g DW^-1^* | 49.9 ± 17.2^ac^ | 27.8 ± 7.5^bc^ | 29.0 ± 8.0^bc^ | 28.8 ± 13.3^bc^ | 98.7 ± 6.8^a^ | 89.6 ± 23.0^ab^ | 30.0 ± 5.8^bc^ | 47.7 ± 21.1^ac^ | 74.8 ± 5.2^ac^ | 15.9 ± 0.6^c^ | 0.041 | 0.566 | 0.000 |
| **Fru.s.75** | *µmol g DW^-1^* | 44.4 ± 11.1^ab^ | 40.9 ± 18.7^ab^ | 31.9 ± 15.3^b^ | 27.0 ± 6.8^b^ | 93.2 ± 15.1^a^ | 38.3 ± 4.4^b^ | 29.0 ± 3.13^b^ | 25.5 ± 7.9^b^ | 24.7 ± 3.8^b^ | 48.1 ± 9.9^ab^ | 0.004 | 0.050 | 0.305 |
| **Fru.p.75** | *µmol g DW^-1^* | 50.2 ± 13.0 | 35.1 ± 8.6 | 29.9 ± 9.1 | 30.2 ± 13.4 | 66.1 ± 17.9 | 32.0 ± 8.7 | 22.9 ± 6.6 | 20.7 ± 3.6 | 23.8 ± 2.3 | 56.7 ± 11.9 | 0.013 | 0.111 | 0.984 |
| **Fru.a.75** | *µmol g DW^-1^* | 15.5 ± 4.5^b^ | 12.3 ± 6.4^b^ | 7.4 ± 1.1^b^ | 8.3 ± 2.6^b^ | 43.1 ± 3.3^a^ | 14.9 ± 2.7^b^ | 12.3 ± 3.1^b^ | 12.8 ± 2.2^b^ | 14.6 ± 3.8^b^ | 29.7 ± 9.7^ab^ | 0.000 | 0.878 | 0.249 |
| **Fru.g.75** | *µmol g DW^-1^* | 21.9 ± 7.7^ab^ | 28.6 ± 9.2^ab^ | 21.5 ± 5.2^ab^ | 20.4 ± 3.8^b^ | 51.3 ± 5.7^a^ | 23 ± 3.5^ab^ | 20.6 ± 3.4^b^ | 17.6 ± 5.0^b^ | 13.6 ± 1.9^b^ | 37.7 ± 9.9^ab^ | 0.002 | 0.118 | 0.807 |
| **Fru.l.75** | *µmol g DW^-1^* | 33.8 ± 10.1^ab^ | 28.9 ± 8.7^ab^ | 18.7 ± 2.4^b^ | 18.5 ± 3.3^b^ | 57.8 ± 5.4^a^ | 30.3 ± 2.0^ab^ | 26.6 ± 3.6^ab^ | 20.6 ± 5.0^b^ | 27.5 ± 6.5^ab^ | 42.1 ± 12.1^ab^ | 0.002 | 0.630 | 0.487 |
| **Suc.b.65** | *µmol g DW^-1^* | 115 ± 13^d^ | 171 ± 29^ad^ | 193 ± 21^ad^ | 137 ± 12^cd^ | 231 ± 24^ac^ | 163 ± 18^bcd^ | 265 ± 5^a^ | 226 ± 37^ac^ | 208 ± 6^ad^ | 254 ± 5^ab^ | 0.000 | 0.000 | 0.408 |
| **Suc.s.65** | *µmol g DW^-1^* | 139 ± 8^e^ | 180 ± 19^bce^ | 167 ± 3^ce^ | 142 ± 10^de^ | 225 ± 7^ab^ | 183 ± 18^bce^ | 219 ± 3^ac^ | 193 ± 11^bcd^ | 208 ± 3^ac^ | 245 ± 2^a^ | 0.000 | 0.000 | 0.241 |
| **Suc.p.65** | *µmol g DW^-1^* | 90 ± 27^c^ | 175 ± 44^ac^ | 196 ± 20^ab^ | 115 ± 22^bc^ | 199 ± 7^ab^ | 140 ± 9^bc^ | 265 ± 6^a^ | 166 ± 24^ac^ | 176 ± 13^ac^ | 263 ± 1^a^ | 0.000 | 0.002 | 0.093 |
| **Suc.a.65** | *µmol g DW^-1^* | 84 ± 10^d^ | 134 ± 20^bcd^ | 139 ± 21^bcd^ | 86 ± 10^d^ | 127 ± 2^cd^ | 116 ± 10^cd^ | 233 ± 14^a^ | 188 ± 17^ac^ | 130 ± 24^bcd^ | 212 ± 25^ab^ | 0.000 | 0.000 | 0.245 |
| **Suc.g.65** | *µmol g DW^-1^* | 102 ± 5^de^ | 130 ± 38^ce^ | 169 ± 16^ace^ | 90 ± 6^e^ | 185 ± 8^acd^ | 153 ± 11^bce^ | 238 ± 24^ab^ | 204 ± 9^ac^ | 152 ± 13^ce^ | 244 ± 14^a^ | 0.000 | 0.000 | 0.321 |
| **Suc.l.65** | *µmol g DW^-1^* | 132 ± 6^cd^ | 158 ± 50^cd^ | 148 ± 10^cd^ | 112 ± 12^d^ | 173 ± 5^bcd^ | 173 ± 16^bcd^ | 286 ± 18^ab^ | 237 ± 26^ac^ | 165 ± 17^bcd^ | 316 ± 42^a^ | 0.002 | 0.000 | 0.205 |


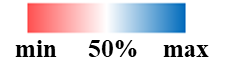
**Supplementary Table S1**. Continued.

| **Variable** | **Units** | **Irrigated** | | | | | **Rainfed** | | | | | ***P*-value** | | |
| --- | --- | --- | --- | --- | --- | --- | --- | --- | --- | --- | --- | --- | --- | --- |
|  |  | **MEX** | **EUR** | **DRI** | **KNI** | **HAR** | **MEX** | **EUR** | **DRI** | **KNI** | **HAR** | **G** | **W** | **G×W** |
| **Suc.b.75** | *µmol g DW^-1^* | 149 ± 29 | 172 ± 41 | 175 ± 20 | 148 ± 12 | 153 ± 21 | 162 ± 6 | 239 ± 9 | 200 ± 16 | 231 ± 29 | 193 ± 34 | 0.341 | 0.008 | 0.583 |
| **Suc.s.75** | *µmol g DW^-1^* | 193 ± 23 | 189 ± 29 | 178 ± 15 | 200 ± 4 | 170 ± 5 | 192 ± 10 | 204 ± 8 | 197 ± 12 | 195 ± 13 | 195 ± 7 | 0.821 | 0.277 | 0.804 |
| **Suc.p.75** | *µmol g DW^-1^* | 253 ± 13 | 239 ± 25 | 253 ± 13 | 274 ± 17 | 226 ± 19 | 254 ± 3 | 287 ± 10 | 298 ± 13 | 272 ± 5 | 261 ± 18 | 0.213 | 0.014 | 0.297 |
| **Suc.a.75** | *µmol g DW^-1^* | 89.1 ± 9.3^ab^ | 93.1 ± 24.8^ab^ | 56.2 ± 3.9^b^ | 68.2 ± 7.7^ab^ | 97.1 ± 5.1^ab^ | 144.0 ± 13.0^ab^ | 155.0 ± 10.7^ab^ | 149.0 ± 18.6^ab^ | 186.0 ± 49.5^a^ | 154.0 ± 47.9^ab^ | 0.857 | 0.000 | 0.663 |
| **Suc.g.75** | *µmol g DW^-1^* | 104 ± 10^bc^ | 121 ± 28^ac^ | 84 ± 6^c^ | 106 ± 17^bc^ | 153 ± 17^ac^ | 180 ± 16^ab^ | 209 ± 7^a^ | 151 ± 20^ac^ | 186 ± 26^ab^ | 171 ± 27^ac^ | 0.128 | 0.000 | 0.396 |
| **Suc.l.75** | *µmol g DW^-1^* | 133 ± 11^ac^ | 121 ± 22^ac^ | 78 ± 3^c^ | 88 ± 17^bc^ | 140 ± 12^ac^ | 196 ± 35^ac^ | 232 ± 15^ab^ | 153 ± 10^ac^ | 263 ± 66^a^ | 178 ± 40^ac^ | 0.255 | 0.000 | 0.200 |
| **starch.b.65** | *µmol g DW^-1^* | 12.8 ± 2.0^b^ | 16.1 ± 3.8^b^ | 25.7 ± 4.2^ab^ | 19.2 ± 1.6^b^ | 28.5 ± 16.0^ab^ | 7.2 ± 3.4^b^ | 54.0 ± 4.3^a^ | 23.0 ± 5.2^ab^ | 7.8 ± 2.7^b^ | 22.3 ± 5.3^b^ | 0.005 | 0.556 | 0.005 |
| **starch.s.65** | *µmol g DW^-1^* | 4.08 ± 0.10^c^ | 5.48 ± 2.17^c^ | 7.25 ± 1.73^c^ | 4.42 ± 0.47^c^ | 8.35 ± 3.81^bc^ | 3.64 ± 1.21^c^ | 17.70 ± 2.61^ab^ | 6.43 ± 0.84^c^ | 5.34 ± 1.43^c^ | 19.20 ± 3.17^a^ | 0.000 | 0.003 | 0.007 |
| **starch.p.65** | *µmol g DW^-1^* | 6.07 ± 1.32^c^ | 9.50 ± 2.69^bc^ | 8.49 ± 1.18^bc^ | 10.20 ± 2.81^bc^ | 9.05 ± 1.67^bc^ | 5.32 ± 0.52^c^ | 17.30 ± 0.53^ab^ | 9.38 ± 0.79^bc^ | 7.62 ± 2.83^bc^ | 25.50 ± 2.93^a^ | 0.000 | 0.002 | 0.001 |
| **starch.a.65** | *µmol g DW^-1^* | 13.1 ± 2.4^c^ | 22.3 ± 6.7^bc^ | 21.5 ± 4.4^bc^ | 13.4 ± 2.0^c^ | 20.5 ± 6.0^bc^ | 15.8 ± 2.8^bc^ | 47.1 ± 3.8^a^ | 29.9 ± 0.6^ac^ | 18.0 ± 3.8^bc^ | 38.8 ± 9.8^ab^ | 0.002 | 0.001 | 0.157 |
| **starch.g.65** | *µmol g DW^-1^* | 11.3 ± 0.5^ab^ | 12 ± 5.2^ab^ | 10.3 ± 1.5^b^ | 10.1 ± 1.3^b^ | 13.4 ± 3.5^ab^ | 12.5 ± 2.0^ab^ | 18.8 ± 3.0^ab^ | 16.4 ± 2.2^ab^ | 13.0 ± 2.7^ab^ | 24.5 ± 2.3^a^ | 0.077 | 0.004 | 0.433 |
| **starch.l.65** | *µmol g DW^-1^* | 35.3 ± 4.5^b^ | 28.9 ± 11.4^b^ | 30.6 ± 3.3^b^ | 22.7 ± 2.4^b^ | 38.3 ± 3.3^b^ | 29.7 ± 3.4^b^ | 52.5 ± 10.6^ab^ | 44.1 ± 5.1^b^ | 25.3 ± 5.6^b^ | 80.2 ± 11.6^a^ | 0.001 | 0.003 | 0.026 |
| **starch.b.75** | *µmol g DW^-1^* | 11.9 ± 7.7 | 14.4 ± 7.2 | 13.2 ± 6.5 | 15.3 ± 8.2 | 4.6 ± 1.6 | 4.96 ± 1.3 | 14.1 ± 1.1 | 10.2 ± 2.6 | 8.41 ± 1.7 | 23.2 ± 2.1 | 0.777 | 0.923 | 0.091 |
| **starch.s.75** | *µmol g DW^-1^* | 2.35 ± 1.3^ab^ | 4.15 ± 2.4^ab^ | 3.14 ± 0.8^ab^ | 2.76 ± 1.1^ab^ | 1.38 ± 0.3^b^ | 3.38 ± 0.79^ab^ | 5.42 ± 0.52^ab^ | 4.47 ± 1.12^ab^ | 5.40 ± 0.43^ab^ | 7.56 ± 0.80^a^ | 0.481 | 0.002 | 0.144 |
| **starch.p.75** | *µmol g DW^-1^* | 1.08 ± 0.42 | 1.32 ± 0.62 | 0.978 ± 0.37 | 1.59 ± 0.24 | 1.02 ± 0.24 | 1.82 ± 0.09 | 2.43 ± 0.39 | 2.84 ± 0.56 | 2.26 ± 0.44 | 2.36 ± 0.76 | 0.805 | 0.001 | 0.682 |
| **starch.a.75** | *µmol g DW^-1^* | 10.5 ± 3.4^ab^ | 10.5 ± 6.0^ab^ | 7.3 ± 1.7^b^ | 6.8 ± 0.8^b^ | 6.7 ± 0.9^b^ | 20.2 ± 3.7^ab^ | 21.2 ± 3.0^ab^ | 21.1 ± 3.1^ab^ | 27.7 ± 7.3^a^ | 13.4 ± 3.4^ab^ | 0.422 | 0.000 | 0.434 |
| **starch.g.75** | *µmol g DW^-1^* | 6.6 ± 1.6^bc^ | 9.0 ± 3.8^ac^ | 4.2 ± 1.3^c^ | 7.9 ± 1.7^ac^ | 7.9 ± 1.3^ac^ | 16.1 ± 1.8^ab^ | 17.9 ± 1.9^a^ | 12.1 ± 2.4^ac^ | 17.3 ± 2.5^a^ | 14.0 ± 1.2^ac^ | 0.157 | 0.000 | 0.919 |
| **starch.l.75** | *µmol g DW^-1^* | 14.7 ± 4.2^bc^ | 17.9 ± 6.0^bc^ | 8.8 ± 1.4^c^ | 8.4 ± 0.8^c^ | 9.2 ± 1.3^bc^ | 26.0 ± 4.0^ac^ | 42.9 ± 8.2^a^ | 19.0 ± 1.0^ac^ | 33.5 ± 9.6^ab^ | 18.9 ± 3.2^ac^ | 0.020 | 0.000 | 0.292 |
| **malate.b.65** | *µmol g DW^-1^* | 14.7 ± 1.0^a^ | 6.6 ± 1.2^c^ | 10.6 ± 1.0^ac^ | 12.3 ± 0.1^ab^ | 7.9 ± 1.0^bc^ | 12.3 ± 0.5^ab^ | 6.8 ± 0.7^c^ | 9.2 ± 0.6^bc^ | 11.2 ± 2.4^ac^ | 7.2 ± 0.3^bc^ | 0.000 | 0.120 | 0.795 |
| **malate.s.65** | *µmol g DW^-1^* | 12.5 ± 1.3^a^ | 8.7 ± 0.4^bd^ | 8.9 ± 0.4^bc^ | 10.8 ± 0.7^ab^ | 8.4 ± 0.2^bd^ | 9.3 ± 0.3^bc^ | 5.9 ± 0.6^d^ | 7.0 ± 0.4^cd^ | 9.5 ± 0.0^bc^ | 5.9 ± 0.6^d^ | 0.000 | 0.000 | 0.526 |
| **malate.p.65** | *µmol g DW^-1^* | 5.77 ± 1.66^ab^ | 5.12 ± 1.05^ab^ | 4.91 ± 0.55^ab^ | 8.33 ± 1.31^a^ | 7.85 ± 1.59^a^ | 4.93 ± 1.20^ab^ | 1.29 ± 0.41^b^ | 4.94 ± 0.79^ab^ | 4.35 ± 0.36^ab^ | 3.38 ± 0.25^ab^ | 0.071 | 0.001 | 0.140 |
| **malate.a.65** | *µmol g DW^-1^* | 3.09 ± 0.69^a^ | 1.01 ± 0.25^b^ | 1.27 ± 0.27^b^ | 3.33 ± 0.12^a^ | 2.61 ± 0.35^ab^ | 2.02 ± 0.35^ab^ | 1.24 ± 0.08^b^ | 1.98 ± 0.21^ab^ | 1.85 ± 0.30^ab^ | 1.47 ± 0.17^b^ | 0.001 | 0.013 | 0.009 |
| **malate.g.65** | *µmol g DW^-1^* | 1.28 ± 0.43 | 0.62 ± 0.11 | 0.76 ± 0.13 | 1.65 ± 0.21 | 1.55 ± 0.30 | 2.49 ± 1.11 | 1.96 ± 0.15 | 1.47 ± 0.23 | 2.31 ± 0.48 | 1.35 ± 0.25 | 0.257 | 0.015 | 0.460 |
| **malate.l.65** | *µmol g DW^-1^* | 4.87 ± 0.52^a^ | 1.95 ± 0.49^b^ | 1.90 ± 0.13^b^ | 4.83 ± 0.54^a^ | 4.31 ± 0.46^ab^ | 3.35 ± 0.96^ab^ | 2.01 ± 0.04^b^ | 2.56 ± 0.58^ab^ | 3.70 ± 0.67^ab^ | 3.10 ± 0.47^ab^ | 0.001 | 0.081 | 0.239 |
| **malate.b.75** | *µmol g DW^-1^* | 12.00 ± 0.81^a^ | 6.18 ± 0.39^b^ | 9.23 ± 0.77^ab^ | 12.00 ± 1.94^a^ | 10.20 ± 0.79^ab^ | 8.97 ± 1.35^ab^ | 6.07 ± 0.31^b^ | 7.63 ± 1.13^ab^ | 11.00 ± 1.38^ab^ | 7.81 ± 1.01^ab^ | 0.001 | 0.030 | 0.709 |
| **malate.s.75** | *µmol g DW^-1^* | 9.17 ± 0.56^ab^ | 7.85 ± 0.77^ac^ | 7.46 ± 0.36^ac^ | 9.29 ± 0.40^a^ | 6.55 ± 0.34^bc^ | 6.46 ± 0.58^c^ | 5.87 ± 0.56^c^ | 6.01 ± 0.77^c^ | 6.75 ± 0.23^ac^ | 6.07 ± 0.51^c^ | 0.020 | 0.000 | 0.263 |
| **malate.p.75** | *µmol g DW^-1^* | 0.955 ± 0.092^ac^ | 0.674 ± 0.143^ac^ | 1.010 ± 0.25^ab^ | 0.566 ± 0.053^ac^ | 0.477 ± 0.116^bc^ | 0.926 ± 0.042^ac^ | 0.429 ± 0.009^c^ | 0.972 ± 0.140^ac^ | 0.564 ± 0.054^ac^ | 1.050 ± 0.014^a^ | 0.002 | 0.482 | 0.023 |
| **malate.a.75** | *µmol g DW^-1^* | 2.10 ± 0.22 | 1.77 ± 0.28 | 1.24 ± 0.06 | 2.82 ± 0.26 | 2.88 ± 0.23 | 0.72 ± 0.10 | 0.94 ± 0.12 | 0.62 ± 0.09 | 1.56 ± 0.50 | 2.35 ± 1.23 | 0.009 | 0.004 | 0.842 |
| **malate.g.75** | *µmol g DW^-1^* | 1.12 ± 0.23^ac^ | 0.90 ± 0.28^bc^ | 0.77 ± 0.10^c^ | 2.01 ± 0.25^a^ | 1.14 ± 0.07^ac^ | 1.33 ± 0.17^ac^ | 1.43 ± 0.07^ac^ | 0.94 ± 0.08^bc^ | 1.73 ± 0.18^ab^ | 0.91 ± 0.21^bc^ | 0.000 | 0.491 | 0.176 |
| **malate.l.75** | *µmol g DW^-1^* | 1.86 ± 0.30 | 0.83 ± 0.22 | 0.96 ± 0.04 | 2.18 ± 0.22 | 2.18 ± 0.12 | 1.29 ± 0.14 | 1.46 ± 0.05 | 1.24 ± 0.02 | 2.37 ± 0.47 | 1.92 ± 0.85 | 0.007 | 0.801 | 0.459 |
| **Glu.b.65** | *µmol g DW^-1^* | 23.7 ± 3.5^ac^ | 26.2 ± 2.5^ab^ | 31.0 ± 3.5^a^ | 28.2 ± 0.3^ab^ | 23.1 ± 1.0^ac^ | 18.1 ± 3.1^bc^ | 18.1 ± 0.5^bc^ | 21.2 ± 1.8^ac^ | 18.9 ± 2.6^bc^ | 14.0 ± 1.6^c^ | 0.044 | 0.000 | 0.902 |
| **Glu.s.65** | *µmol g DW^-1^* | 13.6 ± 1.5^ab^ | 13.9 ± 0.2^ab^ | 16.2 ± 0.4^a^ | 13.4 ± 0.6^ab^ | 12.8 ± 0.6^ac^ | 11.3 ± 0.1^bc^ | 9.5 ± 0.9^c^ | 10.6 ± 0.6^bc^ | 12.5 ± 0.8^bc^ | 9.8 ± 0.4^c^ | 0.049 | 0.000 | 0.034 |
| **Glu.p.65** | *µmol g DW^-1^* | 8.6 ± 2.7 | 13.8 ± 0.8 | 13.8 ± 2.0 | 13.1 ± 1.9 | 12.3 ± 0.9 | 10.2 ± 1.1 | 12.9 ± 1.0 | 13.0 ± 0.4 | 12.1 ± 0.3 | 12.3 ± 0.5 | 0.050 | 0.772 | 0.875 |
| **Glu.a.65** | *µmol g DW^-1^* | 8.72 ± 1.64 | 8.46 ± 0.50 | 8.94 ± 0.96 | 10.40 ± 1.19 | 8.14 ± 0.45 | 6.35 ± 0.39 | 9.22 ± 0.95 | 10.60 ± 1.02 | 7.60 ± 1.42 | 6.35 ± 0.64 | 0.100 | 0.172 | 0.135 |
| **Glu.g.65** | *µmol g DW^-1^* | 9.5 ± 1.0 | 11.2 ± 2.9 | 11.7 ± 1.7 | 12.1 ± 0.6 | 13.2 ± 0.6 | 11.3 ± 0.9 | 15.3 ± 1.2 | 11.4 ± 0.5 | 12.9 ± 1.6 | 13.2 ± 1.0 | 0.234 | 0.158 | 0.529 |
| **Glu.l.65** | *µmol g DW^-1^* | 15.0 ± 1.8 | 14.0 ± 3.7 | 13.4 ± 1.3 | 14.8 ± 1.4 | 15.5 ± 0.8 | 11.6 ± 0.5 | 17.2 ± 1.5 | 13.6 ± 2.7 | 14.2 ± 2.2 | 14.8 ± 1.0 | 0.711 | 0.844 | 0.569 |
| **Glu.b.75** | *µmol g DW^-1^* | 19.7 ± 1.8^ab^ | 20.5 ± 1.2^ab^ | 24.1 ± 0.9^a^ | 22.1 ± 1.1^ab^ | 16.3 ± 1.6^bc^ | 11.8 ± 0.7^c^ | 11.6 ± 0.7^c^ | 10.1 ± 0.6^c^ | 12.2 ± 1.9^c^ | 12.0 ± 1.5^c^ | 0.158 | 0.000 | 0.020 |
| **Glu.s.75** | *µmol g DW^-1^* | 9.71 ± 0.81 | 9.44 ± 2.36 | 9.63 ± 0.51 | 10.9 ± 0.64 | 8.02 ± 1.41 | 7.42 ± 0.35 | 6.76 ± 0.22 | 6.35 ± 0.13 | 7.84 ± 0.28 | 7.73 ± 0.16 | 0.543 | 0.001 | 0.556 |
| **Glu.p.75** | *µmol g DW^-1^* | 7.78 ± 0.59 | 10.40 ± 2.11 | 7.79 ± 0.95 | 9.32 ± 0.38 | 5.59 ± 0.40 | 9.89 ± 0.73 | 9.91 ± 0.67 | 9.56 ± 1.33 | 8.46 ± 0.79 | 6.52 ± 0.12 | 0.007 | 0.274 | 0.462 |
| **Glu.a.75** | *µmol g DW^-1^* | 6.68 ± 0.71 | 8.50 ± 2.63 | 5.50 ± 0.04 | 9.20 ± 0.58 | 5.89 ± 1.15 | 5.27 ± 0.44 | 7.53 ± 0.58 | 6.31 ± 0.88 | 10.10 ± 2.33 | 7.03 ± 2.18 | 0.074 | 0.923 | 0.846 |
| **Glu.g.75** | *µmol g DW^-1^* | 9.4 ± 0.6^b^ | 9.0 ± 1.6^b^ | 8.2 ± 0.5^b^ | 12.8 ± 1.1^ab^ | 10.3 ± 0.7^ab^ | 13.2 ± 1.1^ab^ | 14.6 ± 0.1^ab^ | 9.2 ± 0.3^b^ | 16.7 ± 3.1^a^ | 10.9 ± 1.9^ab^ | 0.006 | 0.003 | 0.345 |
| **Glu.l.75** | *µmol g DW^-1^* | 11.5 ± 1.2^ab^ | 8.96 ± 2.0^ab^ | 7.7 ± 0.9^b^ | 11.0 ± 0.4^ab^ | 10.4 ± 1.9^ab^ | 12.2 ± 0.9^ab^ | 15.6 ± 1.2^ab^ | 9.1 ± 0.7^ab^ | 18.4 ± 4.6^a^ | 11.2 ± 3.0^ab^ | 0.078 | 0.018 | 0.297 |
| **aa.b.65** | *µmol g DW^-1^* | 62.0 ± 7.6 | 67.6 ± 6.3 | 82.4 ± 4.1 | 76.4 ± 1.0 | 69.2 ± 8.2 | 71.2 ± 7.2 | 60.6 ± 5.4 | 69.6 ± 4.2 | 64.8 ± 5.3 | 67.1 ± 1.1 | 0.298 | 0.183 | 0.316 |
| **aa.s.65** | *µmol g DW^-1^* | 43.8 ± 0.4 | 38.4 ± 4.9 | 48.6 ± 2.3 | 40.5 ± 4.9 | 41.7 ± 2.5 | 46.6 ± 4.7 | 44.0 ± 5.2 | 39.3 ± 3.4 | 56.5 ± 4.4 | 51.2 ± 4.9 | 0.465 | 0.067 | 0.058 |
| **aa.p.65** | *µmol g DW^-1^* | 26.9 ± 8.0^d^ | 40.9 ± 6.4^cd^ | 47.8 ± 7.9^bcd^ | 36.9 ± 5.7^cd^ | 57 ± 7.7^ac^ | 38.4 ± 1.0^cd^ | 76.5 ± 5.4^ab^ | 52.1 ± 0.4^ad^ | 53.1 ± 1.6^ad^ | 77.5 ± 6.7^a^ | 0.000 | 0.000 | 0.128 |
| **aa.a.65** | *µmol g DW^-1^* | 35.8 ± 6.4 | 29.5 ± 2.6 | 33.0 ± 6.6 | 34.6 ± 1.8 | 33.6 ± 4.6 | 28.8 ± 5.0 | 49.2 ± 6.1 | 32.3 ± 2.4 | 38.5 ± 7.0 | 34.8 ± 5.5 | 0.618 | 0.303 | 0.151 |
| **aa.g.65** | *µmol g DW^-1^* | 32.8 ± 3.4^d^ | 53.7 ± 12.5^cd^ | 47.9 ± 6.4^cd^ | 48.6 ± 5.1^cd^ | 76.3 ± 9.1^ac^ | 59.1 ± 8.9^bcd^ | 97.4 ± 9.9^ab^ | 56.7 ± 0.9^cd^ | 83.1 ± 11.2^ac^ | 109.0 ± 2.8^a^ | 0.000 | 0.000 | 0.289 |
| **aa.l.65** | *µmol g DW^-1^* | 47.6 ± 7.3^c^ | 44.0 ± 15.3^c^ | 43.2 ± 3.0^c^ | 45.3 ± 7.6^c^ | 73.2 ± 8.3^ac^ | 63.3 ± 8.3^bc^ | 110.0 ± 6.6^a^ | 48.1 ± 5.9^c^ | 80.4 ± 7.6^ac^ | 92.1 ± 9.3^ab^ | 0.002 | 0.000 | 0.016 |


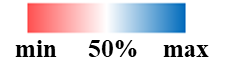
**Supplementary Table S1**. Continued.

| **Variable** | **Units** | **Irrigated** | | | | | **Rainfed** | | | | | ***P*-value** | | |
| --- | --- | --- | --- | --- | --- | --- | --- | --- | --- | --- | --- | --- | --- | --- |
|  |  | **MEX** | **EUR** | **DRI** | **KNI** | **HAR** | **MEX** | **EUR** | **DRI** | **KNI** | **HAR** | **G** | **W** | **G×W** |
| **aa.b.75** | *µmol g DW^-1^* | 48.6 ± 4.3^ab^ | 51.1 ± 4.1^ab^ | 51.6 ± 1.5^ab^ | 62.7 ± 0.8^a^ | 51.9 ± 6.1^ab^ | 59.7 ± 1.7^ab^ | 43.7 ± 6.2^ab^ | 55.5 ± 2.8^ab^ | 50.2 ± 5.78^ab^ | 41.2 ± 4.9^b^ | 0.120 | 0.262 | 0.051 |
| **aa.s.75** | *µmol g DW^-1^* | 32.0 ± 3.0^b^ | 27.0 ± 3.1^bc^ | 28.4 ± 2.1^bc^ | 31.3 ± 1.1^bc^ | 21.2 ± 1.5^c^ | 45.5 ± 2.0^a^ | 28.6 ± 1.2^bc^ | 30.9 ± 0.8^bc^ | 45.4 ± 3.1^a^ | 26.8 ± 1.2^bc^ | 0.000 | 0.000 | 0.014 |
| **aa.p.75** | *µmol g DW^-1^* | 29.7 ± 1.0^bc^ | 46.1 ± 2.1^a^ | 35.0 ± 3.3^ac^ | 43.7 ± 1.8^ab^ | 27.8 ± 3.2^c^ | 49.9 ± 3.3^a^ | 46.7 ± 4.9^a^ | 44.1 ± 4.3^ab^ | 41.8 ± 3.6^ac^ | 28.5 ± 2.3^bc^ | 0.000 | 0.010 | 0.014 |
| **aa.a.75** | *µmol g DW^-1^* | 35.1 ± 4.5 | 37.6 ± 9.6 | 22.1 ± 2.7 | 38.2 ± 4.4 | 27.4 ± 0.8 | 29.9 ± 1.1 | 38.3 ± 6.4 | 23.0 ± 3.8 | 44.8 ± 12.4 | 28.8 ± 7.4 | 0.053 | 0.827 | 0.926 |
| **aa.g.75** | *µmol g DW^-1^* | 40.8 ± 4.6^c^ | 34.6 ± 4.0^c^ | 31.4 ± 4.8^c^ | 81.5 ± 4.7^a^ | 30.1 ± 2.2^c^ | 73.7 ± 5.9^ab^ | 88.0 ± 6.3^a^ | 30.5 ± 0.91^c^ | 85.8 ± 13.9^a^ | 43.7 ± 7.5^bc^ | 0.000 | 0.000 | 0.002 |
| **aa.l.75** | *µmol g DW^-1^* | 54.7 ± 1.1^b^ | 38.9 ± 7.7^b^ | 30.3 ± 4.2^b^ | 66.5 ± 7.3^b^ | 42.5 ± 7.5^b^ | 82.2 ± 13.9^ab^ | 138.0 ± 14.5^a^ | 37.7 ± 0.3^b^ | 137.0 ± 23.1^a^ | 63.4 ± 19.8^b^ | 0.000 | 0.000 | 0.007 |
| **Prot.b.65** | *mg g DW^-1^* | 56.3 ± 5.7 | 66.0 ± 7.4 | 76.4 ± 7.0 | 64.6 ± 3.6 | 70.8 ± 3.9 | 62.4 ± 7.3 | 55.0 ± 2.8 | 66.7 ± 6.2 | 59.1 ± 4.6 | 59.3 ± 6.1 | 0.239 | 0.093 | 0.518 |
| **Prot.s.65** | *mg g DW^-1^* | 34.8 ± 4.9 | 32.0 ± 2.0 | 34.7 ± 1.0 | 34.1 ± 1.3 | 32.4 ± 1.2 | 33.3 ± 2.6 | 31.6 ± 1.1 | 30.7 ± 1.5 | 34.4 ± 1.4 | 33.3 ± 0.3 | 0.771 | 0.494 | 0.775 |
| **Prot.p.65** | *mg g DW^-1^* | 32.7 ± 8.3 | 34.8 ± 1.3 | 37.0 ± 2.1 | 45.6 ± 3.4 | 33.8 ± 1.7 | 30.6 ± 2.5 | 30.6 ± 0.4 | 36.9 ± 0.7 | 32.6 ± 0.9 | 32.2 ± 0.5 | 0.124 | 0.048 | 0.290 |
| **Prot.a.65** | *mg g DW^-1^* | 35.6 ± 4.9 | 29.3 ± 3.0 | 34 ± 6.6 | 35.4 ± 3.9 | 30.3 ± 1.7 | 30.6 ± 1.8 | 41 ± 2.1 | 39.7 ± 1.8 | 34.8 ± 4.5 | 31.5 ± 3.6 | 0.570 | 0.278 | 0.248 |
| **Prot.g.65** | *mg g DW^-1^* | 37.6 ± 3.6 | 27.5 ± 4.0 | 28.3 ± 3.0 | 31.9 ± 2.1 | 31.3 ± 1.3 | 30.1 ± 2.3 | 36 ± 2.3 | 29.9 ± 1.7 | 30.5 ± 1.5 | 31.7 ± 1.7 | 0.485 | 0.843 | 0.061 |
| **Prot.l.65** | *mg g DW^-1^* | 40 ± 4.3 | 30 ± 7.4 | 27 ± 1.4 | 33.9 ± 3.4 | 35.4 ± 1.7 | 29.9 ± 2.5 | 35.7 ± 1.5 | 31 ± 2.9 | 31.2 ± 2.0 | 38.7 ± 2.1 | 0.218 | 0.985 | 0.152 |
| **Prot.b.75** | *mg g DW^-1^* | 60.0 ± 3.4^ab^ | 55.3 ± 4.3^ac^ | 65.1 ± 0.9^a^ | 60.3 ± 2.9^a^ | 49.1 ± 3.3^ac^ | 40.3 ± 2.0^c^ | 43.7 ± 0.4^bc^ | 41.1 ± 4.1^c^ | 42.2 ± 3.7^c^ | 40.2 ± 4.8^c^ | 0.154 | 0.000 | 0.179 |
| **Prot.s.75** | *mg g DW^-1^* | 30.3 ± 2.6 | 27.9 ± 2.1 | 33.0 ± 1.2 | 30.4 ± 1.0 | 24.8 ± 2.8 | 29.0 ± 1.4 | 27.4 ± 0.3 | 29.9 ± 1.3 | 29.5 ± 1.4 | 30.9 ± 0.6 | 0.164 | 0.967 | 0.099 |
| **Prot.p.75** | *mg g DW^-1^* | 22.9 ± 1.2^ab^ | 24.8 ± 0.9^ab^ | 24.0 ± 1.1^ab^ | 24.0 ± 0.1^ab^ | 20.6 ± 1.4^b^ | 24.5 ± 2.2^ab^ | 27.6 ± 0.8^a^ | 28.0 ± 0.6^a^ | 25.3 ± 1.4^ab^ | 23.5 ± 1.3^ab^ | 0.015 | 0.004 | 0.815 |
| **Prot.a.75** | *mg g DW^-1^* | 22.0 ± 1.5 | 29.3 ± 7.8 | 18.8 ± 1.6 | 28.7 ± 2.1 | 24.3 ± 2.2 | 25.4 ± 0.72 | 33.0 ± 2.0 | 28.7 ± 3.9 | 39.7 ± 10.7 | 30.3 ± 9.6 | 0.267 | 0.064 | 0.933 |
| **Prot.g.75** | *mg g DW^-1^* | 24.7 ± 2.5 | 27.3 ± 5.3 | 18.8 ± 0.9 | 28.0 ± 3.3 | 24.5 ± 1.4 | 30.8 ± 1.8 | 35.2 ± 2.0 | 23.4 ± 1.9 | 35.0 ± 6.1 | 29.3 ± 5.5 | 0.052 | 0.014 | 0.989 |
| **Prot.l.75** | *mg g DW^-1^* | 25.8 ± 2.6 | 19.9 ± 2.2 | 17.9 ± 1.6 | 20.6 ± 1.2 | 23.2 ± 2.6 | 27.0 ± 4.6 | 33.6 ± 1.0 | 20.3 ± 0.8 | 35.8 ± 9.6 | 26.7 ± 4.5 | 0.220 | 0.009 | 0.263 |
| **Chla.b.65** | *mg g DW^-1^* | 9.13 ± 1.08^ab^ | 10.3 ± 0.43^ab^ | 9.28 ± 0.65^ab^ | 10.80 ± 0.96^a^ | 7.88 ± 0.45^ab^ | 7.35 ± 0.78^ab^ | 8.67 ± 0.40^ab^ | 10.20 ± 1.47^ab^ | 7.86 ± 0.44^ab^ | 6.55 ± 0.67^b^ | 0.026 | 0.015 | 0.223 |
| **Chla.s.65** | *mg g DW^-1^* | 5.57 ± 0.12^b^ | 6.79 ± 0.60^ab^ | 6.98 ± 0.49^ab^ | 5.86 ± 0.54^ab^ | 5.98 ± 0.75^ab^ | 5.35 ± 0.33^b^ | 9.05 ± 1.38^a^ | 7.07 ± 0.67^ab^ | 7.54 ± 0.27^ab^ | 8.75 ± 0.90^ab^ | 0.024 | 0.007 | 0.163 |
| **Chla.p.65** | *mg g DW^-1^* | 3.39 ± 0.74^b^ | 3.53 ± 0.38^ab^ | 4.17 ± 0.61^ab^ | 4.49 ± 0.07^ab^ | 2.92 ± 0.03^b^ | 3.62 ± 0.26^ab^ | 6.08 ± 0.95^a^ | 4.02 ± 0.33^ab^ | 3.61 ± 0.33^ab^ | 5.13 ± 0.79^ab^ | 0.242 | 0.031 | 0.016 |
| **Chla.a.65** | *mg g DW^-1^* | 5.51 ± 0.87 | 4.74 ± 0.58 | 5.45 ± 1.21 | 5.71 ± 0.64 | 3.95 ± 0.21 | 3.33 ± 0.15 | 5.85 ± 0.43 | 4.96 ± 0.46 | 4.16 ± 0.61 | 3.52 ± 0.75 | 0.148 | 0.104 | 0.167 |
| **Chla.g.65** | *mg g DW^-1^* | 3.96 ± 0.43 | 3.09 ± 0.37 | 3.71 ± 0.39 | 3.85 ± 0.13 | 3.2 ± 0.21 | 2.81 ± 0.18 | 3.67 ± 0.40 | 3.32 ± 0.18 | 2.69 ± 0.21 | 3.54 ± 0.29 | 0.952 | 0.075 | 0.021 |
| **Chla.l.65** | *mg g DW^-1^* | 4.85 ± 0.62 | 3.95 ± 1.04 | 3.79 ± 0.44 | 4.58 ± 0.27 | 3.82 ± 0.36 | 3.22 ± 0.18 | 4.66 ± 0.47 | 4.28 ± 0.45 | 3.08 ± 0.39 | 4.44 ± 0.70 | 0.935 | 0.456 | 0.084 |
| **Chla.b.75** | *mg g DW^-1^* | 7.41 ± 0.55 | 7.35 ± 0.57 | 9.11 ± 0.64 | 9.62 ± 1.09 | 8.72 ± 3.51 | 6.25 ± 1.44 | 7.85 ± 1.59 | 5.44 ± 1.21 | 4.33 ± 0.11 | 3.67 ± 0.59 | 0.896 | 0.004 | 0.234 |
| **Chla.s.75** | *mg g DW^-1^* | 5.82 ± 0.63 | 6.18 ± 0.69 | 6.1 ± 0.16 | 5.53 ± 0.12 | 7.57 ± 2.11 | 7.21 ± 1.34 | 7.09 ± 0.88 | 7.30 ± 2.37 | 7.53 ± 1.03 | 6.05 ± 1.21 | 0.999 | 0.333 | 0.687 |
| **Chla.p.75** | *mg g DW^-1^* | 5.08 ± 0.83 | 6.35 ± 1.16 | 5.11 ± 0.91 | 5.26 ± 0.59 | 5.48 ± 1.00 | 4.45 ± 0.55 | 6.22 ± 1.88 | 5.05 ± 1.13 | 5.77 ± 1.04 | 5.05 ± 0.53 | 0.654 | 0.820 | 0.985 |
| **Chla.a.75** | *mg g DW^-1^* | 3.01 ± 0.38 | 3.93 ± 0.89 | 3.08 ± 0.07 | 4.48 ± 0.64 | 2.70 ± 0.74 | 3.13 ± 0.17 | 3.61 ± 0.51 | 3.56 ± 0.78 | 3.37 ± 0.56 | 3.15 ± 1.17 | 0.511 | 0.861 | 0.743 |
| **Chla.g.75** | *mg g DW^-1^* | 2.52 ± 0.30 | 2.65 ± 0.45 | 2.05 ± 0.15 | 2.71 ± 0.10 | 2.41 ± 0.15 | 2.77 ± 0.29 | 3.53 ± 0.32 | 2.32 ± 0.35 | 2.42 ± 0.06 | 2.74 ± 0.51 | 0.097 | 0.146 | 0.471 |
| **Chla.l.75** | *mg g DW^-1^* | 2.74 ± 0.33 | 2.21 ± 0.28 | 2.07 ± 0.17 | 2.38 ± 0.19 | 2.52 ± 0.31 | 2.71 ± 0.34 | 3.61 ± 0.25 | 2.21 ± 0.31 | 2.98 ± 0.31 | 3.01 ± 1.14 | 0.500 | 0.082 | 0.561 |
| **Chlb.b.65** | *mg g DW^-1^* | 3.72 ± 0.35^b^ | 3.38 ± 0.20^b^ | 3.68 ± 0.27^b^ | 3.70 ± 0.16^b^ | 3.23 ± 0.18^b^ | 3.36 ± 0.11^b^ | 6.16 ± 0.46^a^ | 6.27 ± 1.15^a^ | 5.04 ± 0.27^ab^ | 3.26 ± 0.31^b^ | 0.003 | 0.000 | 0.005 |
| **Chlb.s.65** | *mg g DW^-1^* | 3.10 ± 0.17^b^ | 4.53 ± 0.41^b^ | 4.39 ± 0.18^b^ | 3.11 ± 0.18^b^ | 4.73 ± 1.41^b^ | 4.39 ± 0.62^b^ | 10.40 ± 1.67^a^ | 6.99 ± 1.31^ab^ | 7.97 ± 0.87^ab^ | 10.20 ± 1.54^a^ | 0.007 | 0.000 | 0.145 |
| **Chlb.p.65** | *mg g DW^-1^* | 2.54 ± 0.65^b^ | 2.77 ± 0.23^b^ | 3.15 ± 0.35^b^ | 3.34 ± 0.12^b^ | 3.11 ± 0.20^b^ | 3.21 ± 0.63^b^ | 6.89 ± 1.22^a^ | 2.90 ± 0.25^b^ | 3.84 ± 0.49^ab^ | 6.87 ± 1.17^a^ | 0.008 | 0.000 | 0.007 |
| **Chlb.a.65** | *mg g DW^-1^* | 2.12 ± 0.19^b^ | 1.80 ± 0.21^b^ | 1.95 ± 0.31^b^ | 1.91 ± 0.18^b^ | 1.84 ± 0.06^b^ | 1.55 ± 0.09^b^ | 3.73 ± 0.25^a^ | 2.44 ± 0.32^b^ | 1.88 ± 0.35^b^ | 2.33 ± 0.32^b^ | 0.009 | 0.008 | 0.001 |
| **Chlb.g.65** | *mg g DW^-1^* | 2.71 ± 0.26^ab^ | 2.11 ± 0.29^ab^ | 2.12 ± 0.20^ab^ | 2.23 ± 0.10^ab^ | 2.03 ± 0.19^ab^ | 2.07 ± 0.17^ab^ | 3.16 ± 0.42^a^ | 2.35 ± 0.12^ab^ | 1.95 ± 0.16^b^ | 3.17 ± 0.27^a^ | 0.145 | 0.061 | 0.003 |
| **Chlb.l.65** | *mg g DW^-1^* | 3.24 ± 0.43^ab^ | 2.70 ± 0.64^ab^ | 2.44 ± 0.20^b^ | 2.66 ± 0.21^b^ | 2.56 ± 0.29^b^ | 2.57 ± 0.26^b^ | 4.77 ± 0.56^a^ | 3.66 ± 0.54^ab^ | 2.17 ± 0.22^b^ | 4.24 ± 0.52^ab^ | 0.051 | 0.010 | 0.009 |
| **Chlb.b.75** | *mg g DW^-1^* | 3.52 ± 0.55 | 4.16 ± 0.83 | 3.96 ± 0.37 | 3.84 ± 0.21 | 8.96 ± 6.34 | 6.18 ± 3.20 | 8.29 ± 3.18 | 4.92 ± 1.84 | 3.43 ± 0.76 | 2.29 ± 0.19 | 0.867 | 0.934 | 0.294 |
| **Chlb.s.75** | *mg g DW^-1^* | 5.11 ± 1.40 | 6.05 ± 1.26 | 4.11 ± 0.56 | 3.92 ± 0.27 | 8.89 ± 3.31 | 8.97 ± 3.24 | 10.30 ± 1.56 | 8.44 ± 3.73 | 9.73 ± 1.65 | 6.59 ± 1.91 | 0.918 | 0.033 | 0.416 |
| **Chlb.p.75** | *mg g DW^-1^* | 5.98 ± 1.60 | 7.08 ± 1.90 | 4.81 ± 1.33 | 5.24 ± 0.79 | 6.43 ± 1.03 | 5.02 ± 1.34 | 7.50 ± 2.89 | 5.68 ± 1.55 | 7.25 ± 1.37 | 7.01 ± 0.92 | 0.684 | 0.563 | 0.920 |
| **Chlb.a.75** | *mg g DW^-1^* | 1.45 ± 0.17 | 1.81 ± 0.29 | 1.21 ± 0.03 | 1.73 ± 0.15 | 1.67 ± 0.42 | 1.77 ± 0.14 | 2.51 ± 0.42 | 2.44 ± 0.77 | 2.23 ± 0.17 | 2.26 ± 1.02 | 0.810 | 0.034 | 0.892 |
| **Chlb.g.75** | *mg g DW^-1^* | 1.79 ± 0.25^ab^ | 1.98 ± 0.30^ab^ | 1.27 ± 0.03^b^ | 1.82 ± 0.15^ab^ | 1.84 ± 0.12^ab^ | 2.08 ± 0.12^ab^ | 2.89 ± 0.40^a^ | 1.85 ± 0.40^ab^ | 2.35 ± 0.13^ab^ | 2.17 ± 0.43^ab^ | 0.058 | 0.005 | 0.787 |
| **Chlb.l.75** | *mg g DW^-1^* | 1.96 ± 0.21 | 1.7 ± 0.19 | 1.30 ± 0.05 | 1.55 ± 0.06 | 2.14 ± 0.35 | 2.29 ± 0.40 | 3.71 ± 0.47 | 2.08 ± 0.57 | 2.92 ± 0.29 | 2.85 ± 1.28 | 0.368 | 0.004 | 0.525 |
| **Chltot.b.65** | *mg g DW^-1^* | 12.9 ± 1.4^ab^ | 13.7 ± 0.5^ab^ | 13.0 ± 0.6^ab^ | 14.5 ± 1.1^ab^ | 11.1 ± 0.6^ab^ | 10.7 ± 0.8^b^ | 14.8 ± 0.8^ab^ | 16.5 ± 2.5^a^ | 12.9 ± 0.5^ab^ | 9.8 ± 0.9^b^ | 0.005 | 0.911 | 0.104 |
| **Chltot.s.65** | *mg g DW^-1^* | 8.7 ± 0.1^c^ | 11.3 ± 1.0^ac^ | 11.4 ± 0.5^ac^ | 9.0 ± 0.7^c^ | 10.7 ± 2.2^bc^ | 9.7 ± 0.9^c^ | 19.5 ± 3.0^a^ | 14.1 ± 2.0^ac^ | 15.5 ± 1.1^ac^ | 19.0 ± 2.4^ab^ | 0.010 | 0.000 | 0.139 |
| **Chltot.p.65** | *mg g DW^-1^* | 5.92 ± 1.39^c^ | 6.30 ± 0.61^bc^ | 7.31 ± 0.94^ac^ | 7.84 ± 0.18^ac^ | 6.03 ± 0.18^c^ | 6.83 ± 0.88^bc^ | 13.00 ± 2.18^a^ | 6.91 ± 0.56^bc^ | 7.45 ± 0.81^ac^ | 12.00 ± 1.96^ab^ | 0.062 | 0.002 | 0.010 |
| **Chltot.a.65** | *mg g DW^-1^* | 7.64 ± 1.05^ab^ | 6.54 ± 0.79^ab^ | 7.39 ± 1.52^ab^ | 7.61 ± 0.80^ab^ | 5.80 ± 0.27^ab^ | 4.87 ± 0.23^b^ | 9.58 ± 0.67^a^ | 7.39 ± 0.77^ab^ | 6.03 ± 0.94^ab^ | 5.85 ± 1.06^ab^ | 0.132 | 0.660 | 0.042 |
| **Chltot.g.65** | *mg g DW^-1^* | 6.67 ± 0.69 | 5.20 ± 0.66 | 5.83 ± 0.59 | 6.08 ± 0.19 | 5.22 ± 0.41 | 4.88 ± 0.33 | 6.83 ± 0.82 | 5.66 ± 0.30 | 4.64 ± 0.33 | 6.70 ± 0.55 | 0.747 | 0.863 | 0.008 |
| **Chltot.l.65** | *mg g DW^-1^* | 8.09 ± 1.05 | 6.65 ± 1.68 | 6.24 ± 0.64 | 7.25 ± 0.44 | 6.38 ± 0.63 | 5.80 ± 0.43 | 9.43 ± 1.03 | 7.94 ± 0.99 | 5.25 ± 0.61 | 8.68 ± 1.21 | 0.432 | 0.416 | 0.031 |


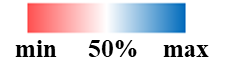
**Supplementary Table S1**. Continued.

| **Variable** | **Units** | **Irrigated** | | | | | **Rainfed** | | | | | ***P*-value** | | |
| --- | --- | --- | --- | --- | --- | --- | --- | --- | --- | --- | --- | --- | --- | --- |
|  |  | **MEX** | **EUR** | **DRI** | **KNI** | **HAR** | **MEX** | **EUR** | **DRI** | **KNI** | **HAR** | **G** | **W** | **G×W** |
| **Chltot.b.75** | *mg g DW^-1^* | 10.9 ± 1.1 | 11.5 ± 1.4 | 13.1 ± 0.9 | 13.5 ± 1.3 | 17.7 ± 9.8 | 12.4 ± 4.6 | 16.1 ± 4.8 | 10.4 ± 3.0 | 7.8 ± 0.9 | 6.0 ± 0.4 | 0.949 | 0.276 | 0.305 |
| **Chltot.s.75** | *mg g DW^-1^* | 10.9 ± 2.0 | 12.2 ± 2.0 | 10.2 ± 0.7 | 9.5 ± 0.3 | 16.5 ± 5.4 | 16.2 ± 4.6 | 17.3 ± 2.4 | 15.7 ± 6.1 | 17.3 ± 2.7 | 12.6 ± 3.1 | 0.980 | 0.083 | 0.509 |
| **Chltot.p.75** | *mg g DW^-1^* | 11.1 ± 2.4 | 13.4 ± 3.0 | 9.9 ± 2.2 | 10.5 ± 1.4 | 11.9 ± 2.0 | 9.5 ± 1.9 | 13.7 ± 4.8 | 10.7 ± 2.7 | 13.0 ± 2.3 | 12.1 ± 1.4 | 0.689 | 0.791 | 0.954 |
| **Chltot.a.75** | *mg g DW^-1^* | 4.45 ± 0.54 | 5.74 ± 1.17 | 4.29 ± 0.07 | 6.21 ± 0.79 | 4.37 ± 1.14 | 4.90 ± 0.31 | 6.13 ± 0.92 | 6.00 ± 1.54 | 5.60 ± 0.67 | 5.40 ± 2.18 | 0.697 | 0.404 | 0.873 |
| **Chltot.g.75** | *mg g DW^-1^* | 4.30 ± 0.55^ab^ | 4.63 ± 0.71^ab^ | 3.32 ± 0.18^b^ | 4.52 ± 0.24^ab^ | 4.25 ± 0.26^ab^ | 4.85 ± 0.39^ab^ | 6.42 ± 0.71^a^ | 4.16 ± 0.75^ab^ | 4.77 ± 0.19^ab^ | 4.91 ± 0.91^ab^ | 0.066 | 0.029 | 0.693 |
| **Chltot.l.75** | *mg g DW^-1^* | 4.70 ± 0.54 | 3.91 ± 0.43 | 3.36 ± 0.21 | 3.93 ± 0.24 | 4.66 ± 0.62 | 5.00 ± 0.73 | 7.32 ± 0.72 | 4.29 ± 0.88 | 5.90 ± 0.51 | 5.86 ± 2.42 | 0.426 | 0.016 | 0.537 |
| **Glc.b.65** | *µmol organ^-1^* | 1.64 ± 0.16^ab^ | 0.96 ± 0.32^b^ | 1.38 ± 0.63^b^ | 0.57 ± 0.05^b^ | 5.31 ± 1.94^a^ | 3.75 ± 0.32^ab^ | 0.78 ± 0.14^b^ | 2.28 ± 0.53^ab^ | 3.32 ± 0.87^ab^ | 1.39 ± 0.35^b^ | 0.037 | 0.487 | 0.002 |
| **Glc.s.65** | *µmol organ^-1^* | 9.11 ± 0.28^ac^ | 4.78 ± 1.40^bcd^ | 3.82 ± 1.77^cd^ | 6.24 ± 0.09^ad^ | 9.95 ± 1.30^ab^ | 10.60 ± 1.59^a^ | 2.02 ± 0.42^d^ | 6.25 ± 0.47^ad^ | 7.70 ± 1.23^ac^ | 8.12 ± 0.47^ac^ | 0.000 | 0.815 | 0.096 |
| **Glc.p.65** | *µmol organ^-1^* | 58.8 ± 18.7^ab^ | 67.1 ± 13.8^ab^ | 63.6 ± 9.1^ab^ | 80.5 ± 6.3^a^ | 60.8 ± 4.4^ab^ | 58.9 ± 17.2^ab^ | 13.0 ± 1.4^b^ | 56.5 ± 10.3^ab^ | 61.5 ± 14.5^ab^ | 28.4 ± 2.1^ab^ | 0.082 | 0.005 | 0.171 |
| **Glc.a.65** | *µmol organ^-1^* | 13.30 ± 3.57^ab^ | 5.29 ± 1.23^bc^ | 4.34 ± 0.80^bc^ | 8.75 ± 1.42^ac^ | 14.50 ± 1.27^ab^ | 15.50 ± 3.66^a^ | 3.02 ± 0.60^c^ | 6.65 ± 1.31^ac^ | 8.14 ± 1.31^ac^ | 6.40 ± 2.37^ac^ | 0.000 | 0.326 | 0.105 |
| **Glc.g.65** | *µmol organ^-1^* | 8.14 ± 1.54^a^ | 4.43 ± 0.97^ab^ | 6.00 ± 0.53^ab^ | 7.57 ± 0.79^a^ | 7.26 ± 0.15^ab^ | 6.36 ± 0.33^ab^ | 2.85 ± 0.64^b^ | 5.91 ± 0.04^ab^ | 5.73 ± 0.79^ab^ | 4.44 ± 1.58^ab^ | 0.007 | 0.009 | 0.659 |
| **Glc.l.65** | *µmol organ^-1^* | 17.1 ± 2.7^a^ | 7.2 ± 1.7^bc^ | 9.0 ± 2.0^ac^ | 13.5 ± 2.1^ab^ | 16.5 ± 1.6^a^ | 11.3 ± 0.8^ac^ | 2.9 ± 0.2^c^ | 8.6 ± 1.1^ac^ | 8.5 ± 1.7^ac^ | 7.3 ± 2.0^bc^ | 0.001 | 0.000 | 0.203 |
| **Glc.b.75** | *µmol organ^-1^* | 3.74 ± 1.40^b^ | 1.97 ± 0.51^b^ | 3.17 ± 1.34^b^ | 2.53 ± 1.23^b^ | 11.9 ± 1.74^a^ | 2.69 ± 0.56^b^ | 1.26 ± 0.10^b^ | 2.08 ± 0.51^b^ | 1.45 ± 0.48^b^ | 1.51 ± 0.57^b^ | 0.000 | 0.000 | 0.000 |
| **Glc.s.75** | *µmol organ^-1^* | 3.84 ± 0.97^b^ | 1.85 ± 0.58^b^ | 2.34 ± 0.84^b^ | 2.48 ± 0.57^b^ | 8.00 ± 0.54^a^ | 2.61 ± 0.25^b^ | 1.96 ± 0.16^b^ | 2.28 ± 0.35^b^ | 2.08 ± 0.46^b^ | 3.95 ± 0.69^b^ | 0.000 | 0.007 | 0.012 |
| **Glc.p.75** | *µmol organ^-1^* | 15.6 ± 9.1 | 11.1 ± 6.0 | 14.2 ± 5.3 | 8.1 ± 1.7 | 21.1 ± 5.8 | 5.3 ± 0.5 | 3.9 ± 1.2 | 4.9 ± 1.0 | 4.5 ± 1.6 | 12.4 ± 7.4 | 0.284 | 0.021 | 0.964 |
| **Glc.a.75** | *µmol organ^-1^* | 5.10 ± 2.06^b^ | 4.04 ± 2.61^b^ | 2.66 ± 1.03^b^ | 2.37 ± 0.79^b^ | 14.90 ± 3.10^a^ | 3.43 ± 0.31^b^ | 2.63 ± 0.85^b^ | 1.61 ± 0.41^b^ | 1.41 ± 0.25^b^ | 7.90 ± 3.69^ab^ | 0.000 | 0.061 | 0.483 |
| **Glc.g.75** | *µmol organ^-1^* | 3.22 ± 1.11^ab^ | 2.83 ± 1.28^ab^ | 4.51 ± 1.37^ab^ | 2.42 ± 0.45^ab^ | 5.12 ± 0.50^a^ | 2.20 ± 0.29^ab^ | 1.73 ± 0.42^ab^ | 2.40 ± 0.76^ab^ | 0.87 ± 0.17^b^ | 2.57 ± 0.87^ab^ | 0.094 | 0.005 | 0.861 |
| **Glc.l.75** | *µmol organ^-1^* | 5.04 ± 1.87^ab^ | 3.34 ± 1.75^ab^ | 3.94 ± 1.01^ab^ | 2.26 ± 0.45^b^ | 7.63 ± 0.91^a^ | 2.15 ± 0.23^b^ | 1.49 ± 0.27^b^ | 2.32 ± 0.76^ab^ | 1.39 ± 0.19^b^ | 3.66 ± 1.38^ab^ | 0.018 | 0.003 | 0.634 |
| **Glc6P.b.65** | *µmol organ^-1^* | 0.184 ± 0.017^ab^ | 0.176 ± 0.013^ab^ | 0.188 ± 0.024^ab^ | 0.135 ± 0.009^bc^ | 0.217 ± 0.020^a^ | 0.074 ± 0.016^cd^ | 0.062 ± 0.002^d^ | 0.076 ± 0.007^cd^ | 0.060 ± 0.013^d^ | 0.068 ± 0.004^cd^ | 0.046 | 0.000 | 0.175 |
| **Glc6P.s.65** | *µmol organ^-1^* | 0.235 ± 0.002^a^ | 0.179 ± 0.013^a^ | 0.208 ± 0.018^a^ | 0.207 ± 0.021^a^ | 0.195 ± 0.022^a^ | 0.104 ± 0.001^b^ | 0.072 ± 0.001^b^ | 0.089 ± 0.003^b^ | 0.094 ± 0.008^b^ | 0.097 ± 0.007^b^ | 0.036 | 0.000 | 0.737 |
| **Glc6P.p.65** | *µmol organ^-1^* | 0.442 ± 0.127^ac^ | 0.527 ± 0.083^ab^ | 0.502 ± 0.016^ab^ | 0.566 ± 0.064^a^ | 0.372 ± 0.027^ac^ | 0.301 ± 0.083^ac^ | 0.173 ± 0.007^c^ | 0.279 ± 0.051^ac^ | 0.291 ± 0.052^ac^ | 0.215 ± 0.018^bc^ | 0.326 | 0.000 | 0.459 |
| **Glc6P.a.65** | *µmol organ^-1^* | 0.323 ± 0.062^a^ | 0.210 ± 0.027^ac^ | 0.196 ± 0.029^ac^ | 0.221 ± 0.028^ac^ | 0.291 ± 0.027^ab^ | 0.206 ± 0.007^ac^ | 0.164 ± 0.010^bc^ | 0.169 ± 0.009^bc^ | 0.113 ± 0.005^c^ | 0.189 ± 0.008^ac^ | 0.007 | 0.000 | 0.366 |
| **Glc6P.g.65** | *µmol organ^-1^* | 0.164 ± 0.025^ab^ | 0.106 ± 0.010^ac^ | 0.170 ± 0.023^a^ | 0.146 ± 0.008^ac^ | 0.168 ± 0.020^a^ | 0.103 ± 0.001^ac^ | 0.086 ± 0.004^c^ | 0.137 ± 0.008^ac^ | 0.094 ± 0.006^c^ | 0.099 ± 0.010^bc^ | 0.007 | 0.000 | 0.383 |
| **Glc6P.l.65** | *µmol organ^-1^* | 0.296 ± 0.038^a^ | 0.178 ± 0.018^bcd^ | 0.221 ± 0.026^ac^ | 0.221 ± 0.028^ac^ | 0.250 ± 0.007^ab^ | 0.145 ± 0.007^cd^ | 0.120 ± 0.007^d^ | 0.177 ± 0.009^bcd^ | 0.115 ± 0.012^d^ | 0.135 ± 0.008^cd^ | 0.012 | 0.000 | 0.067 |
| **Glc6P.b.75** | *µmol organ^-1^* | 0.121 ± 0.005^ac^ | 0.147 ± 0.021^ac^ | 0.185 ± 0.002^ab^ | 0.147 ± 0.023^ac^ | 0.200 ± 0.030^a^ | 0.052 ± 0.003^c^ | 0.075 ± 0.007^bc^ | 0.082 ± 0.006^bc^ | 0.055 ± 0.016^c^ | 0.116 ± 0.053^ac^ | 0.036 | 0.000 | 0.938 |
| **Glc6P.s.75** | *µmol organ^-1^* | 0.098 ± 0.013 | 0.086 ± 0.003 | 0.134 ± 0.018 | 0.096 ± 0.017 | 0.121 ± 0.005 | 0.075 ± 0.006 | 0.074 ± 0.001 | 0.101 ± 0.012 | 0.076 ± 0.011 | 0.143 ± 0.031 | 0.006 | 0.151 | 0.399 |
| **Glc6P.p.75** | *µmol organ^-1^* | 0.206 ± 0.028 | 0.193 ± 0.027 | 0.261 ± 0.042 | 0.209 ± 0.031 | 0.300 ± 0.064 | 0.153 ± 0.015 | 0.124 ± 0.003 | 0.169 ± 0.020 | 0.149 ± 0.019 | 0.301 ± 0.070 | 0.009 | 0.033 | 0.795 |
| **Glc6P.a.75** | *µmol organ^-1^* | 0.191 ± 0.008^ac^ | 0.170 ± 0.030^ac^ | 0.128 ± 0.002^c^ | 0.141 ± 0.018^bc^ | 0.311 ± 0.064^ab^ | 0.154 ± 0.017^ac^ | 0.142 ± 0.012^bc^ | 0.114 ± 0.005^c^ | 0.093 ± 0.007^c^ | 0.325 ± 0.085^a^ | 0.000 | 0.335 | 0.923 |
| **Glc6P.g.75** | *µmol organ^-1^* | 0.107 ± 0.007^ab^ | 0.075 ± 0.007^b^ | 0.132 ± 0.004^ab^ | 0.102 ± 0.019^ab^ | 0.114 ± 0.009^ab^ | 0.110 ± 0.012^ab^ | 0.083 ± 0.005^b^ | 0.127 ± 0.011^ab^ | 0.104 ± 0.007^ab^ | 0.171 ± 0.035^a^ | 0.003 | 0.176 | 0.235 |
| **Glc6P.l.75** | *µmol organ^-1^* | 0.136 ± 0.007 | 0.097 ± 0.018 | 0.124 ± 0.006 | 0.105 ± 0.014 | 0.133 ± 0.020 | 0.110 ± 0.002 | 0.118 ± 0.002 | 0.130 ± 0.009 | 0.111 ± 0.011 | 0.180 ± 0.041 | 0.050 | 0.323 | 0.331 |
| **Fru.b.65** | *µmol organ^-1^* | 2.03 ± 0.23^ab^ | 0.97 ± 0.38^b^ | 1.32 ± 0.55^ab^ | 1.22 ± 0.08^b^ | 4.35 ± 1.49^a^ | 3.07 ± 0.50^ab^ | 0.65 ± 0.13^b^ | 1.50 ± 0.38^ab^ | 2.28 ± 0.78^ab^ | 1.10 ± 0.20^b^ | 0.029 | 0.519 | 0.013 |
| **Fru.s.65** | *µmol organ^-1^* | 4.73 ± 0.44^ab^ | 3.48 ± 0.96^ab^ | 3.03 ± 1.45^b^ | 3.46 ± 0.17^ab^ | 6.96 ± 1.07^a^ | 4.88 ± 0.59^ab^ | 1.27 ± 0.21^b^ | 3.33 ± 0.42^ab^ | 3.54 ± 0.71^ab^ | 3.43 ± 0.51^ab^ | 0.007 | 0.041 | 0.065 |
| **Fru.p.65** | *µmol organ^-1^* | 30.9 ± 9.8^ab^ | 36.2 ± 6.2^a^ | 35.8 ± 3.5^ab^ | 42.6 ± 4.4^a^ | 32.3 ± 1.02^ab^ | 28.7 ± 8.7^ab^ | 8.5 ± 0.3^b^ | 29.0 ± 5.27^ab^ | 26.0 ± 5.3^ab^ | 15.8 ± 1.0^ab^ | 0.169 | 0.001 | 0.205 |
| **Fru.a.65** | *µmol organ^-1^* | 2.14 ± 0.48 | 1.47 ± 0.67 | 0.90 ± 0.15 | 1.33 ± 0.26 | 2.36 ± 0.42 | 2.49 ± 0.71 | 1.08 ± 0.16 | 1.36 ± 0.41 | 1.35 ± 0.20 | 1.48 ± 0.52 | 0.069 | 0.764 | 0.550 |
| **Fru.g.65** | *µmol organ^-1^* | 1.90 ± 0.28 | 1.84 ± 0.60 | 1.76 ± 0.19 | 1.72 ± 0.20 | 2.30 ± 0.27 | 2.10 ± 0.22 | 1.78 ± 0.30 | 1.93 ± 0.06 | 2.12 ± 0.32 | 2.56 ± 0.97 | 0.603 | 0.484 | 0.988 |
| **Fru.l.65** | *µmol organ^-1^* | 6.67 ± 1.17^ab^ | 4.17 ± 0.60^ac^ | 4.01 ± 0.76^ac^ | 4.92 ± 0.85^ac^ | 7.78 ± 1.11^a^ | 4.48 ± 0.42^ac^ | 2.35 ± 0.20^c^ | 3.74 ± 0.36^bc^ | 3.59 ± 0.63^bc^ | 4.05 ± 0.83^ac^ | 0.009 | 0.001 | 0.269 |
| **Fru.b.75** | *µmol organ^-1^* | 4.16 ± 1.41^b^ | 2.81 ± 0.83^b^ | 3.83 ± 1.19^b^ | 2.30 ± 0.93^b^ | 13.90 ± 2.75^a^ | 4.94 ± 1.20^b^ | 1.98 ± 0.29^b^ | 4.61 ± 2.49^b^ | 3.46 ± 0.85^b^ | 1.56 ± 0.57^b^ | 0.013 | 0.034 | 0.001 |
| **Fru.s.75** | *µmol organ^-1^* | 6.06 ± 1.81^b^ | 5.24 ± 2.63^b^ | 6.09 ± 3.29^b^ | 3.39 ± 0.78^b^ | 18.40 ± 4.15^a^ | 4.78 ± 0.29^b^ | 3.86 ± 0.46^b^ | 4.65 ± 1.90^b^ | 2.91 ± 0.68^b^ | 8.50 ± 3.13^ab^ | 0.002 | 0.061 | 0.256 |
| **Fru.p.75** | *µmol organ^-1^* | 19.0 ± 5.4^ab^ | 12.0 ± 3.8^b^ | 13.7 ± 4.3^ab^ | 10.7 ± 3.6^b^ | 33.4 ± 7.4^a^ | 6.2 ± 0.9^b^ | 4.7 ± 1.5^b^ | 5.8 ± 1.1^b^ | 5.5 ± 1.3^b^ | 17.4 ± 5.6^ab^ | 0.002 | 0.001 | 0.674 |
| **Fru.a.75** | *µmol organ^-1^* | 2.63 ± 1.04^b^ | 1.99 ± 1.18^b^ | 1.18 ± 0.28^b^ | 0.99 ± 0.31^b^ | 10.40 ± 1.87^a^ | 2.05 ± 0.28^b^ | 1.62 ± 0.40^b^ | 1.74 ± 0.47^b^ | 1.04 ± 0.18^b^ | 6.61 ± 2.51^ab^ | 0.000 | 0.268 | 0.376 |
| **Fru.g.75** | *µmol organ^-1^* | 1.69 ± 0.69^bc^ | 1.84 ± 0.80^bc^ | 2.40 ± 0.69^bc^ | 1.35 ± 0.28^bc^ | 5.92 ± 0.96^a^ | 1.51 ± 0.16^bc^ | 1.26 ± 0.28^bc^ | 1.93 ± 0.66^bc^ | 0.76 ± 0.09^c^ | 4.32 ± 1.26^ab^ | 0.000 | 0.131 | 0.871 |
| **Fru.l.75** | *µmol organ^-1^* | 3.23 ± 1.27^ab^ | 2.69 ± 1.15^b^ | 2.47 ± 0.54^b^ | 1.46 ± 0.12^b^ | 6.94 ± 0.89^a^ | 2.32 ± 0.18^b^ | 1.88 ± 0.37^b^ | 2.44 ± 0.71^b^ | 1.46 ± 0.14^b^ | 4.54 ± 1.32^ab^ | 0.000 | 0.119 | 0.582 |
| **Suc.b.65** | *µmol organ^-1^* | 10.9 ± 1.6^b^ | 15.0 ± 2.8^b^ | 18.5 ± 2.7^ab^ | 10.5 ± 1.0^b^ | 28.4 ± 3.9^a^ | 11.7 ± 3.4^b^ | 16.5 ± 0.6^b^ | 15.2 ± 0.5^b^ | 11.2 ± 1.9^b^ | 19.1 ± 1.1^ab^ | 0.000 | 0.195 | 0.129 |
| **Suc.s.65** | *µmol organ^-1^* | 22.2 ± 1.3^c^ | 24.9 ± 2.1^bc^ | 27.0 ± 2.3^ac^ | 20.4 ± 2.5^c^ | 36.8 ± 2.6^a^ | 27.2 ± 3.6^ac^ | 27.6 ± 0.5^ac^ | 29.1 ± 1.7^ac^ | 26.4 ± 3.1^ac^ | 35.9 ± 1.7^ab^ | 0.000 | 0.057 | 0.607 |
| **Suc.p.65** | *µmol organ^-1^* | 23.1 ± 6.8^b^ | 46.5 ± 10.1^ab^ | 57.5 ± 8.4^a^ | 27.3 ± 5.0^b^ | 38.5 ± 1.1^ab^ | 28.6 ± 2.0^b^ | 49.0 ± 3.0^ab^ | 29.4 ± 1.9^b^ | 34.4 ± 3.4^ab^ | 44.2 ± 1.3^ab^ | 0.002 | 0.661 | 0.014 |
| **Suc.a.65** | *µmol organ^-1^* | 12.9 ± 2.2^de^ | 18.1 ± 0.7^bcd^ | 15.6 ± 2.1^ce^ | 10.0 ± 1.7^e^ | 21.5 ± 1.5^ac^ | 15.8 ± 1.0^ce^ | 24.8 ± 1.8^ab^ | 19.1 ± 1.1^acd^ | 9.8 ± 0.8^e^ | 25.9 ± 1.8^a^ | 0.000 | 0.002 | 0.307 |
| **Suc.g.65** | *µmol organ^-1^* | 6.6 ± 0.3^de^ | 7.7 ± 1.3^de^ | 14.1 ± 1.4^bc^ | 5.7 ± 0.7^e^ | 17.0 ± 0.9^ab^ | 10.3 ± 0.9^cd^ | 13.7 ± 1.0^bc^ | 16.4 ± 0.2^ab^ | 8.9 ± 0.6^de^ | 20.2 ± 0.8^a^ | 0.000 | 0.000 | 0.335 |
| **Suc.l.65** | *µmol organ^-1^* | 11.3 ± 1.1^bc^ | 11.3 ± 1.5^bc^ | 15.2 ± 2.1^ac^ | 8.8 ± 1.4^c^ | 17.2 ± 0.6^ab^ | 13.9 ± 1.7^ac^ | 19.1 ± 1.0^a^ | 20.5 ± 1.8^a^ | 10.4 ± 0.7^bc^ | 21.0 ± 1.6^a^ | 0.000 | 0.000 | 0.255 |


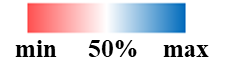
**Supplementary Table S1**. Continued.

| **Variable** | **Units** | **Irrigated** | | | | | **Rainfed** | | | | | ***P*-value** | | |
| --- | --- | --- | --- | --- | --- | --- | --- | --- | --- | --- | --- | --- | --- | --- |
|  |  | **MEX** | **EUR** | **DRI** | **KNI** | **HAR** | **MEX** | **EUR** | **DRI** | **KNI** | **HAR** | **G** | **W** | **G×W** |
| **Suc.b.75** | *µmol organ^-1^* | 12.5 ± 2.4 | 17.0 ± 3.8 | 22.5 ± 1.8 | 12.8 ± 2.3 | 21.8 ± 5.2 | 9.1 ± 1.2 | 16.1 ± 1.4 | 17.4 ± 1.0 | 11.4 ± 3.7 | 20.7 ± 8.8 | 0.051 | 0.343 | 0.978 |
| **Suc.s.75** | *µmol organ^-1^* | 25.3 ± 1.1 | 23.0 ± 2.7 | 31.7 ± 0.4 | 26.2 ± 3.4 | 33.0 ± 3.1 | 24.4 ± 2.4 | 27.1 ± 1.3 | 33.5 ± 1.2 | 22.6 ± 3.7 | 33.4 ± 7.2 | 0.018 | 0.857 | 0.810 |
| **Suc.p.75** | *µmol organ^-1^* | 93.6 ± 11.5^ac^ | 77.9 ± 3.2^ac^ | 115.0 ± 3.3^a^ | 110.0 ± 20.6^ab^ | 118.0 ± 17.0^a^ | 52.4 ± 7.4^c^ | 57.8 ± 4.4^bc^ | 82.5 ± 7.0^ac^ | 61.8 ± 12.6^ac^ | 77.8 ± 12.1^ac^ | 0.040 | 0.000 | 0.779 |
| **Suc.a.75** | *µmol organ^-1^* | 14.2 ± 0.8^bc^ | 13.4 ± 2.4^bc^ | 8.8 ± 1.2^bc^ | 8.2 ± 1.1^c^ | 23.0 ± 2.3^ab^ | 20.3 ± 3.0^ac^ | 20.5 ± 1.0^ac^ | 19.5 ± 2.2^ac^ | 13.1 ± 1.5^bc^ | 32.5 ± 7.3^a^ | 0.000 | 0.000 | 0.853 |
| **Suc.g.75** | *µmol organ^-1^* | 7.7 ± 0.6^c^ | 7.0 ± 0.9^c^ | 9.4 ± 1.2^c^ | 7.0 ± 1.1^c^ | 17.2 ± 0.7^ab^ | 11.9 ± 0.5^bc^ | 12.5 ± 0.3^bc^ | 16.0 ± 2.1^ab^ | 10.2 ± 0.4^c^ | 19.3 ± 2.0^a^ | 0.000 | 0.000 | 0.324 |
| **Suc.l.75** | *µmol organ^-1^* | 12.0 ± 1.0^bd^ | 10.1 ± 1.1^cd^ | 10.1 ± 0.6^cd^ | 7.0 ± 1.0^d^ | 16.7 ± 1.2^ab^ | 14.6 ± 1.3^abc^ | 16.0 ± 0.3^abc^ | 17.8 ± 1.9^ab^ | 13.8 ± 1.1^abc^ | 18.9 ± 2.3^a^ | 0.000 | 0.000 | 0.157 |
| **starch.b.65** | *µmol organ^-1^* | 1.22 ± 0.22 | 1.35 ± 0.14 | 2.41 ± 0.20 | 1.47 ± 0.19 | 3.59 ± 2.11 | 0.56 ± 0.33 | 3.40 ± 0.44 | 1.53 ± 0.16 | 0.37 ± 0.05 | 1.68 ± 0.43 | 0.067 | 0.284 | 0.104 |
| **starch.s.65** | *µmol organ^-1^* | 0.65 ± 0.04^bc^ | 0.71 ± 0.20^bc^ | 1.13 ± 0.22^bc^ | 0.64 ± 0.10^bc^ | 1.41 ± 0.69^ac^ | 0.55 ± 0.19^c^ | 2.24 ± 0.34^ab^ | 0.99 ± 0.19^bc^ | 0.64 ± 0.09^bc^ | 2.84 ± 0.55^a^ | 0.001 | 0.017 | 0.028 |
| **starch.p.65** | *µmol organ^-1^* | 1.56 ± 0.34^bc^ | 2.51 ± 0.56^ac^ | 2.50 ± 0.48^ac^ | 2.43 ± 0.66^ac^ | 1.72 ± 0.20^bc^ | 1.09 ± 0.13^c^ | 3.20 ± 0.14^ab^ | 1.72 ± 0.25^bc^ | 1.36 ± 0.29^bc^ | 4.28 ± 0.49^a^ | 0.002 | 0.461 | 0.001 |
| **starch.a.65** | *µmol organ^-1^* | 2.04 ± 0.47^bc^ | 2.93 ± 0.54^ac^ | 2.40 ± 0.44^ac^ | 1.56 ± 0.31^c^ | 3.51 ± 1.10^ac^ | 2.17 ± 0.37^ac^ | 5.02 ± 0.40^a^ | 3.07 ± 0.26^ac^ | 1.36 ± 0.15^c^ | 4.67 ± 1.01^ab^ | 0.001 | 0.051 | 0.348 |
| **starch.g.65** | *µmol organ^-1^* | 0.74 ± 0.04^b^ | 0.68 ± 0.23^b^ | 0.85 ± 0.11^b^ | 0.64 ± 0.11^b^ | 1.23 ± 0.33^ab^ | 0.86 ± 0.18^b^ | 1.07 ± 0.14^b^ | 1.31 ± 0.14^ab^ | 0.75 ± 0.11^b^ | 2.03 ± 0.15^a^ | 0.000 | 0.002 | 0.274 |
| **starch.l.65** | *µmol organ^-1^* | 3.03 ± 0.44^bc^ | 2.03 ± 0.46^bc^ | 3.11 ± 0.42^bc^ | 1.77 ± 0.26^bc^ | 3.81 ± 0.36^ab^ | 2.39 ± 0.38^bc^ | 3.48 ± 0.64^ac^ | 3.82 ± 0.38^ab^ | 1.56 ± 0.23^c^ | 5.33 ± 0.47^a^ | 0.000 | 0.043 | 0.061 |
| **starch.b.75** | *µmol organ^-1^* | 1.000 ± 0.638 | 1.410 ± 0.723 | 1.620 ± 0.733 | 1.440 ± 0.891 | 0.657 ± 0.235 | 0.282 ± 0.090 | 0.939 ± 0.056 | 0.868 ± 0.185 | 0.418 ± 0.158 | 2.380 ± 0.994 | 0.630 | 0.509 | 0.157 |
| **starch.s.75** | *µmol organ^-1^* | 0.289 ± 0.141^b^ | 0.489 ± 0.276^b^ | 0.545 ± 0.117^ab^ | 0.392 ± 0.200^b^ | 0.258 ± 0.042^b^ | 0.435 ± 0.109^b^ | 0.719 ± 0.064^ab^ | 0.749 ± 0.151^ab^ | 0.608 ± 0.045^ab^ | 1.250 ± 0.186^a^ | 0.137 | 0.001 | 0.053 |
| **starch.p.75** | *µmol organ^-1^* | 0.364 ± 0.099 | 0.419 ± 0.192 | 0.436 ± 0.150 | 0.626 ± 0.120 | 0.555 ± 0.168 | 0.379 ± 0.065 | 0.498 ± 0.108 | 0.772 ± 0.112 | 0.480 ± 0.074 | 0.637 ± 0.170 | 0.366 | 0.390 | 0.503 |
| **starch.a.75** | *µmol organ^-1^* | 1.59 ± 0.40 | 1.42 ± 0.72 | 1.11 ± 0.24 | 0.84 ± 0.18 | 1.61 ± 0.32 | 2.89 ± 0.69 | 2.81 ± 0.39 | 2.76 ± 0.39 | 1.97 ± 0.29 | 2.84 ± 0.38 | 0.315 | 0.000 | 0.980 |
| **starch.g.75** | *µmol organ^-1^* | 0.49 ± 0.11^c^ | 0.49 ± 0.19^c^ | 0.47 ± 0.15^c^ | 0.54 ± 0.16^bc^ | 0.88 ± 0.09^ac^ | 1.07 ± 0.13^ac^ | 1.07 ± 0.09^ac^ | 1.32 ± 0.33^ab^ | 0.95 ± 0.05^ac^ | 1.61 ± 0.14^a^ | 0.031 | 0.000 | 0.718 |
| **starch.l.75** | *µmol organ^-1^* | 1.26 ± 0.25^b^ | 1.42 ± 0.39^b^ | 1.11 ± 0.13^b^ | 0.71 ± 0.15^b^ | 1.11 ± 0.17^b^ | 1.93 ± 0.13^ab^ | 3.04 ± 0.77^a^ | 2.19 ± 0.18^ab^ | 1.74 ± 0.15^ab^ | 2.03 ± 0.13^ab^ | 0.055 | 0.000 | 0.646 |
| **malate.b.65** | *µmol organ^-1^* | 1.39 ± 0.13^a^ | 0.56 ± 0.03^b^ | 1.02 ± 0.13^ab^ | 0.94 ± 0.05^ab^ | 0.97 ± 0.14^ab^ | 0.86 ± 0.20^ab^ | 0.42 ± 0.04^b^ | 0.64 ± 0.06^b^ | 0.65 ± 0.22^b^ | 0.54 ± 0.04^b^ | 0.001 | 0.000 | 0.590 |
| **malate.s.65** | *µmol organ^-1^* | 1.97 ± 0.10^a^ | 1.22 ± 0.15^bd^ | 1.44 ± 0.16^ab^ | 1.53 ± 0.07^ab^ | 1.38 ± 0.02^bc^ | 1.37 ± 0.05^bc^ | 0.75 ± 0.07^d^ | 1.07 ± 0.12^bd^ | 1.20 ± 0.13^bd^ | 0.87 ± 0.13^cd^ | 0.000 | 0.000 | 0.717 |
| **malate.p.65** | *µmol organ^-1^* | 1.49 ± 0.45^ab^ | 1.42 ± 0.34^ab^ | 1.41 ± 0.08^ab^ | 1.99 ± 0.31^a^ | 1.49 ± 0.20^ab^ | 1.09 ± 0.42^ab^ | 0.23 ± 0.07^b^ | 0.93 ± 0.25^ab^ | 0.88 ± 0.18^ab^ | 0.57 ± 0.02^b^ | 0.235 | 0.000 | 0.497 |
| **malate.a.65** | *µmol organ^-1^* | 0.483 ± 0.126^a^ | 0.141 ± 0.038^c^ | 0.139 ± 0.019^c^ | 0.382 ± 0.032^ac^ | 0.447 ± 0.079^ab^ | 0.276 ± 0.047^ac^ | 0.132 ± 0.006^c^ | 0.200 ± 0.008^bc^ | 0.144 ± 0.026^c^ | 0.183 ± 0.026^bc^ | 0.001 | 0.001 | 0.019 |
| **malate.g.65** | *µmol organ^-1^* | 0.085 ± 0.029 | 0.038 ± 0.003 | 0.062 ± 0.007 | 0.103 ± 0.016 | 0.142 ± 0.029 | 0.162 ± 0.065 | 0.114 ± 0.014 | 0.117 ± 0.016 | 0.135 ± 0.026 | 0.113 ± 0.024 | 0.287 | 0.028 | 0.331 |
| **malate.l.65** | *µmol organ^-1^* | 0.416 ± 0.048^ab^ | 0.153 ± 0.046^cd^ | 0.191 ± 0.008^bcd^ | 0.376 ± 0.058^ac^ | 0.427 ± 0.041^a^ | 0.260 ± 0.058^ad^ | 0.134 ± 0.004^d^ | 0.22 ± 0.045^ad^ | 0.243 ± 0.062^ad^ | 0.213 ± 0.046^ad^ | 0.001 | 0.003 | 0.082 |
| **malate.b.75** | *µmol organ^-1^* | 1.00 ± 0.06^ac^ | 0.62 ± 0.04^bc^ | 1.21 ± 0.16^ab^ | 1.00 ± 0.11^ac^ | 1.40 ± 0.15^a^ | 0.51 ± 0.13^bc^ | 0.41 ± 0.02^c^ | 0.69 ± 0.16^ac^ | 0.54 ± 0.17^bc^ | 0.80 ± 0.30^ac^ | 0.010 | 0.000 | 0.744 |
| **malate.s.75** | *µmol organ^-1^* | 1.22 ± 0.11 | 0.97 ± 0.14 | 1.35 ± 0.16 | 1.21 ± 0.16 | 1.26 ± 0.09 | 0.82 ± 0.10 | 0.78 ± 0.07 | 1.06 ± 0.24 | 0.77 ± 0.09 | 1.01 ± 0.14 | 0.176 | 0.002 | 0.894 |
| **malate.p.75** | *µmol organ^-1^* | 0.353 ± 0.054^ab^ | 0.230 ± 0.066^ab^ | 0.466 ± 0.123^a^ | 0.228 ± 0.048^ab^ | 0.263 ± 0.096^ab^ | 0.190 ± 0.023^ab^ | 0.086 ± 0.005^b^ | 0.275 ± 0.058^ab^ | 0.124 ± 0.018^b^ | 0.323 ± 0.070^ab^ | 0.023 | 0.016 | 0.361 |
| **malate.a.75** | *µmol organ^-1^* | 0.333 ± 0.017^bc^ | 0.270 ± 0.062^bd^ | 0.192 ± 0.013^cd^ | 0.334 ± 0.025^bc^ | 0.673 ± 0.020^a^ | 0.099 ± 0.008^d^ | 0.124 ± 0.014^cd^ | 0.080 ± 0.007^d^ | 0.108 ± 0.010^d^ | 0.432 ± 0.117^b^ | 0.000 | 0.000 | 0.487 |
| **malate.g.75** | *µmol organ^-1^* | 0.082 ± 0.015^ab^ | 0.057 ± 0.024^b^ | 0.086 ± 0.016^ab^ | 0.131 ± 0.013^ab^ | 0.132 ± 0.019^a^ | 0.087 ± 0.007^ab^ | 0.089 ± 0.006^ab^ | 0.101 ± 0.013^ab^ | 0.096 ± 0.001^ab^ | 0.102 ± 0.019^ab^ | 0.028 | 0.727 | 0.171 |
| **malate.l.75** | *µmol organ^-1^* | 0.165 ± 0.015^ac^ | 0.078 ± 0.031^c^ | 0.125 ± 0.017^bc^ | 0.175 ± 0.007^ac^ | 0.261 ± 0.025^a^ | 0.098 ± 0.003^bc^ | 0.102 ± 0.005^bc^ | 0.144 ± 0.010^bc^ | 0.127 ± 0.004^bc^ | 0.188 ± 0.049^ab^ | 0.000 | 0.048 | 0.092 |
| **Glu.b.65** | *µmol organ^-1^* | 2.26 ± 0.39^ac^ | 2.31 ± 0.34^ac^ | 2.98 ± 0.45^a^ | 2.15 ± 0.07^ac^ | 2.81 ± 0.07^ab^ | 1.33 ± 0.48^c^ | 1.13 ± 0.04^c^ | 1.46 ± 0.09^bc^ | 1.07 ± 0.31^c^ | 1.06 ± 0.15^c^ | 0.294 | 0.000 | 0.621 |
| **Glu.s.65** | *µmol organ^-1^* | 2.15 ± 0.12^ab^ | 1.94 ± 0.16^abc^ | 2.60 ± 0.17^a^ | 1.90 ± 0.03^bc^ | 2.09 ± 0.01^abc^ | 1.68 ± 0.13^bd^ | 1.20 ± 0.12^d^ | 1.60 ± 0.11^bd^ | 1.59 ± 0.25^bd^ | 1.43 ± 0.12^cd^ | 0.013 | 0.000 | 0.150 |
| **Glu.p.65** | *µmol organ^-1^* | 2.22 ± 0.69 | 3.73 ± 0.21 | 4.06 ± 0.72 | 3.13 ± 0.43 | 2.38 ± 0.10 | 2.16 ± 0.52 | 2.37 ± 0.10 | 2.37 ± 0.28 | 2.42 ± 0.37 | 2.07 ± 0.12 | 0.072 | 0.005 | 0.281 |
| **Glu.a.65** | *µmol organ^-1^* | 1.36 ± 0.32^a^ | 1.17 ± 0.10^ab^ | 1.01 ± 0.09^ab^ | 1.20 ± 0.20^ab^ | 1.39 ± 0.14^a^ | 0.87 ± 0.04^ab^ | 0.98 ± 0.11^ab^ | 1.07 ± 0.03^ab^ | 0.58 ± 0.10^b^ | 0.78 ± 0.04^ab^ | 0.567 | 0.001 | 0.105 |
| **Glu.g.65** | *µmol organ^-1^* | 0.62 ± 0.08^c^ | 0.66 ± 0.09^c^ | 0.98 ± 0.14^ac^ | 0.76 ± 0.05^bc^ | 1.21 ± 0.07^a^ | 0.76 ± 0.07^bc^ | 0.88 ± 0.03^ac^ | 0.91 ± 0.02^ac^ | 0.75 ± 0.05^bc^ | 1.11 ± 0.11^ab^ | 0.000 | 0.474 | 0.252 |
| **Glu.l.65** | *µmol organ^-1^* | 1.29 ± 0.20 | 1.02 ± 0.08 | 1.37 ± 0.22 | 1.15 ± 0.17 | 1.54 ± 0.05 | 0.93 ± 0.06 | 1.15 ± 0.08 | 1.17 ± 0.21 | 0.90 ± 0.15 | 1.00 ± 0.09 | 0.345 | 0.014 | 0.261 |
| **Glu.b.75** | *µmol organ^-1^* | 1.65 ± 0.14^be^ | 2.04 ± 0.08^abc^ | 3.12 ± 0.21^a^ | 1.88 ± 0.19^bd^ | 2.27 ± 0.35^ab^ | 0.66 ± 0.11^e^ | 0.79 ± 0.11^de^ | 0.89 ± 0.06^cde^ | 0.61 ± 0.21^e^ | 1.26 ± 0.54^be^ | 0.011 | 0.000 | 0.106 |
| **Glu.s.75** | *µmol organ^-1^* | 1.30 ± 0.18 | 1.19 ± 0.35 | 1.75 ± 0.22 | 1.42 ± 0.19 | 1.52 ± 0.19 | 0.94 ± 0.08 | 0.90 ± 0.04 | 1.09 ± 0.09 | 0.90 ± 0.11 | 1.33 ± 0.29 | 0.206 | 0.004 | 0.764 |
| **Glu.p.75** | *µmol organ^-1^* | 2.85 ± 0.30 | 3.53 ± 0.94 | 3.57 ± 0.50 | 3.65 ± 0.39 | 2.95 ± 0.50 | 2.03 ± 0.29 | 1.99 ± 0.11 | 2.63 ± 0.34 | 1.94 ± 0.48 | 2.00 ± 0.42 | 0.633 | 0.001 | 0.832 |
| **Glu.a.75** | *µmol organ^-1^* | 1.07 ± 0.12 | 1.29 ± 0.45 | 0.857 ± 0.074 | 1.09 ± 0.07 | 1.42 ± 0.36 | 0.74 ± 0.11 | 1.00 ± 0.07 | 0.82 ± 0.11 | 0.72 ± 0.03 | 1.49 ± 0.35 | 0.071 | 0.193 | 0.825 |
| **Glu.g.75** | *µmol organ^-1^* | 0.70 ± 0.06^ab^ | 0.55 ± 0.12^b^ | 0.91 ± 0.11^ab^ | 0.85 ± 0.14^ab^ | 1.19 ± 0.18^a^ | 0.87 ± 0.05^ab^ | 0.88 ± 0.05^ab^ | 0.99 ± 0.07^ab^ | 0.92 ± 0.09^ab^ | 1.24 ± 0.18^a^ | 0.004 | 0.075 | 0.719 |
| **Glu.l.75** | *µmol organ^-1^* | 1.04 ± 0.10 | 0.81 ± 0.25 | 1.02 ± 0.19 | 0.91 ± 0.14 | 1.26 ± 0.29 | 0.93 ± 0.08 | 1.09 ± 0.14 | 1.04 ± 0.03 | 0.97 ± 0.03 | 1.17 ± 0.19 | 0.468 | 0.749 | 0.774 |
| **aa.b.65** | *µmol organ^-1^* | 5.90 ± 0.93^ac^ | 5.87 ± 0.33^ac^ | 7.93 ± 0.89^ab^ | 5.83 ± 0.27^ac^ | 8.57 ± 1.47^a^ | 5.11 ± 1.50^ac^ | 3.78 ± 0.31^bc^ | 4.83 ± 0.42^ac^ | 3.60 ± 0.87^c^ | 5.04 ± 0.26^ac^ | 0.089 | 0.000 | 0.565 |
| **aa.s.65** | *µmol organ^-1^* | 7.01 ± 0.39 | 5.27 ± 0.21 | 7.83 ± 0.72 | 5.71 ± 0.50 | 6.78 ± 0.17 | 6.81 ± 0.26 | 5.55 ± 0.68 | 5.89 ± 0.27 | 7.18 ± 1.04 | 7.54 ± 0.98 | 0.065 | 0.851 | 0.098 |
| **aa.p.65** | *µmol organ^-1^* | 6.94 ± 2.08 | 10.90 ± 1.14 | 14.10 ± 2.82 | 8.79 ± 1.36 | 11.00 ± 1.46 | 8.00 ± 1.28 | 14.2 ± 1.22 | 9.51 ± 1.09 | 10.50 ± 1.17 | 13.00 ± 1.25 | 0.022 | 0.500 | 0.154 |
| **aa.a.65** | *µmol organ^-1^* | 5.55 ± 1.24 | 4.06 ± 0.15 | 3.71 ± 0.70 | 3.94 ± 0.09 | 5.71 ± 0.91 | 3.91 ± 0.58 | 5.24 ± 0.67 | 3.28 ± 0.13 | 2.92 ± 0.25 | 4.24 ± 0.46 | 0.059 | 0.105 | 0.202 |
| **aa.g.65** | *µmol organ^-1^* | 2.16 ± 0.29^e^ | 3.21 ± 0.46^de^ | 3.97 ± 0.49^cde^ | 3.03 ± 0.29^de^ | 6.98 ± 0.80^ab^ | 3.97 ± 0.54^cde^ | 5.59 ± 0.40^bc^ | 4.56 ± 0.14^cd^ | 4.84 ± 0.40^bd^ | 9.06 ± 0.48^a^ | 0.000 | 0.000 | 0.383 |
| **aa.l.65** | *µmol organ^-1^* | 4.11 ± 0.80^bc^ | 3.15 ± 0.54^c^ | 4.39 ± 0.45^ac^ | 3.51 ± 0.69^c^ | 7.26 ± 0.79^ab^ | 5.06 ± 0.69^ac^ | 7.34 ± 0.29^a^ | 4.15 ± 0.41^bc^ | 5.22 ± 0.90^ac^ | 6.17 ± 0.51^ac^ | 0.005 | 0.012 | 0.005 |


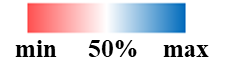
**Supplementary Table S1**. Continued.

| **Variable** | **Units** | **Irrigated** | | | | | **Rainfed** | | | | | ***P*-value** | | |
| --- | --- | --- | --- | --- | --- | --- | --- | --- | --- | --- | --- | --- | --- | --- |
|  |  | **MEX** | **EUR** | **DRI** | **KNI** | **HAR** | **MEX** | **EUR** | **DRI** | **KNI** | **HAR** | **G** | **W** | **G×W** |
| **aa.b.75** | *µmol organ^-1^* | 4.06 ± 0.34^ab^ | 5.07 ± 0.25^ab^ | 6.70 ± 0.53^ab^ | 5.35 ± 0.57^ab^ | 7.43 ± 1.84^a^ | 3.30 ± 0.28^ab^ | 2.87 ± 0.19^b^ | 4.92 ± 0.61^ab^ | 2.46 ± 0.78^b^ | 4.22 ± 1.58^ab^ | 0.042 | 0.001 | 0.668 |
| **aa.s.75** | *µmol organ^-1^* | 4.31 ± 0.68 | 3.34 ± 0.51 | 5.17 ± 0.75 | 4.09 ± 0.55 | 4.05 ± 0.02 | 5.71 ± 0.05 | 3.80 ± 0.20 | 5.33 ± 0.64 | 5.18 ± 0.63 | 4.62 ± 0.97 | 0.073 | 0.060 | 0.829 |
| **aa.p.75** | *µmol organ^-1^* | 11.2 ± 2.0 | 15.3 ± 1.5 | 16.1 ± 1.9 | 17.3 ± 2.2 | 14.5 ± 2.3 | 10.2 ± 1.1 | 9.3 ± 0.6 | 12.3 ± 1.6 | 9.5 ± 2.2 | 9.0 ± 2.6 | 0.408 | 0.001 | 0.477 |
| **aa.a.75** | *µmol organ^-1^* | 5.64 ± 0.87 | 5.51 ± 1.15 | 3.50 ± 0.63 | 4.70 ± 1.07 | 6.54 ± 0.85 | 4.18 ± 0.38 | 5.04 ± 0.74 | 3.00 ± 0.43 | 3.14 ± 0.15 | 6.13 ± 1.00 | 0.008 | 0.094 | 0.895 |
| **aa.g.75** | *µmol organ^-1^* | 3.02 ± 0.12^ab^ | 2.10 ± 0.38^b^ | 3.53 ± 0.72^ab^ | 5.30 ± 0.19^a^ | 3.48 ± 0.53^ab^ | 4.87 ± 0.17^a^ | 5.24 ± 0.16^a^ | 3.25 ± 0.18^ab^ | 4.76 ± 0.62^a^ | 4.99 ± 0.87^a^ | 0.024 | 0.001 | 0.004 |
| **aa.l.75** | *µmol organ^-1^* | 4.98 ± 0.48^bc^ | 3.48 ± 0.99^c^ | 4.00 ± 0.86^bc^ | 5.42 ± 0.74^bc^ | 5.15 ± 1.11^bc^ | 6.15 ± 0.49^ac^ | 9.51 ± 0.83^a^ | 4.35 ± 0.21^bc^ | 7.43 ± 0.32^ab^ | 6.52 ± 1.05^ac^ | 0.044 | 0.000 | 0.012 |
| **Prot.b.65** | *mg organ^-1^* | 5.35 ± 0.71^ac^ | 5.79 ± 0.83^ac^ | 7.31 ± 0.89^ab^ | 4.91 ± 0.11^bc^ | 8.59 ± 0.10^a^ | 4.50 ± 1.38^bc^ | 3.43 ± 0.11^c^ | 4.59 ± 0.27^bc^ | 3.28 ± 0.77^c^ | 4.46 ± 0.50^bc^ | 0.014 | 0.000 | 0.221 |
| **Prot.s.65** | *mg organ^-1^* | 5.48 ± 0.47^ab^ | 4.45 ± 0.22^ab^ | 5.56 ± 0.23^a^ | 4.85 ± 0.27^ab^ | 5.28 ± 0.18^ab^ | 4.87 ± 0.10^ab^ | 3.99 ± 0.15^b^ | 4.62 ± 0.27^ab^ | 4.40 ± 0.63^ab^ | 4.88 ± 0.24^ab^ | 0.028 | 0.009 | 0.908 |
| **Prot.p.65** | *mg organ^-1^* | 8.44 ± 2.18^ab^ | 9.46 ± 0.64^ab^ | 10.80 ± 0.86^a^ | 10.90 ± 0.76^a^ | 6.54 ± 0.29^ab^ | 6.48 ± 1.41^ab^ | 5.66 ± 0.15^b^ | 6.72 ± 0.67^ab^ | 6.54 ± 1.02^ab^ | 5.43 ± 0.26^b^ | 0.074 | 0.000 | 0.430 |
| **Prot.a.65** | *mg organ^-1^* | 5.50 ± 1.02^a^ | 4.02 ± 0.22^ab^ | 3.83 ± 0.71^ab^ | 4.11 ± 0.69^ab^ | 5.16 ± 0.53^ab^ | 4.19 ± 0.13^ab^ | 4.38 ± 0.32^ab^ | 4.04 ± 0.16^ab^ | 2.68 ± 0.14^b^ | 3.86 ± 0.29^ab^ | 0.087 | 0.044 | 0.223 |
| **Prot.g.65** | *mg organ^-1^* | 2.48 ± 0.31^ac^ | 1.67 ± 0.06^c^ | 2.35 ± 0.23^ac^ | 2.01 ± 0.20^bc^ | 2.87 ± 0.14^a^ | 2.02 ± 0.10^bc^ | 2.07 ± 0.06^ac^ | 2.40 ± 0.10^ac^ | 1.80 ± 0.06^bc^ | 2.64 ± 0.22^ab^ | 0.000 | 0.422 | 0.149 |
| **Prot.l.65** | *mg organ^-1^* | 3.44 ± 0.51^ab^ | 2.21 ± 0.14^bc^ | 2.75 ± 0.28^ac^ | 2.62 ± 0.30^ac^ | 3.51 ± 0.09^a^ | 2.39 ± 0.21^ac^ | 2.39 ± 0.05^ac^ | 2.69 ± 0.19^ac^ | 1.99 ± 0.18^c^ | 2.62 ± 0.24^ac^ | 0.017 | 0.006 | 0.101 |
| **Prot.b.75** | *mg organ^-1^* | 5.02 ± 0.30^bcd^ | 5.50 ± 0.28^ac^ | 8.46 ± 0.59^a^ | 5.10 ± 0.24^ad^ | 6.83 ± 0.97^ab^ | 2.25 ± 0.34^cd^ | 2.94 ± 0.27^cd^ | 3.57 ± 0.10^cd^ | 2.05 ± 0.62^d^ | 4.15 ± 1.60^bcd^ | 0.005 | 0.000 | 0.429 |
| **Prot.s.75** | *mg organ^-1^* | 4.04 ± 0.49^ab^ | 3.44 ± 0.34^b^ | 5.97 ± 0.59^a^ | 3.96 ± 0.51^ab^ | 4.72 ± 0.27^ab^ | 3.67 ± 0.36^ab^ | 3.64 ± 0.11^ab^ | 5.11 ± 0.36^ab^ | 3.42 ± 0.54^b^ | 5.24 ± 0.96^ab^ | 0.002 | 0.515 | 0.646 |
| **Prot.p.75** | *mg organ^-1^* | 8.42 ± 0.89^ab^ | 8.23 ± 0.68^ab^ | 11.00 ± 0.67^a^ | 9.48 ± 1.17^ab^ | 10.8 ± 1.63^a^ | 4.93 ± 0.33^b^ | 5.55 ± 0.31^b^ | 7.75 ± 0.61^ab^ | 5.66 ± 1.06^b^ | 7.02 ± 1.13^ab^ | 0.030 | 0.000 | 0.969 |
| **Prot.a.75** | *mg organ^-1^* | 3.52 ± 0.24^ab^ | 4.30 ± 1.00^ab^ | 2.94 ± 0.39^ab^ | 3.41 ± 0.33^ab^ | 5.80 ± 0.92^ab^ | 3.55 ± 0.29^ab^ | 4.36 ± 0.19^ab^ | 3.74 ± 0.46^ab^ | 2.79 ± 0.13^b^ | 6.40 ± 1.55^a^ | 0.002 | 0.701 | 0.865 |
| **Prot.g.75** | *mg organ^-1^* | 1.84 ± 0.08^b^ | 1.62 ± 0.26^b^ | 2.08 ± 0.22^ab^ | 1.88 ± 0.39^b^ | 2.77 ± 0.10^ab^ | 2.04 ± 0.10^ab^ | 2.10 ± 0.13^ab^ | 2.49 ± 0.18^ab^ | 1.91 ± 0.14^b^ | 3.33 ± 0.60^a^ | 0.001 | 0.060 | 0.862 |
| **Prot.l.75** | *mg organ^-1^* | 2.30 ± 0.01 | 1.75 ± 0.36 | 2.32 ± 0.28 | 1.71 ± 0.32 | 2.79 ± 0.43 | 2.02 ± 0.15 | 2.33 ± 0.07 | 2.34 ± 0.10 | 1.87 ± 0.11 | 2.94 ± 0.56 | 0.020 | 0.512 | 0.699 |
| **Chla.b.65** | *mg organ^-1^* | 0.865 ± 0.123^ac^ | 0.906 ± 0.078^ab^ | 0.889 ± 0.098^ab^ | 0.823 ± 0.067^ac^ | 0.966 ± 0.100^a^ | 0.525 ± 0.149^ac^ | 0.541 ± 0.033^ac^ | 0.692 ± 0.019^ac^ | 0.428 ± 0.092^c^ | 0.495 ± 0.068^bc^ | 0.494 | 0.000 | 0.660 |
| **Chla.s.65** | *mg organ^-1^* | 0.891 ± 0.063 | 0.939 ± 0.039 | 1.110 ± 0.027 | 0.827 ± 0.040 | 0.984 ± 0.160 | 0.786 ± 0.039 | 1.140 ± 0.179 | 1.070 ± 0.108 | 0.951 ± 0.089 | 1.290 ± 0.187 | 0.057 | 0.181 | 0.340 |
| **Chla.p.65** | *mg organ^-1^* | 0.872 ± 0.191 | 0.949 ± 0.064 | 1.220 ± 0.221 | 1.070 ± 0.015 | 0.568 ± 0.040 | 0.748 ± 0.102 | 1.140 ± 0.220 | 0.722 ± 0.042 | 0.700 ± 0.044 | 0.869 ± 0.152 | 0.155 | 0.241 | 0.029 |
| **Chla.a.65** | *mg organ^-1^* | 0.855 ± 0.175^a^ | 0.650 ± 0.060^ab^ | 0.611 ± 0.128^ab^ | 0.652 ± 0.083^ab^ | 0.671 ± 0.061^ab^ | 0.455 ± 0.008^ab^ | 0.624 ± 0.052^ab^ | 0.503 ± 0.035^ab^ | 0.320 ± 0.032^b^ | 0.427 ± 0.070^b^ | 0.287 | 0.000 | 0.195 |
| **Chla.g.65** | *mg organ^-1^* | 0.260 ± 0.033^ac^ | 0.188 ± 0.002^cd^ | 0.307 ± 0.021^a^ | 0.241 ± 0.017^ad^ | 0.293 ± 0.021^ab^ | 0.189 ± 0.011^cd^ | 0.211 ± 0.019^bcd^ | 0.267 ± 0.013^ac^ | 0.159 ± 0.012^d^ | 0.293 ± 0.017^ab^ | 0.000 | 0.008 | 0.040 |
| **Chla.l.65** | *mg organ^-1^* | 0.417 ± 0.066^a^ | 0.289 ± 0.026^ab^ | 0.380 ± 0.035^a^ | 0.353 ± 0.026^ab^ | 0.381 ± 0.041^a^ | 0.258 ± 0.019^ab^ | 0.311 ± 0.026^ab^ | 0.370 ± 0.032^a^ | 0.194 ± 0.016^b^ | 0.294 ± 0.025^ab^ | 0.066 | 0.002 | 0.044 |
| **Chla.b.75** | *mg organ^-1^* | 0.621 ± 0.046^ab^ | 0.734 ± 0.063^ab^ | 1.170 ± 0.033^a^ | 0.828 ± 0.140^ab^ | 1.180 ± 0.417^a^ | 0.345 ± 0.077^b^ | 0.543 ± 0.156^ab^ | 0.462 ± 0.057^ab^ | 0.204 ± 0.052^b^ | 0.384 ± 0.151^ab^ | 0.178 | 0.000 | 0.271 |
| **Chla.s.75** | *mg organ^-1^* | 0.765 ± 0.051 | 0.753 ± 0.054 | 1.001 ± 0.066 | 0.723 ± 0.097 | 1.450 ± 0.365 | 0.893 ± 0.122 | 0.946 ± 0.136 | 1.220 ± 0.342 | 0.890 ± 0.225 | 0.962 ± 0.085 | 0.117 | 0.833 | 0.363 |
| **Chla.p.75** | *mg organ^-1^* | 1.82 ± 0.13^ab^ | 2.06 ± 0.30^ab^ | 2.35 ± 0.47^ab^ | 2.02 ± 0.03^ab^ | 2.99 ± 0.85^a^ | 0.89 ± 0.03^b^ | 1.29 ± 0.46^ab^ | 1.38 ± 0.26^ab^ | 1.33 ± 0.42^ab^ | 1.50 ± 0.24^ab^ | 0.290 | 0.001 | 0.869 |
| **Chla.a.75** | *mg organ^-1^* | 0.475 ± 0.018 | 0.581 ± 0.124 | 0.479 ± 0.035 | 0.527 ± 0.050 | 0.644 ± 0.183 | 0.441 ± 0.052 | 0.476 ± 0.062 | 0.468 ± 0.100 | 0.252 ± 0.044 | 0.647 ± 0.163 | 0.155 | 0.193 | 0.620 |
| **Chla.g.75** | *mg organ^-1^* | 0.186 ± 0.005^ab^ | 0.157 ± 0.020^b^ | 0.228 ± 0.031^ab^ | 0.179 ± 0.022^ab^ | 0.276 ± 0.031^ab^ | 0.185 ± 0.021^ab^ | 0.209 ± 0.008^ab^ | 0.247 ± 0.037^ab^ | 0.137 ± 0.011^b^ | 0.313 ± 0.060^a^ | 0.001 | 0.486 | 0.533 |
| **Chla.l.75** | *mg organ^-1^* | 0.244 ± 0.004 | 0.193 ± 0.034 | 0.269 ± 0.038 | 0.193 ± 0.020 | 0.301 ± 0.041 | 0.204 ± 0.007 | 0.249 ± 0.015 | 0.257 ± 0.042 | 0.166 ± 0.019 | 0.300 ± 0.056 | 0.014 | 0.819 | 0.614 |
| **Chlb.b.65** | *mg organ^-1^* | 0.351 ± 0.036 | 0.298 ± 0.035 | 0.356 ± 0.049 | 0.282 ± 0.018 | 0.397 ± 0.045 | 0.235 ± 0.051 | 0.386 ± 0.040 | 0.425 ± 0.062 | 0.268 ± 0.041 | 0.245 ± 0.024 | 0.092 | 0.360 | 0.031 |
| **Chlb.s.65** | *mg organ^-1^* | 0.493 ± 0.009^c^ | 0.628 ± 0.045^bc^ | 0.706 ± 0.060^bc^ | 0.442 ± 0.024^c^ | 0.790 ± 0.266^ac^ | 0.643 ± 0.079^bc^ | 1.310 ± 0.218^ab^ | 1.050 ± 0.203^ac^ | 0.985 ± 0.016^ac^ | 1.520 ± 0.293^a^ | 0.016 | 0.000 | 0.371 |
| **Chlb.p.65** | *mg organ^-1^* | 0.653 ± 0.168^ab^ | 0.748 ± 0.044^ab^ | 0.917 ± 0.122^ab^ | 0.797 ± 0.029^ab^ | 0.599 ± 0.006^b^ | 0.651 ± 0.105^ab^ | 1.290 ± 0.275^a^ | 0.518 ± 0.008^b^ | 0.740 ± 0.053^ab^ | 1.170 ± 0.223^ab^ | 0.097 | 0.146 | 0.008 |
| **Chlb.a.65** | *mg organ^-1^* | 0.327 ± 0.046^ab^ | 0.247 ± 0.025^bc^ | 0.219 ± 0.033^bc^ | 0.219 ± 0.029^bc^ | 0.312 ± 0.019^ab^ | 0.211 ± 0.007^bc^ | 0.397 ± 0.025^a^ | 0.246 ± 0.020^bc^ | 0.142 ± 0.005^c^ | 0.285 ± 0.0252^ab^ | 0.000 | 0.597 | 0.001 |
| **Chlb.g.65** | *mg organ^-1^* | 0.178 ± 0.0215^bc^ | 0.128 ± 0.004^bc^ | 0.176 ± 0.014^bc^ | 0.141 ± 0.015^bc^ | 0.186 ± 0.018^bc^ | 0.140 ± 0.016^bc^ | 0.181 ± 0.019^bc^ | 0.188 ± 0.007^b^ | 0.114 ± 0.002^c^ | 0.262 ± 0.015^a^ | 0.000 | 0.110 | 0.003 |
| **Chlb.l.65** | *mg organ^-1^* | 0.279 ± 0.046^a^ | 0.200 ± 0.014^ab^ | 0.246 ± 0.018^ab^ | 0.207 ± 0.025^ab^ | 0.255 ± 0.031^ab^ | 0.206 ± 0.025^ab^ | 0.318 ± 0.033^a^ | 0.316 ± 0.039^a^ | 0.137 ± 0.008^b^ | 0.283 ± 0.022^a^ | 0.008 | 0.417 | 0.009 |
| **Chlb.b.75** | *mg organ^-1^* | 0.294 ± 0.046 | 0.415 ± 0.084 | 0.511 ± 0.050 | 0.328 ± 0.038 | 1.160 ± 0.769 | 0.333 ± 0.159 | 0.589 ± 0.274 | 0.418 ± 0.129 | 0.175 ± 0.065 | 0.212 ± 0.060 | 0.542 | 0.265 | 0.298 |
| **Chlb.s.75** | *mg organ^-1^* | 0.653 ± 0.123 | 0.731 ± 0.129 | 0.730 ± 0.0816 | 0.520 ± 0.0971 | 1.690 ± 0.571 | 1.090 ± 0.335 | 1.370 ± 0.241 | 1.420 ± 0.566 | 1.160 ± 0.326 | 1.020 ± 0.124 | 0.510 | 0.097 | 0.191 |
| **Chlb.p.75** | *mg organ^-1^* | 2.13 ± 0.41 | 2.27 ± 0.53 | 2.22 ± 0.66 | 2.00 ± 0.10 | 3.48 ± 0.98 | 0.96 ± 0.09 | 1.57 ± 0.69 | 1.53 ± 0.33 | 1.60 ± 0.43 | 2.14 ± 0.57 | 0.219 | 0.021 | 0.903 |
| **Chlb.a.75** | *mg organ^-1^* | 0.229 ± 0.007 | 0.267 ± 0.032 | 0.187 ± 0.014 | 0.205 ± 0.019 | 0.392 ± 0.087 | 0.250 ± 0.035 | 0.331 ± 0.051 | 0.319 ± 0.093 | 0.169 ± 0.022 | 0.437 ± 0.102 | 0.009 | 0.223 | 0.680 |
| **Chlb.g.75** | *mg organ^-1^* | 0.132 ± 0.0057^b^ | 0.115 ± 0.006^b^ | 0.140 ± 0.013^b^ | 0.121 ± 0.018^b^ | 0.209 ± 0.017^ab^ | 0.138 ± 0.007^b^ | 0.171 ± 0.016^ab^ | 0.197 ± 0.042^ab^ | 0.132 ± 0.008^b^ | 0.243 ± 0.035^a^ | 0.001 | 0.019 | 0.619 |
| **Chlb.l.75** | *mg organ^-1^* | 0.175 ± 0.000 | 0.145 ± 0.012 | 0.167 ± 0.015 | 0.128 ± 0.020 | 0.253 ± 0.036 | 0.171 ± 0.013 | 0.255 ± 0.024 | 0.244 ± 0.070 | 0.165 ± 0.025 | 0.275 ± 0.068 | 0.044 | 0.046 | 0.547 |
| **Chltot.b.65** | *mg organ^-1^* | 1.56 ± 0.34^ab^ | 1.21 ± 0.06^ab^ | 1.11 ± 0.12^ab^ | 1.15 ± 0.14^ab^ | 1.31 ± 0.13^ab^ | 1.04 ± 0.03^ab^ | 1.57 ± 0.11^a^ | 1.40 ± 0.19^ab^ | 0.81 ± 0.05^b^ | 0.97 ± 0.08^ab^ | 0.099 | 0.270 | 0.021 |
| **Chltot.s.65** | *mg organ^-1^* | 0.73 ± 0.08^b^ | 0.69 ± 0.06^b^ | 1.15 ± 0.12^ab^ | 0.64 ± 0.07^b^ | 1.00 ± 0.26^ab^ | 0.67 ± 0.08^b^ | 1.41 ± 0.27^ab^ | 1.44 ± 0.24^ab^ | 0.93 ± 0.04^b^ | 1.80 ± 0.20^a^ | 0.001 | 0.001 | 0.092 |
| **Chltot.p.65** | *mg organ^-1^* | 0.595 ± 0.079^ab^ | 0.407 ± 0.058^b^ | 0.612 ± 0.116^ab^ | 0.449 ± 0.056^b^ | 0.513 ± 0.029^b^ | 0.549 ± 0.113^ab^ | 1.070 ± 0.187^a^ | 0.610 ± 0.089^ab^ | 0.406 ± 0.024^b^ | 0.905 ± 0.168^ab^ | 0.056 | 0.009 | 0.008 |
| **Chltot.a.65** | *mg organ^-1^* | 1.18 ± 0.22^a^ | 0.90 ± 0.08^ab^ | 0.83 ± 0.16^ab^ | 0.87 ± 0.11^ab^ | 0.98 ± 0.08^ab^ | 0.67 ± 0.01^ab^ | 1.02 ± 0.08^a^ | 0.75 ± 0.05^ab^ | 0.46 ± 0.0358^b^ | 0.71 ± 0.10^ab^ | 0.098 | 0.003 | 0.057 |
| **Chltot.g.65** | *mg organ^-1^* | 0.439 ± 0.054^abc^ | 0.317 ± 0.006^cd^ | 0.483 ± 0.034^ab^ | 0.382 ± 0.031^bd^ | 0.479 ± 0.038^ab^ | 0.329 ± 0.026^bd^ | 0.392 ± 0.038^bd^ | 0.455 ± 0.020^abc^ | 0.273 ± 0.013^d^ | 0.555 ± 0.031^a^ | 0.000 | 0.359 | 0.012 |
| **Chltot.l.65** | *mg organ^-1^* | 0.696 ± 0.112^a^ | 0.489 ± 0.040^ab^ | 0.626 ± 0.052^ab^ | 0.560 ± 0.050^ab^ | 0.636 ± 0.070^ab^ | 0.465 ± 0.043^ab^ | 0.630 ± 0.059^ab^ | 0.686 ± 0.070^a^ | 0.331 ± 0.023^b^ | 0.577 ± 0.046^ab^ | 0.032 | 0.114 | 0.019 |


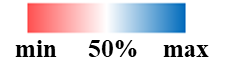
**Supplementary Table S1**. Continued.

| **Variable** | **Units** | **Irrigated** | | | | | **Rainfed** | | | | | ***P*-value** | | |
| --- | --- | --- | --- | --- | --- | --- | --- | --- | --- | --- | --- | --- | --- | --- |
|  |  | **MEX** | **EUR** | **DRI** | **KNI** | **HAR** | **MEX** | **EUR** | **DRI** | **KNI** | **HAR** | **G** | **W** | **G×W** |
| **Chltot.b.75** | *mg organ^-1^* | 0.92 ± 0.09 | 1.15 ± 0.15 | 1.68 ± 0.08 | 1.16 ± 0.18 | 2.34 ± 1.17 | 0.68 ± 0.23 | 1.13 ± 0.43 | 0.88 ± 0.18 | 0.38 ± 0.12 | 0.60 ± 0.21 | 0.409 | 0.014 | 0.321 |
| **Chltot.s.75** | *mg organ^-1^* | 1.42 ± 0.15 | 1.48 ± 0.18 | 1.83 ± 0.11 | 1.24 ± 0.19 | 3.14 ± 0.93 | 1.99 ± 0.46 | 2.32 ± 0.38 | 2.64 ± 0.91 | 2.04 ± 0.55 | 1.98 ± 0.20 | 0.346 | 0.252 | 0.241 |
| **Chltot.p.75** | *mg organ^-1^* | 3.95 ± 0.53 | 4.33 ± 0.82 | 4.57 ± 1.12 | 4.02 ± 0.11 | 6.47 ± 1.81 | 1.85 ± 0.06 | 2.86 ± 1.15 | 2.91 ± 0.58 | 2.93 ± 0.84 | 3.64 ± 0.81 | 0.246 | 0.005 | 0.899 |
| **Chltot.a.75** | *mg organ^-1^* | 0.704 ± 0.024 | 0.848 ± 0.155 | 0.666 ± 0.047 | 0.732 ± 0.067 | 1.040 ± 0.266 | 0.691 ± 0.087 | 0.807 ± 0.111 | 0.787 ± 0.191 | 0.421 ± 0.056 | 1.080 ± 0.258 | 0.049 | 0.687 | 0.673 |
| **Chltot.g.75** | *mg organ^-1^* | 0.318 ± 0.010^ab^ | 0.272 ± 0.024^b^ | 0.368 ± 0.043^ab^ | 0.299 ± 0.040^b^ | 0.485 ± 0.048^ab^ | 0.323 ± 0.028^ab^ | 0.380 ± 0.023^ab^ | 0.443 ± 0.078^ab^ | 0.268 ± 0.018^b^ | 0.556 ± 0.093^a^ | 0.001 | 0.144 | 0.594 |
| **Chltot.l.75** | *mg organ^-1^* | 0.419 ± 0.004 | 0.337 ± 0.046 | 0.437 ± 0.053 | 0.321 ± 0.039 | 0.555 ± 0.072 | 0.375 ± 0.020 | 0.505 ± 0.038 | 0.501 ± 0.112 | 0.332 ± 0.039 | 0.576 ± 0.123 | 0.021 | 0.302 | 0.583 |


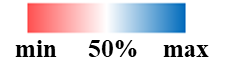
**Supplementary Table S2**. Effect of organ (O), water regime (W) and their interaction (G×W) on C [glucose (Glc), glucose-6-phosphate (Glc6P), fructose (Fru), sucrose (Suc), starch, and malate] and N [glutamate (Glu), total amino acids (aa), proteins (Prot), chlorophylls a (Chla), b (Chlb) and total (Chltotal)] metabolites. The numbers in the traits represent the Zadoks scale when they were measured (Zadoks 65, anthesis; Zadoks 75, mid-grain filling). The values were expressed as total organ content. The means in each row with different letters are statistically different (*P*<0.05; two-way ANOVA, TUKEY test; yellow colour indicates the significance of a factor). The colour scale in the means shows the minimum (red) and maximum (blue) values per trait.

| **Traits** | **Units** | **blade** | | **sheath** | | **peduncle** | | **awn** | | **glume** | | **lemma** | | ***P*-value** | | |
| --- | --- | --- | --- | --- | --- | --- | --- | --- | --- | --- | --- | --- | --- | --- | --- | --- |
|  |  | **irrigated** | **rainfed** | **irrigated** | **rainfed** | **irrigated** | **rainfed** | **irrigated** | **rainfed** | **irrigated** | **rainfed** | **irrigated** | **rainfed** | **organ** | **water** | **O×W** |
| Glc.65 | µmol organ^-1^ | 1.97 ± 0.57^c^ | 2.31 ± 0.36^c^ | 6.78 ± 0.78^c^ | 6.94 ± 0.84^c^ | 66.16 ± 4.87^a^ | 43.63 ± 6.68^b^ | 9.24 ± 1.32^c^ | 7.94 ± 1.37^c^ | 6.68 ± 0.50^c^ | 5.06 ± 0.47^c^ | 12.66 ± 1.31^c^ | 7.72 ± 0.89^c^ | 0.000 | 0.001 | 0.000 |
| Glc.75 | µmol organ^-1^ | 4.65 ± 1.09^b^ | 1.80 ± 0.23^b^ | 3.70 ± 0.66^b^ | 2.58 ± 0.25^b^ | 14.03 ± 2.57^a^ | 6.19 ± 1.56^b^ | 5.81 ± 1.48^b^ | 3.40 ± 0.90^b^ | 3.62 ± 0.47^b^ | 1.96 ± 0.27^b^ | 4.44 ± 0.70^b^ | 2.20 ± 0.35^b^ | 0.000 | 0.000 | 0.038 |
| Glc6P.65 | µmol organ^-1^ | 0.180 ± 0.010^cde^ | 0.068 ± 0.004^h^ | 0.205 ± 0.008^be^ | 0.091 ± 0.003^gh^ | 0.482 ± 0.034^a^ | 0.252 ± 0.023^b^ | 0.248 ± 0.019^bc^ | 0.168 ± 0.009^def^ | 0.151 ± 0.010^eg^ | 0.104 ± 0.005^fgh^ | 0.233 ± 0.014^bd^ | 0.139 ± 0.007^eg^ | 0.000 | 0.000 | 0.000 |
| Glc6P.75 | µmol organ^-1^ | 0.160 ± 0.011^bce^ | 0.076 ± 0.011^f^ | 0.107 ± 0.007^def^ | 0.094 ± 0.009^ef^ | 0.234 ± 0.019^a^ | 0.179 ± 0.021^ac^ | 0.188 ± 0.021^ab^ | 0.166 ± 0.027^acd^ | 0.106 ± 0.006^def^ | 0.119 ± 0.010^cf^ | 0.119 ± 0.007^bcef^ | 0.130 ± 0.010^bcef^ | 0.000 | 0.004 | 0.007 |
| Fru.65 | µmol organ^-1^ | 1.98 ± 0.43^c^ | 1.72 ± 0.29^c^ | 4.33 ± 0.52^c^ | 3.29 ± 0.37^c^ | 35.58 ± 2.44^a^ | 21.59 ± 2.92^b^ | 1.64 ± 0.22^c^ | 1.55 ± 0.21^c^ | 1.91 ± 0.14^c^ | 2.10 ± 0.20^c^ | 5.51 ± 0.53^c^ | 3.64 ± 0.28^c^ | 0.000 | 0.000 | 0.000 |
| Fru.75 | µmol organ^-1^ | 5.40 ± 1.30^b^ | 3.31 ± 0.62^b^ | 7.83 ± 1.78^b^ | 4.94 ± 0.81^b^ | 17.76 ± 2.95^a^ | 7.90 ± 1.64^b^ | 3.43 ± 1.03^b^ | 2.61 ± 0.70^b^ | 2.64 ± 0.52^b^ | 1.96 ± 0.41^b^ | 3.36 ± 0.61^b^ | 2.53 ± 0.39^b^ | 0.000 | 0.000 | 0.003 |
| Suc.65 | µmol organ^-1^ | 16.7 ± 2.0^e^ | 14.7 ± 1.1^e^ | 26.3 ± 1.8^cd^ | 29.2 ± 1.3^bc^ | 38.6 ± 4.3^a^ | 37.1 ± 2.4^ab^ | 15.6 ± 1.3^e^ | 19.1 ± 1.7^de^ | 10.2 ± 1.3^e^ | 13.9 ± 1.1^e^ | 12.8 ± 1.0^e^ | 17.0 ± 1.2^e^ | 0.000 | 0.098 | 0.389 |
| Suc.75 | µmol organ^-1^ | 17.3 ± 1.7^cd^ | 14.9 ± 2.0^d^ | 27.8 ± 1.4^c^ | 28.2 ± 1.9^c^ | 103.0 ± 6.4^a^ | 66.5 ± 4.7^b^ | 13.5 ± 1.6^d^ | 21.2 ± 2.2^cd^ | 9.6 ± 1.1^d^ | 14.0 ± 1.0^d^ | 11.2 ± 0.9^d^ | 16.2 ± 0.8^cd^ | 0.000 | 0.022 | 0.000 |
| starch.65 | µmol organ^-1^ | 2.01 ± 0.43^ad^ | 1.51 ± 0.31^bcd^ | 0.91 ± 0.15^d^ | 1.45 ± 0.27^bcd^ | 2.14 ± 0.21^ad^ | 2.33 ± 0.34^ac^ | 2.49 ± 0.30^ac^ | 3.26 ± 0.43^a^ | 0.83 ± 0.09^d^ | 1.20 ± 0.13^cd^ | 2.75 ± 0.25^ab^ | 3.32 ± 0.38^a^ | 0.000 | 0.060 | 0.341 |
| starch.75 | µmol organ^-1^ | 1.23 ± 0.27^bc^ | 0.98 ± 0.26^be^ | 0.39 ± 0.07^e^ | 0.75 ± 0.09^be^ | 0.48 ± 0.06^de^ | 0.55 ± 0.06^cde^ | 1.31 ± 0.18^b^ | 2.65 ± 0.19^a^ | 0.57 ± 0.07^cde^ | 1.20 ± 0.09^bd^ | 1.12 ± 0.11^be^ | 2.19 ± 0.18^a^ | 0.000 | 0.000 | 0.000 |
| malate.65 | µmol organ^-1^ | 0.971 ± 0.081^b^ | 0.623 ± 0.065^cd^ | 1.508 ± 0.079^a^ | 1.050 ± 0.072^b^ | 1.558 ± 0.128^a^ | 0.740 ± 0.120^bc^ | 0.318 ± 0.048^de^ | 0.187 ± 0.017^e^ | 0.086 ± 0.012^e^ | 0.128 ± 0.014^e^ | 0.313 ± 0.035^de^ | 0.214 ± 0.021^e^ | 0.000 | 0.000 | 0.000 |
| malate.75 | µmol organ^-1^ | 1.046 ± 0.082^ab^ | 0.588 ± 0.077^c^ | 1.204 ± 0.061^a^ | 0.887 ± 0.062^b^ | 0.308 ± 0.039^de^ | 0.200 ± 0.029^de^ | 0.361 ± 0.046^d^ | 0.168 ± 0.041^de^ | 0.097 ± 0.010^e^ | 0.094 ± 0.005^e^ | 0.161 ± 0.018^de^ | 0.132 ± 0.012^e^ | 0.000 | 0.000 | 0.000 |
| Glu.65 | µmol organ^-1^ | 2.50 ± 0.15^b^ | 1.21 ± 0.11^cd^ | 2.13 ± 0.08^b^ | 1.50 ± 0.07^c^ | 3.11 ± 0.27^a^ | 2.28 ± 0.13^b^ | 1.23 ± 0.080^cd^ | 0.86 ± 0.05^d^ | 0.85 ± 0.07^d^ | 0.88 ± 0.04^d^ | 1.28 ± 0.08^cd^ | 1.03 ± 0.06^cd^ | 0.000 | 0.000 | 0.000 |
| Glu.75 | µmol organ^-1^ | 2.19 ± 0.16^b^ | 0.84 ± 0.12^d^ | 1.43 ± 0.10^c^ | 1.03 ± 0.07^cd^ | 3.31 ± 0.23^a^ | 2.12 ± 0.15^b^ | 1.15 ± 0.11^cd^ | 0.95 ± 0.10^cd^ | 0.84 ± 0.08^d^ | 0.98 ± 0.05^cd^ | 1.01 ± 0.09^cd^ | 1.04 ± 0.05^cd^ | 0.000 | 0.000 | 0.000 |
| aa.65 | µmol organ^-1^ | 6.82 ± 0.46^b^ | 4.47 ± 0.36^bc^ | 6.52 ± 0.30^b^ | 6.59 ± 0.34^b^ | 10.35 ± 0.96^a^ | 11.04 ± 0.76^a^ | 4.59 ± 0.37^bc^ | 3.92 ± 0.28^c^ | 3.87 ± 0.48^c^ | 5.61 ± 0.51^bc^ | 4.48 ± 0.46^bc^ | 5.59 ± 0.37^bc^ | 0.000 | 0.745 | 0.001 |
| aa.75 | µmol organ^-1^ | 5.73 ± 0.47^cd^ | 3.55 ± 0.40^d^ | 4.19 ± 0.26^d^ | 4.93 ± 0.29^cd^ | 14.87 ± 0.94^a^ | 10.05 ± 0.74^b^ | 5.18 ± 0.45^cd^ | 4.30 ± 0.39^d^ | 3.49 ± 0.33^d^ | 4.62 ± 0.27^cd^ | 4.60 ± 0.38^cd^ | 6.79 ± 0.52^c^ | 0.000 | 0.026 | 0.000 |
| Prot.65 | mg organ^-1^ | 6.39 ± 0.44^b^ | 4.05 ± 0.32^cd^ | 5.12 ± 0.16^bc^ | 4.55 ± 0.16^c^ | 9.21 ± 0.61^a^ | 6.16 ± 0.35^b^ | 4.52 ± 0.32^c^ | 3.83 ± 0.18^cd^ | 2.28 ± 0.13^e^ | 2.19 ± 0.09^e^ | 2.91 ± 0.18^de^ | 2.41 ± 0.10^e^ | 0.000 | 0.000 | 0.000 |
| Prot.75 | mg organ^-1^ | 6.18 ± 0.41^b^ | 2.99 ± 0.37^cd^ | 4.43 ± 0.29^c^ | 4.22 ± 0.29^c^ | 9.58 ± 0.51^a^ | 6.18 ± 0.40^b^ | 4.00 ± 0.37^c^ | 4.17 ± 0.43^c^ | 2.04 ± 0.14^d^ | 2.38 ± 0.18^d^ | 2.18 ± 0.16^d^ | 2.30 ± 0.14^d^ | 0.000 | 0.000 | 0.000 |
| Chla.65 | mg organ^-1^ | 0.890 ± 0.038^ac^ | 0.537 ± 0.040^de^ | 0.951 ± 0.040^ab^ | 1.047 ± 0.068^a^ | 0.937 ± 0.078^ab^ | 0.835 ± 0.066^bc^ | 0.688 ± 0.048^cd^ | 0.466 ± 0.031^ef^ | 0.258 ± 0.014^fg^ | 0.224 ± 0.014^g^ | 0.364 ± 0.019^eg^ | 0.286 ± 0.018^fg^ | 0.000 | 0.000 | 0.000 |
| Chla.75 | mg organ^-1^ | 0.906 ± 0.097^bc^ | 0.388 ± 0.051^d^ | 0.957 ± 0.100^b^ | 0.983 ± 0.084^b^ | 2.248 ± 0.205^a^ | 1.277 ± 0.133^b^ | 0.541 ± 0.043^cd^ | 0.457 ± 0.049^d^ | 0.205 ± 0.014^d^ | 0.218 ± 0.020^d^ | 0.240 ± 0.016^d^ | 0.235 ± 0.018^d^ | 0.000 | 0.000 | 0.000 |
| Chlb.65 | mg organ^-1^ | 0.337 ± 0.018^d^ | 0.312 ± 0.027^d^ | 0.612 ± 0.058^c^ | 1.102 ± 0.107^a^ | 0.743 ± 0.047^bc^ | 0.873 ± 0.102^ab^ | 0.265 ± 0.017^d^ | 0.256 ± 0.024^d^ | 0.162 ± 0.008^d^ | 0.177 ± 0.014^d^ | 0.237 ± 0.014^d^ | 0.252 ± 0.021^d^ | 0.000 | 0.001 | 0.000 |
| Chlb.75 | mg organ^-1^ | 0.541 ± 0.157^de^ | 0.346 ± 0.072^de^ | 0.864 ± 0.153^cd^ | 1.211 ± 0.139^bc^ | 2.420 ± 0.271^a^ | 1.561 ± 0.204^b^ | 0.256 ± 0.025^e^ | 0.301 ± 0.035^de^ | 0.143 ± 0.010^e^ | 0.176 ± 0.015^e^ | 0.174 ± 0.014^e^ | 0.222 ± 0.021^e^ | 0.000 | 0.184 | 0.000 |
| Chltotal.65 | mg organ^-1^ | 1.27 ± 0.08^a^ | 1.16 ± 0.09^a^ | 0.84 ± 0.074^bc^ | 1.25 ± 0.13^a^ | 0.52 ± 0.035^de^ | 0.71 ± 0.08^be^ | 0.95 ± 0.06^ab^ | 0.72 ± 0.05^bd^ | 0.42 ± 0.02^de^ | 0.40 ± 0.03^e^ | 0.60 ± 0.03^cde^ | 0.54 ± 0.04^cde^ | 0.000 | 0.440 | 0.000 |
| Chltotal.75 | mg organ^-1^ | 1.45 ± 0.24^cd^ | 0.73 ± 0.12^de^ | 1.82 ± 0.25^c^ | 2.19 ± 0.22^bc^ | 4.67 ± 0.47^a^ | 2.84 ± 0.33^b^ | 0.80 ± 0.06^de^ | 0.76 ± 0.08^de^ | 0.35 ± 0.02^e^ | 0.39 ± 0.03^e^ | 0.41 ± 0.03^e^ | 0.46 ± 0.04^e^ | 0.000 | 0.004 | 0.000 |

**Supplementary Fig. S1**. Principal component analysis (PCA) of agronomic, grain quality and physiological traits in five durum wheat varieties (MEX, Mexa; EUR, Euroduro; DRI, Don Ricardo; KNI, Kiko Nick; HAR, Haristide) under rainfed and irrigated conditions. Abbreviations are described throughout the text.


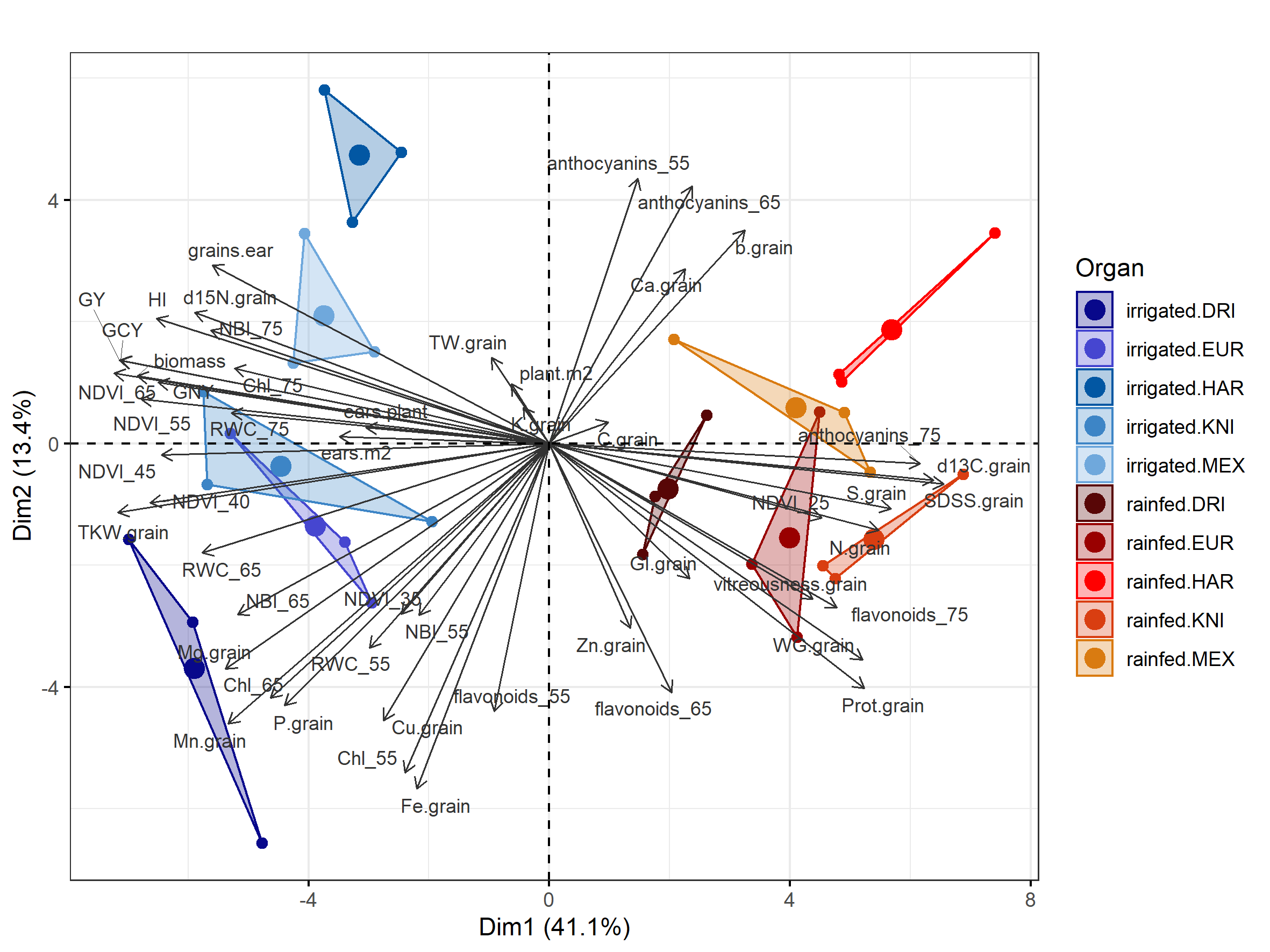


**Supplementary Fig. S2**. Correlation matrix of agronomic components and grain quality traits under **a** irrigated and **b** rainfed conditions. Significant correlations: *, *p*<0.05, **; *p*<0.01; ***, *p*<0.001; blue, positive (*r*>0); red, negative (*r*<0).

**
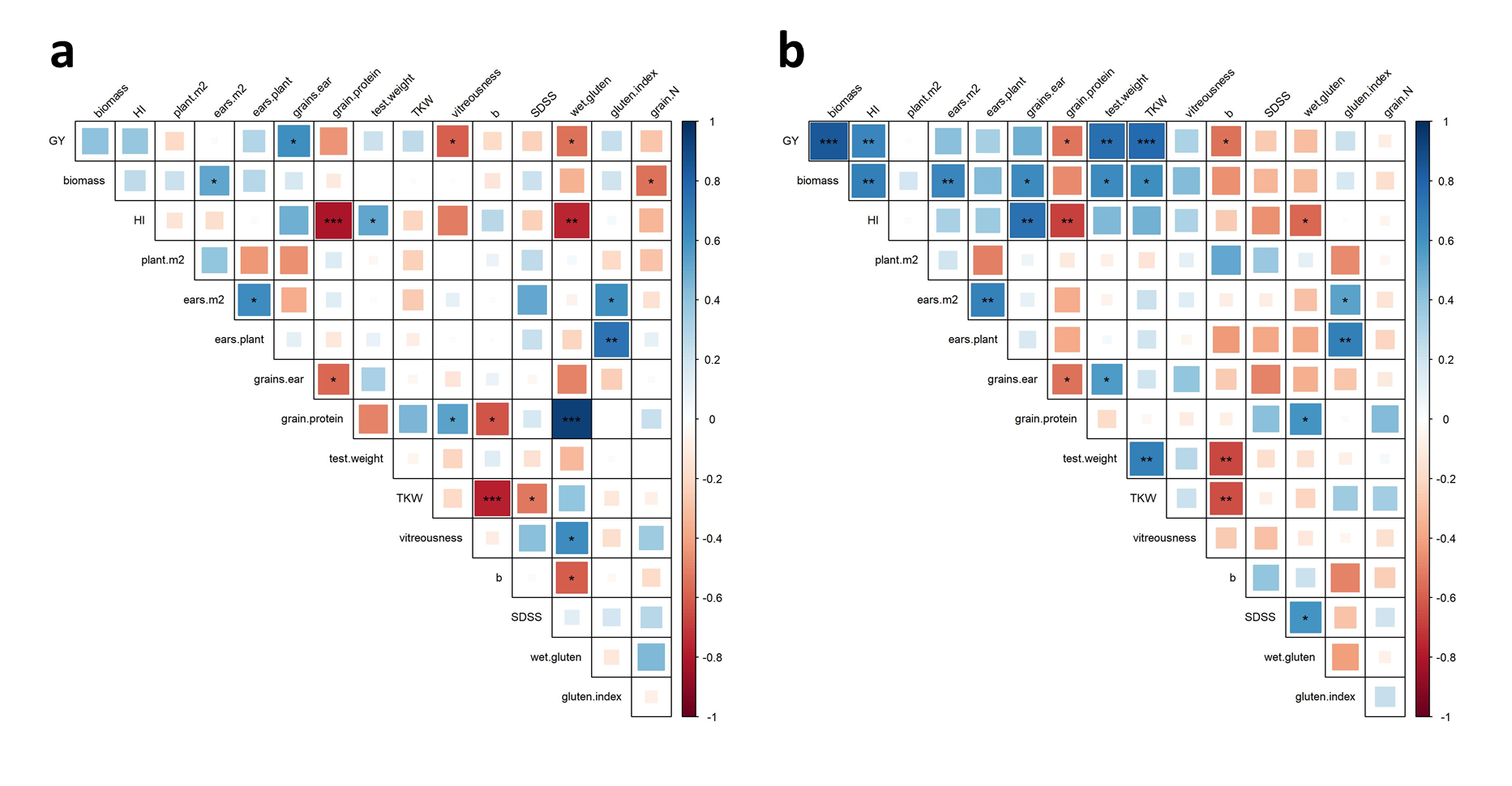
**

**Supplementary Fig. S3**. Percentage (%) of variation in C and N metabolites between mid-grain filling and anthesis (Zadoks 75 and 65, respectively) in six photosynthetic organs. Each box represents the average ± standard deviation of five field-grown durum wheat varieties per organ (blade, sheath, peduncle, awn, glume, and lemma) and water regime (irrigated and rainfed conditions). The different letters differ statistically (*P*<0.05; two-way ANOVA, TUKEY test). Abbreviations: Glc, glucose; Glc6P, glucose-6-phosphate; Fru, fructose; Suc, sucrose; Glu, glutamate; aa, total amino acids; Chltot, total chlorophylls.


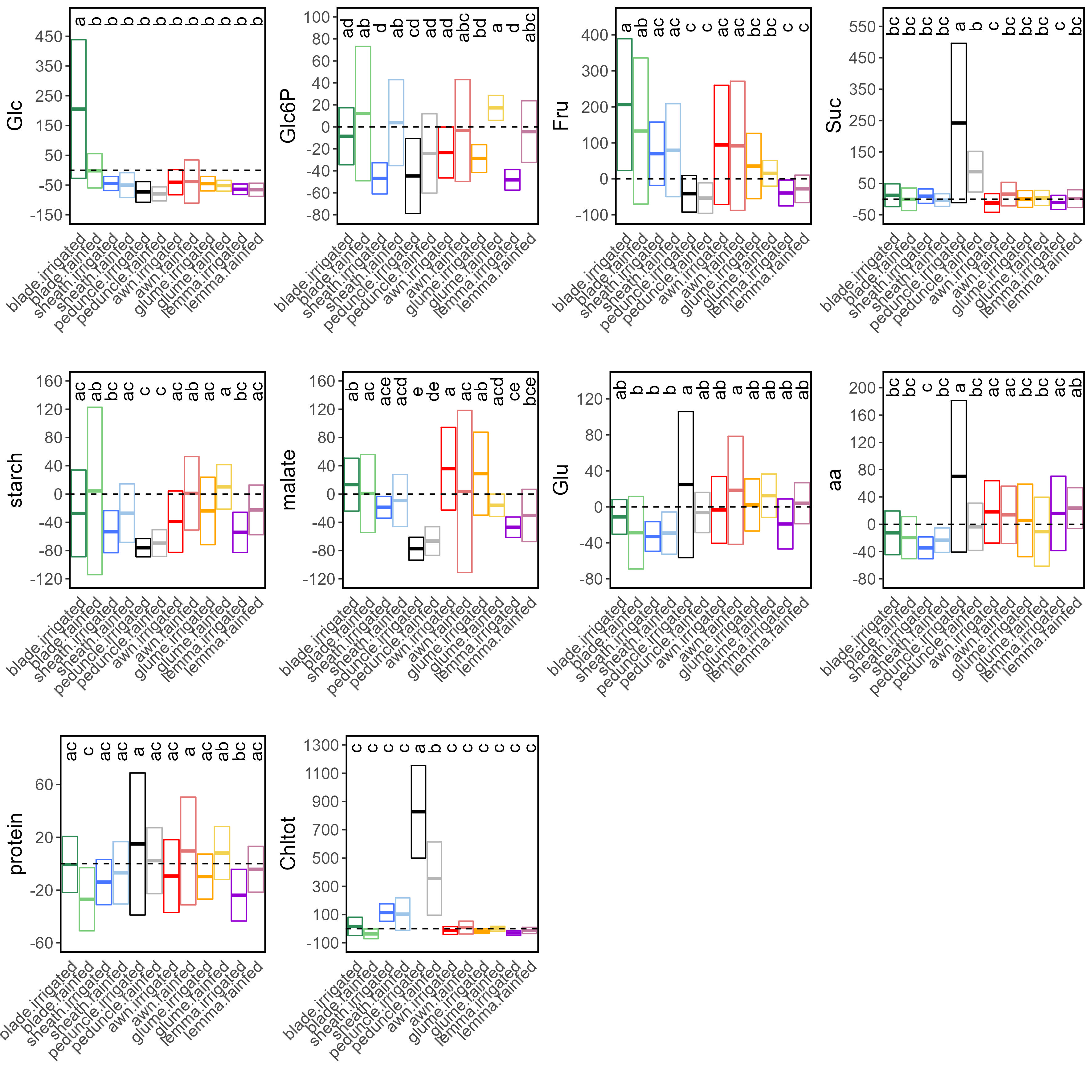

Supplement: Supplementary file 1 — Supplementary file1 (DOCX 4240 kb) [file 425_2023_4115_MOESM1_ESM.docx]
